# *O*-GlcNAcylation Facilitates the Interaction between Keratin 18 and Isocitrate Dehydrogenases and Potentially Influencing Cholangiocarcinoma Progression

**DOI:** 10.1021/acscentsci.4c00163

**Published:** 2024-04-23

**Authors:** Xiangfeng Meng, Yue Zhou, Lei Xu, Limu Hu, Changjiang Wang, Xiao Tian, Xiang Zhang, Yi Hao, Bo Cheng, Jing Ma, Lei Wang, Jialin Liu, Ran Xie

**Affiliations:** † State Key Laboratory of Coordination Chemistry, School of Chemistry and Chemical Engineering, Nanjing University, Nanjing 210023, China; ‡ Department of Gastroenterology, Nanjing Drum Tower Hospital, The Affiliated, Hospital of Nanjing University Medical School, Nanjing 210008, China; § State Key Laboratory of Medical Proteomics, Beijing Proteome Research Center, National Center for Protein Sciences (Beijing), Beijing Institute of Lifeomics, Beijing 102206, China; ∥ Chemistry and Biomedicine Innovation Center (ChemBIC), Nanjing University, Nanjing 210023, China; ⊥ Collaborative Innovation Center of Advanced Microstructures, Nanjing University, Nanjing 210023, China; # College of Chemistry and Molecular Engineering, Peking University, Beijing 100871, China; ▽ School of Pharmaceutical Sciences, Peking University, Beijing 100191, China; ⬢ Beijing National Laboratory for Molecular Sciences, Beijing 100191, China

## Abstract

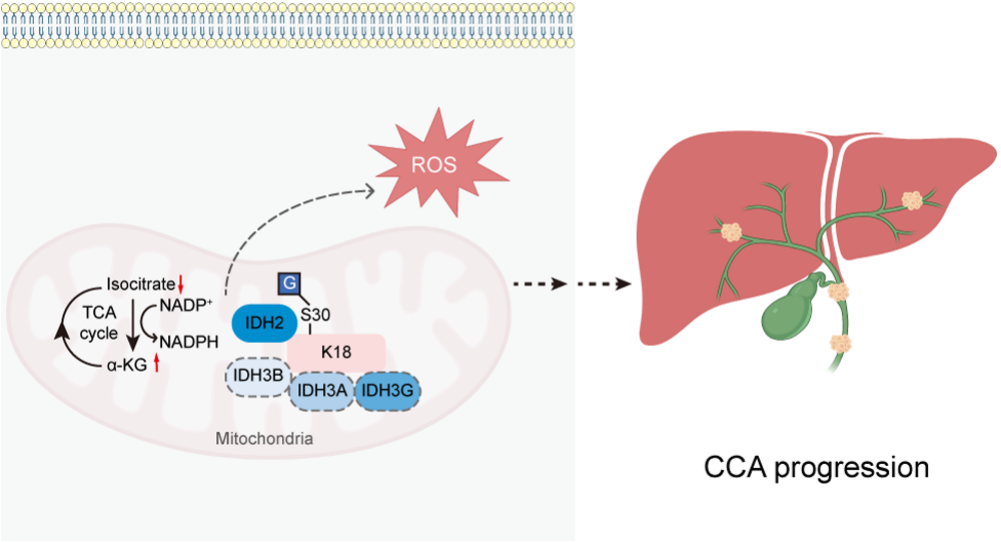

Glycosylation plays a pivotal role in the intricate landscape of human cholangiocarcinoma (CCA), actively participating in key pathophysiological processes driving tumor progression. Among the various glycosylation modifications, *O*-linked β-*N*-acetyl-glucosamine modification (*O*-GlcNAcylation) emerges as a dynamic regulator influencing diverse tumor-associated biological activities. In this study, we employed a state-of-the-art chemical proteomic approach to analyze intact glycopeptides, unveiling the critical role of *O*-GlcNAcylation in orchestrating Keratin 18 (K18) and its interplay with tricarboxylic acid (TCA) cycle enzymes, specifically isocitrate dehydrogenases (IDHs), to propel CCA progression. Our findings shed light on the mechanistic intricacies of *O*-GlcNAcylation, revealing that site-specific modification of K18 on Ser 30 serves as a stabilizing factor, amplifying the expression of cell cycle checkpoints. This molecular event intricately fosters cell cycle progression and augments cellular growth in CCA. Notably, the interaction between *O*-GlcNAcylated K18 and IDHs orchestrates metabolic reprogramming by down-regulating citrate and isocitrate levels while elevating α-ketoglutarate (α-KG). These metabolic shifts further contribute to the overall tumorigenic potential of CCA. Our study thus expands the current understanding of protein *O*-GlcNAcylation and introduces a new layer of complexity to post-translational control over metabolism and tumorigenesis.

## Abstract 2

Synopsis: Unraveling the role of *O*-GlcNAcylation in cholangiocarcinoma (CCA), our study illuminates how it influences Keratin 18, impacting metabolic reprogramming and CCA progression. These findings deepen our understanding of tumorigenesis and post-translational control over metabolism.

## Introduction

Cholangiocarcinoma (CCA), also known as bile duct cancer, constitutes a constellation of malignancies emerging in the biliary tree. CCA is the second most common primary hepatic malignancy, accounting for approximately 15% of all primary liver tumors, and its incidence is increasing worldwide.^1^ CCA is typically asymptomatic in the early stages and difficult to cure at late stages, highly compromises therapeutic options, and results in a bleak prognosis.^2,3^ Like most cancers, in the intricate landscape of CCA progression, alternations of glycosylation greatly impact tumor pathogenesis and progression.^4^ Numerous glycans and glycoconjugates (e.g., glycoproteins, glycolipids) with altered expressions have been identified as tumor markers for diagnosis and prognostic prediction of CCA.^5−7^ Furthermore, certain distinctive glycan modifications appear to be correlated with the short survival of the CCA patients and played pivotal roles in the proliferation, migration, invasion, and chemoresistance of CCA cells.^8−10^ Therefore, functional elucidation of glycosylation in CCA has become the subject of intense scientific efforts.

**Figure 1:**
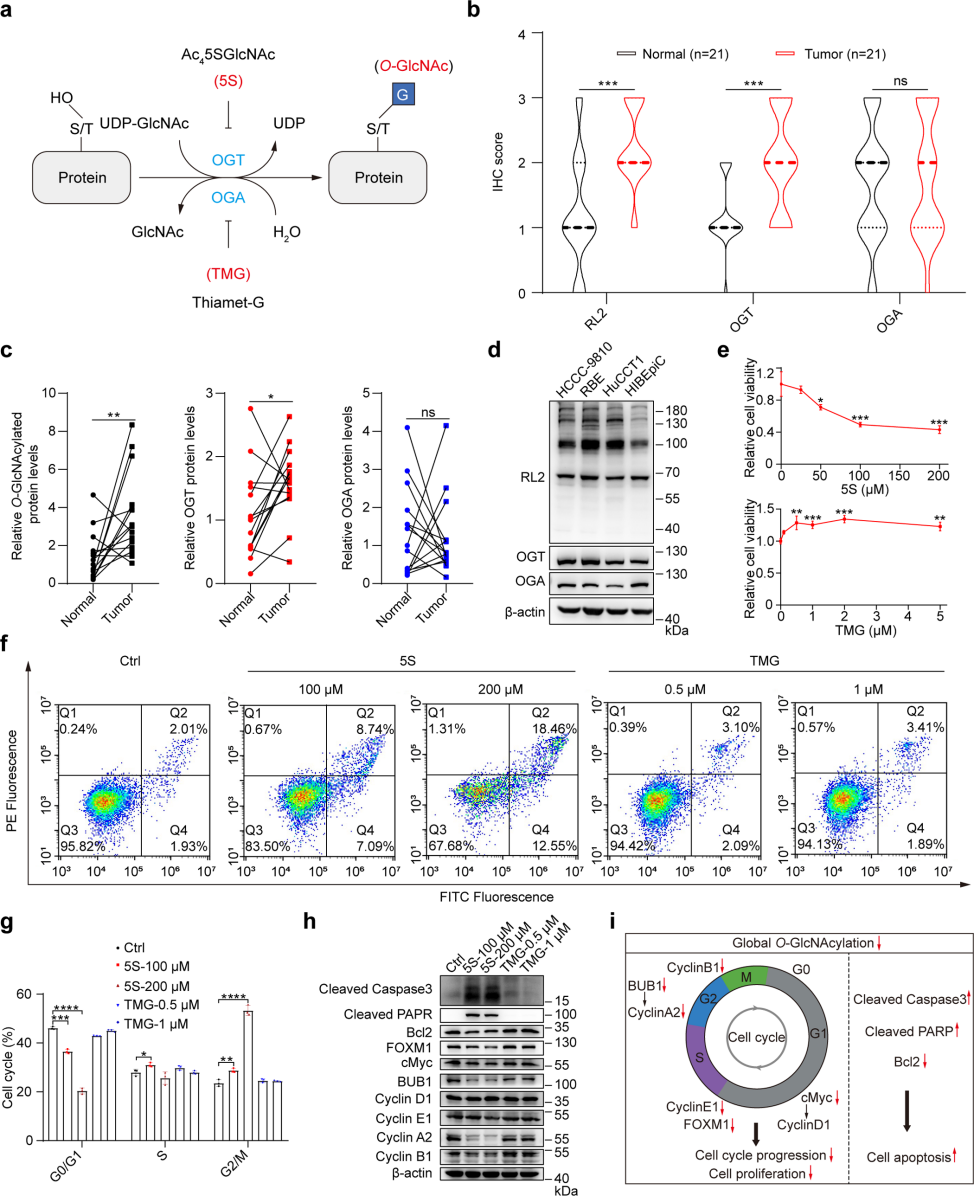
*O*-GlcNAcylation promotes CCA cell proliferation. (a) Schematic of protein *O*-GlcNAcylation processes and inhibition. A pair of enzymes, OGT and OGA, catalyze the addition/removal of single *O*-GlcNAc, respectively. Ac_4_5SGlcNAc (5S) is an inhibitor of OGT, and Thiamet-G (TMG) is an inhibitor of OGA. (b) Analysis of IHC staining scores of RL2 (anti-*O*-GlcNAc), OGT, and OGA in 21 pairs of CCA tumor tissues and adjacent normal bile ducts. Level of staining: 0, negative; 1, weakly positive; 2, positive; and 3, strongly positive. (c) Densitometric analysis of *O*-GlcNAcylated protein, OGT, and OGA levels from 15 pairs of CCA tumor tissues and adjacent normal tissues by Western blot analysis. (d) *O*-GlcNAcylated protein, OGT, and OGA levels of three CCA cell lines and the Human Intrahepatic Biliary Epithelial Cell (HIBEpiC) control cell line by Western blot analysis. Equal loading was confirmed using β-actin. (e) Cytotoxicity assay of 5S- or TMG-treated HuCCT1 cells. The cells were treated with 5S or TMG at various concentrations for 48 h. (f) Cell apoptosis assay of 5S- or TMG-treated HuCCT1 cells. The bivariate density plot in flow cytometry indicated the cell population of early apoptotic cells (FITC^+^/PE^–^) and late apoptotic cells (FITC^+^/PE^+^). (g) Quantitative analysis of cell cycle distribution for 5S- or TMG-treated HuCCT1 cells. (h) Cell cycle and apoptosis marker analysis of 5S- or TMG-treated HuCCT1 cells by Western blot. Protein levels of cleaved Caspase3, cleaved PARP (apoptotic markers); Bcl2 (antiapoptotic marker); cMyc, Cyclin D1, FOXM1, Cyclin E1(G1/S transition markers); and BUB1, Cyclin A2, Cyclin B1 (G2/M transition markers) were analyzed. Equal loading was confirmed using β-actin. (i) Putative schematic model of *O*-GlcNAcylation regulation in CCA progression. CCA, cholangiocarcinoma; *O*-GlcNAc, β-*O*-linked *N*-acetylglucosamine; OGT, *O*-GlcNAc transferase; and OGA, *O*-GlcNAcase. Data were shown as the mean ± standard deviation (SD); statistical significance was determined by Student’s *t* tests (two-tailed, *P < 0.05, **P < 0.01, ***P < 0.001, and ****P < 0.0001, ns, not significant).

Of note, β-*O*-linked *N*-acetylglucosamine (GlcNAc) is a dynamic glycosylation attached to serine and threonine of nucleocytoplasmic and mitochondrial proteins. This modification is dynamically concerted by a pair of enzymes, *O*-GlcNAc transferase (OGT) and *O*-GlcNAcase (OGA) (Figure 1a).^11^ Despite the analytical challenges in understanding *O*-GlcNAc biology,^12^ accumulating evidence suggested that *O*-GlcNAcylation coordinates a myriad of biological activities (e.g., epigenetics, transcription, cellular metabolism) in response to environmental cues,^13−15^ and the dysregulation of *O*-GlcNAcylation has been deeply linked to key CCA hallmarks. For instance, *O*-GlcNAcylation is responsible for controlling the metastatic ability of CCA cells via nuclear translocation of NF-κB and heterogeneous nuclear ribonucleoprotein-K (hnRNP-K).^16,17^ Therefore, understanding the nuanced impact of *O*-GlcNAcylation associated with cholangiocarcinoma yields insight into its regulation of cancer-relevant proteins, unveils novel therapeutic targets, and provides scientific paradigm for effective strategies in CCA therapeutics.

The application of mass spectrometry (MS) for intact glycopeptide profiling has surfaced as a potent technique for glycoproteomic analysis.^18,19^ However, annotating the *O*-GlcNAcylation site is challenging due to its biosynthetic complexity, the lack of peptide consensus sequence, and the often-overlapping interplay in the secretory pathway, far hindering the deep elucidation of *O*-GlcNAcylation with molecular details in CCA. Regrettably, we still lack systematic information on the precise glycan attachment site(s) of *O*-GlcNAcylation and the knowledge of their impact(s) on CCA progression.

**Figure 2:**
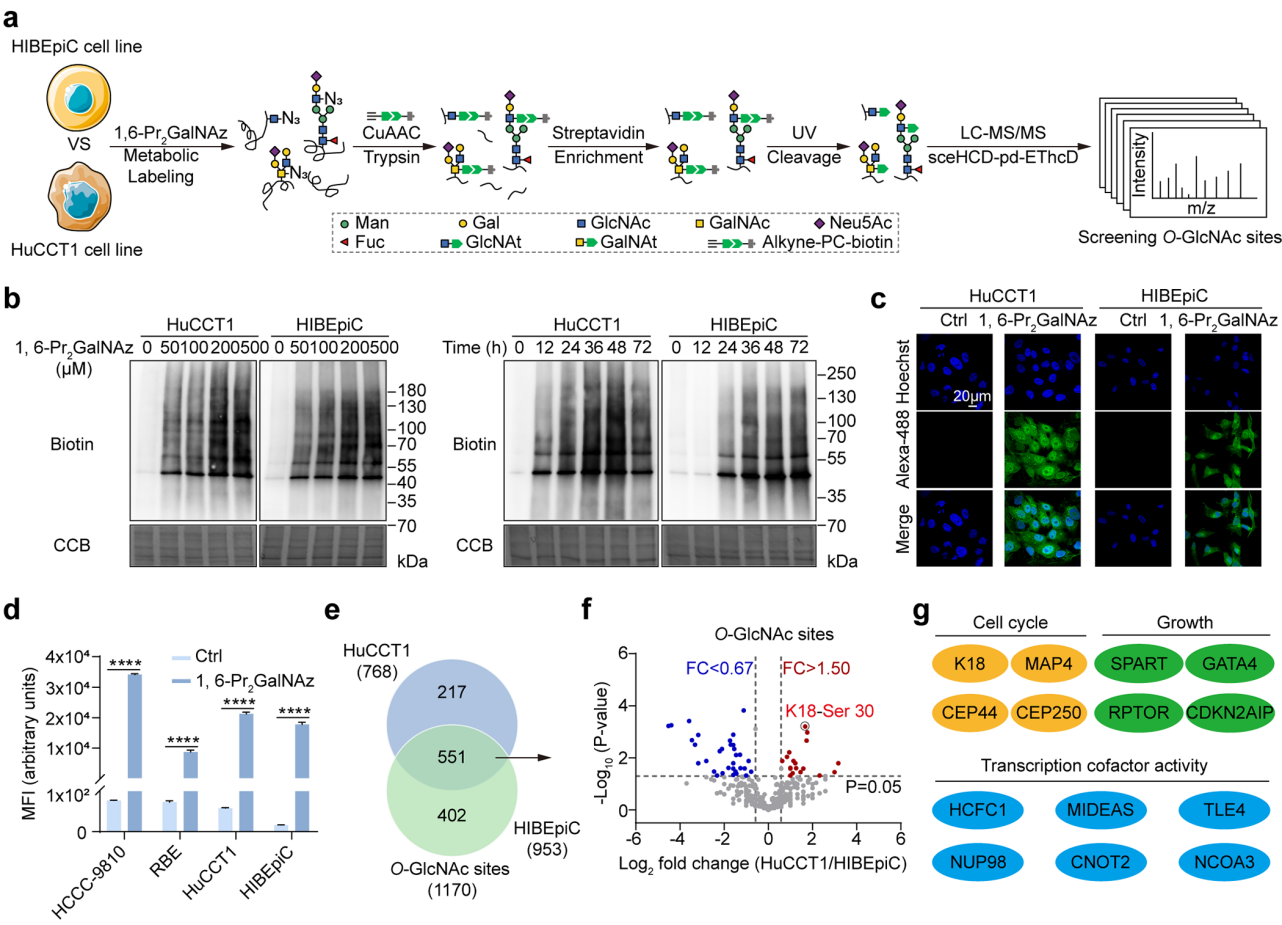
Profiling of protein *O*-GlcNAcylation in CCA by Click-iG. (a) Schematic of the Click-iG strategy in CCA analysis. HuCCT1 and HiBEpiC cells are metabolically incorporated with 1,6-di-*O*-propionyl-*N*-azidoacetylgalactosamine (1,6-Pr_2_GalNAz), reacted with alkyne-PC-biotin via click chemistry, digested and enriched by streptavidin beads, followed by photocleavage release of glycopeptides for whole glycopeptide analysis. (b) Western blot analysis of HuCCT1 and HiBEpiC cells treated with 1,6-Pr_2_GalNAz at various concentrations for different times. The cell lysates were reacted with alkyne-biotin via copper(I)-catalyzed azide–alkyne cycloaddition (CuAAC) and blotted using antibiotin. Equal loading was confirmed using Coomassie brilliant blue staining (CBB). (c) Confocal fluorescence imaging of HuCCT1 and HIBEpiC cells treated with 1,6-Pr_2_GalNAz at 0 or 200 μM for 48 h. The cells were washed, labeled with alkyne-AZDye-488, and analyzed. Scale bar: 20 μm. (d) Flow cytometry analysis of three CCA cells and HIBEpiC cells treated with 1,6-Pr_2_GalNAz at 0 or 200 μM for 48 h. The cells were washed, reacted with alkyne-biotin and Alexa Flour 488-streptavidin, and analyzed. (e) Total numbers of *O*-GlcNAc sites identified in HuCCT1 and HIBEpiC cells in three independent experiments. (f) Volcano plots showing the average log_2_ fold change (HuCCT1/HIBEpiC) for *O*-GlcNAc sites quantified in three independent replicates and *P*-values. *O*-GlcNAc sites with *P*-value < 0.05 and a fold change > 1.50 (red) or < 0.67 (blue) were considered as up-regulated or down-regulated *O*-GlcNAc sites, respectively. (g) *O*-GlcNAcylated proteins involved in the cell cycle, growth, and transcription cofactor activity. sceHCD-pd-EThcD, stepped collision energy based higher-energy collisional dissociation followed by product-dependent electron transfer/higher-energy dissociation; Man, mannose; Gal, galactose; Glu, glucose; GlcNAc, *N*-acetylglucosamine; GalNAc, *N*-acetylgalactosamine; Neu5Ac, *N*-acetylneuraminic acid; Fuc, fucose; GlcNAt, *N*-(4-aminomethyl)-triazolylacetylglucosamine; GalNAt, *N*-(4-aminomethyl)-triazolylacetylgalactosamine; alkyne-PC-biotin, alkyne-photocleavable linker-biotin tag. Data were shown as the mean ± SD; statistical significance was determined by Student’s *t* tests (two-tailed, ****P < 0.0001).

Chemical tools have proven themselves as indispensable instruments for glycobiology research. For instance, small molecule inhibitor Ac_4_5SGlcNAc (designated as 5S thereof) is a known inhibitor of OGT that acts as a metabolic precursor to form uridine diphosphate activated precursor-5SGlcNAc (UDP-5SGlcNAc), and OGA can be selectively and effectively inhibited by Thiamet-G (designated as TMG thereof) (Figure 1a).^20,21^ In addition, our collaborators recently reported the Click-iG strategy, which amalgamates the metabolic oligosaccharide engineering (MOE) of selected, clickable unnatural sugar probes for *O*-GlcNAcylated protein enrichment, and a customized pGlyco3 search engine for intact glycopeptide annotation (Figure 2a).^22,23^ Click-iG outperforms pioneering workflows like IsoTag strategy^24^ and software iterations such as MetaMorpheus O-Pair,^25^ MSFragger-Glyco,^26^ and StrucGP.^27^ It allows for simultaneous and comprehensive profiling of multiple protein glycosylation types at the intact glycosite level in a single experiment, offering enhanced coverage of the protein glycosylation landscape. Leveraging our chemical toolkit at hand, we sought to delineate the molecular details of *O*-GlcNAc modification and its impact on CCA. Here we first systematically perceive the *O*-GlcNAcylation, OGT, and OGA levels in human CCA samples, then perturb the CCA cell lines with Click-iG to profile *O*-GlcNAcome with glycan composition and glycosylation site resolution. We identified that *O*-GlcNAcylated type I cytokeratin, Keratin 18 (K18), can coordinate the tricarboxylic acid (TCA) cycle enzymes, namely isocitrate dehydrogenases (IDHs), to promote CCA progression. Mechanistically, we provide evidence that site-specific *O*-GlcNAcylation of K18 on Ser 30 stabilizes K18, which benefits the expression of cell cycle checkpoints to enhance cell cycle progression and cell growth *in vitro* and *in vivo*. We also demonstrate that *O*-GlcNAcylation on K18 affects and choreographs the TCA cycle, which regulates the level of metabolites, and exhibits stronger resistance toward oxidative stress. Our study thus expands the current understanding of protein *O*-GlcNAcylation and adds another dimension of complexity to post-translational control over metabolism and tumorigenesis.

## Results and Discussion

### Up-regulation of *O*-GlcNAcylation in CCA Is Associated with Its Contribution to Global Cell Proliferation in CCA Cells

We started by investigating the clinical relevance of *O*-GlcNAcylation in CCA. We analyzed the *O*-GlcNAc, and the expression of OGT and OGA in 21 pairs of human resected tumor tissues and adjacent normal bile ducts using immunohistochemistry (IHC) (Figure 1b, Figure S1). The levels of *O*-GlcNAc and OGT were distinctively elevated in tumor tissues compared with normal tissues, consistent with the IHC staining scores in these patients (*P* < 0.001, Figure 1b). However, the level of OGA did not show distinctive changes between the two groups (Figure 1b, Figure S1). We then blotted the *O*-GlcNAc expression levels of peritumoral/tumor tissue pairs from another 15 CCA patients. Again, *O*-GlcNAcylation and OGT were identified to be up-regulated in tumor tissues (Figure S2), and semiquantitative densitometric analysis confirmed that the increases are statistically significant (*P* < 0.01 and 0.05, respectively, by Student’s *t* tests, two-tailed) (Figure 1c). In parallel, we found that the mRNA expression of *OGT* and *OGA* was escalated from the Cancer Genome Atlas (TCGA) and Gene Expression Omnibus (GEO) data sets (GSE32879, GSE107943, GSE119336, GSE76297) (Figure S3a,b). CCA patients with high levels of *OGT* and low levels of *OGA* gene expression also displayed worse overall survival (OS), according to the Kaplan–Meier survival analysis, indicating the pivotal role of *O*-GlcNAcylation in CCA (Figure S3c,d). We further assessed the *O*-GlcNAc, OGT, and OGA expressions in three human CCA cell lines, namely HCCC-9810, RBE, and HuCCT1, and the Human Intrahepatic Biliary Epithelial Cell (HIBEpiC) control cell line (Figure 1d). *O*-GlcNAcylation level increased in all CCA cells compared with HIBEpiC cells, while mRNA expression of *OGT* and *OGA*, as measured by quantitative real-time PCR (qRT-PCR), exhibited varied expression levels (Figure S4). The collective data conclusively identified the increased occurrence of *O*-GlcNAcylation as a shared occurrence in CCA samples.

We next postulated that modulation of *O*-GlcNAc levels would alter CCA oncology phenotypes (i.e., proliferation, apoptosis, cell cycle) by targeting pathways known to regulate CCA progression. To do so, we first used chemical tools 5S and TMG, to block the activity of OGT and OGA, respectively (Figure 1a).^20,21^ Treatment of CCA cell lines with 5S or TMG resulted in robust reduction or elevation of *O*-GlcNAc modification level on proteins, in a time- and dose-dependent manner (Figure S5), consistent with earlier findings.^20,21^ Concurrently, cellular viability was evaluated through the cell counting kit-8 (CCK-8). The predominant cytotoxic effect observed in these cells was attributed to OGT inhibition rather than OGA, indicating that reducing *O*-GlcNAcylation leads to CCA cell death (Figure 1e, Figure S6). Moreover, suppression of OGT promoted HuCCT1 cell apoptosis, as evidenced by the increased percentage of both early apoptosis (FITC^+^/PE^–^) and late apoptosis (FITC^+^/PE^+^) after OGT silencing upon 5S incubation (Figure 1f, Figure S7). However, no remarkable effects of TMG as an antiapoptotic inhibitor were detected (Figure 1f, Figure S7). Similar effects were also observed in RBE cells (Figure S8).

Mounting reports implied that *O*-GlcNAc plays a multifaceted role during the cell cycle, and incongruousness arises partly due to different physiological cues.^28^ We therefore examined the cell cycle distribution of HuCCT1 and RBE cells using flow cytometric analysis after incubation with 5S or TMG at varied concentrations (Figure 1g, Figure S9). Interestingly, cell cycle distribution of HuCCT1 cells was majorly found in the G2/M phase, while RBE cells were arrested in the S phase when treated with 5S but not under the TMG-treated scenarios (Figure 1g, Figure S9). Hence, it can be concluded that maintaining an optimal *O*-GlcNAc level is crucial for all cell cycle phases. Additionally, we probed the classical biomarkers associated with cell cycle and apoptosis after disruption of *O*-GlcNAcylation using 5S and TMG (Figure 1h, Figure S10). While the TMG-treated group displayed no significant variations in these biomarkers, the 5S-treated group exhibited widespread up-regulation in apoptosis markers and down-regulation in antiapoptotic marker and cell cycle checkpoint indicators (Figure 1h, Figure S10). To further explore the link between *O*-GlcNAcylation and CCA progression, we validated three independent small interfering RNAs (siRNAs) for *OGT* or *OGA* knockdown in HuCCT1 and RBE cells, all demonstrating potent knockdown efficacy while maintaining catalytic activities (Figure S11a,b). Clonogenic assays confirmed that *OGT* knockdown in both CCA cell lines significantly reduced cell proliferation and presented an antitumor effect (Figure S11c–f). These biological characteristics synergistically align with chemical tools (Figure S12), underscoring the potential role of global *O*-GlcNAcylation in fostering the development and progression of cholangiocarcinoma (Figure 1i).

### Chemical Enrichment and Profiling of Intact *O*-GlcNAcylated Glycopeptides in CCA

Given that disrupted *O*-GlcNAcylation influences the course of cancer progression in CCA, our subsequent investigation aimed to adopt Click-iG (Figure 2a), a chemical glycoproteomic platform, to systematically enrich, identify, and profile intact *O*-GlcNAcylated glycopeptides with matched information on glycosylation sites and glycan composition.^22,23^ In contrast to established techniques, Click-iG provides extensive coverage of the protein glycosylation landscape, laying a foundation for investigating the interplay between various glycosylation pathways. In brief, we utilized 1,6-di-*O*-propionyl-*N*-azidoacetylgalactosamine (1,6-Pr_2_GalNAz), an optimized *O*-GlcNAc chemical reporter with minimal nonspecific modification and cytotoxicity (Figure S13),^29^ for metabolic incorporation into various glycoconjugates in HuCCT1 or HIBEpiC cells. 1,6-Pr_2_GalNAz can cross cell membranes and undergo deacetylation by nonspecific esterases to generate cell-active GalNAz, which can be metabolically converted to UDP-GalNAz with high efficiency in cells via the GalNAc salvage pathway.^30^ The nicotinamide adenine dinucleotide (NAD)-dependent epimerase UDP-galactose-4-epimerase (GALE) next converts UDP-GalNAz to UDP-GlcNAz that serves as the substrate for *O*-GlcNAcylation by the *O*-β-GlcNAc transferase (OGT). The azido-containing *O*-GlcNAcylated were then reacted with a three-module alkyne-photocleavable linker-biotin tag (alkyne-PC-biotin), followed by trypsin digestion and enrichment using streptavidin beads. After photocleavage with 365 nm ultraviolet (UV), the released, click-labeled glycopeptides were analyzed using liquid chromatography-tandem mass spectrometry (LC-MS/MS) with preferred glycopeptide fragmentation strategies, including stepped collision energy-based higher-energy collisional dissociation (sceHCD) followed by product-dependent electron transfer/higher-energy dissociation (sceHCD-pd-EThcD) (Figure 2a).

Initially, we assessed the metabolic efficacy of 1,6-Pr_2_GalNAz in the CCA and HIBEpiC cell lines and observed dose- and time-dependent azidosugars incorporation (Figure 2b, Figure S14a). Notably, significant fluorescence labeling occurred in nucleocytoplasmic regions when permeabilized 1,6-Pr_2_GalNAz-treated cells were reacted with alkyne-AZDye-488 via the ligand-assisted copper(I)-catalyzed azide–alkyne cycloaddition (CuAAC), in alignment with expected *O*-GlcNAc glycosylation localization (Figure 2c, Figure S14b).^31^ Flow cytometry corroborated these findings across all four cell lines (Figure 2d). Given these results, HuCCT1 and HIBEpiC cells incubated with 200 μM 1,6-Pr_2_GalNAz for 48 h were established as the standardized condition for glycoproteomic analysis.

By using the streamlined MS data analysis and annotation procedure (Figure S15), we mapped a total of 1170 *O*-GlcNAc sites on 368 *O*-GlcNAcylated proteins across three replicate experiments employing the Click-iG workflow (Figure 2a,e and Figures S16a and S17a). The Click-iG strategy provided comprehensive pan-scale intact glycopeptide information, including intact glycosites, glycosites, and glycoproteins in both HuCCT1 and HIBEpiC cells (Figure S17b). For instance, in the HuCCT1 cells, 1872 intact glycosites were identified, with 1094 overlapping those identified in HIBEpiC cells, highlighting the cell-type specific feature of protein glycosylation (Figure S17b, left panel). By glycan classification, we identified a total of 3164 intact glycosites, including 1421 intact *N*-linked glycosites and 573 intact mucin-type *O*-linked glycosites (Figure S17c). The sequence visualized using a probabilistic approach around the identified glycosites revealed typical sequon/motif for *O*-GlcNAcylation, mucin-type *O*-linked glycosylation, and *N*-linked glycosylation (Figures S16b and S17d). Remarkably, Click-iG provided in-depth information on glycan type and composition from intact glycosites (Figures S18 and S19). Gene ontology (GO) analysis indicated that the identified *O*-GlcNAcylated proteins were concentrated in the nucleocytoplasmic region (Figure S16c). In addition, proteins with 23 up-regulated (fold change >1.50, *P* < 0.05) and 36 down-regulated (fold change <0.67, *P* < 0.05) *O*-GlcNAc sites in HuCCT1 cells were successfully enriched using Click-iG (Figure 2f, Tables S1 and S2). Many regulators involved in the cell cycle and growth, as well as transcriptional processes, were identified as *O*-GlcNAcylated proteins (Figure 2g, Figure S20). These results collectively demonstrate that Click-iG enables simultaneous and comprehensive profiling of *O*-GlcNAcylation (and other types of glycosylation) in CCA and HIBEpiC cell lines at intact glycosite level. However, cautions are needed in glycoproteomic data interpretation, because the resulting UDP-GalNAz and UDP-GlcNAz are general nucleotide sugar donors and can be readily incorporated into *N*-linked-, mucin-type *O*-linked-, and *O*-GlcNAcylated-glycoproteins. Therefore, we manually annotate the *O*-GlcNAcylation site with the aid of glycoprotein subcellular location and the presence of *N*-(4-aminomethyl)-triazolylacetylglucosamine (GlcNAt).^22^

### Keratin 18 is Mainly *O*-GlcNAcylated at Ser 30

We noticed that Keratin 18 (K18) is on the list of up-regulated *O*-GlcNAcylated proteins in HuCCT1 cells compared to HIBEpiC control cells (Figure 2f, Tables S1 and S2). Keratins are a family of fibrous structural proteins that play a pivotal role in differentiation and tissue specialization and are highly regulated among various epithelia in a cell-specific manner.^32^ Keratin 18, in conjunction with its heteropolymeric partner, Keratin 8 (K8), forms intermediate filaments and serves as a crucial component of the cytoskeleton.^33^ Keratin 18 is also a versatile protein with functional significance in intracellular scaffolding and cellular processes, and its association with the malignant phenotype in digestive epithelia is well-established.^34^ Previous studies have reported post-translational modification (PTM) on K18, encompassing sumoylation,^35^ acetylation/methylation,^36^*O*-GlcNAcylation,^37^ and reciprocal cross-talk with phosphorylation.^38^

**Figure 3:**
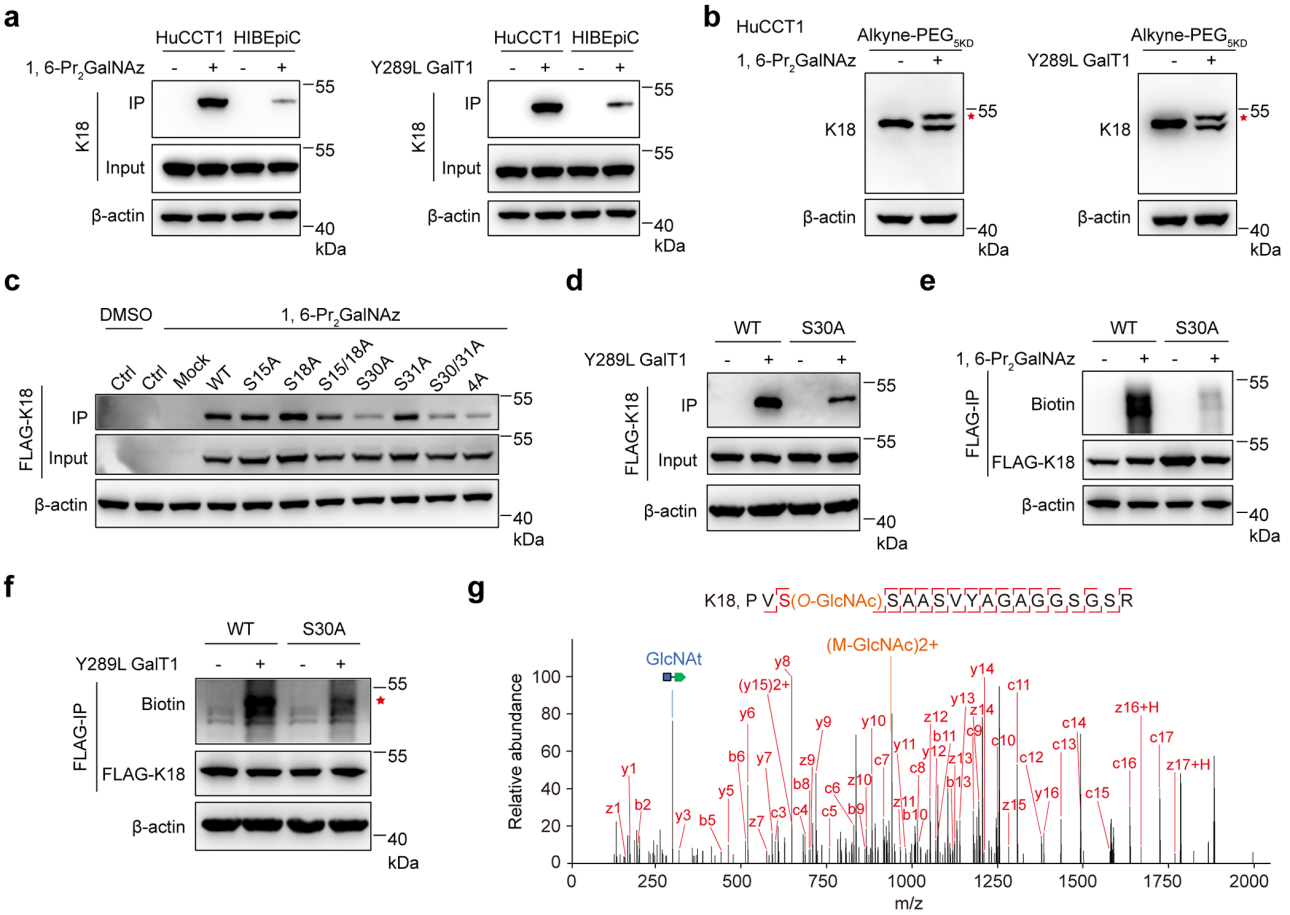
Keratin 18 is mainly *O*-GlcNAcylated at Ser 30. (a) Western blot analysis showing the Keratin 18 (K18) *O*-GlcNAcylation in HuCCT1 and HIBEpiC cells. The cells were incubated with 1,6-Pr_2_GalNAz, lysed, reacted with alkyne-biotin, and captured by streptavidin beads (left) or incubated with Y289L GalT1 and UDP-GalNAz in cell lysates, reacted with alkyne-biotin, and captured by streptavidin beads (right). (b) Western blot analysis showing the *O*-GlcNAcylation stoichiometry of K18 in HuCCT1 cells. The cells were incubated with 1,6-Pr_2_GalNAz, lysed, reacted with alkyne-PEG_5KD_ (left) or incubated with Y289L GalT1 and UDP-GalNAz in cell lysates and reacted with alkyne-PEG_5KD_ (right). The red asterisk indicated tagged *O*-GlcNAcylated K18. (c) Immunoblot analysis of K18 *O*-GlcNAcylation showing HEK293T cell lysates transfected with FLAG-tagged K18 (FLAG-K18) with wild-type, single, double, or quadruple mutations incubated with 1,6-Pr_2_GalNAz, lysed, and immunoprecipitated with streptavidin beads. (d) Immunoblot analysis showing HEK293T cells overexpressing FLAG-K18^WT^ or FLAG-K18^S30A^ incubated with Y289L GalT1 and UDP-GalNAz and immunoprecipitated with streptavidin beads. (e) Immunoblot analysis showing the HEK293T cells overexpressing FLAG-K18^WT^ or FLAG-K18^S30A^ incubated with 1,6-Pr_2_GalNAz, lysed, reacted with alkyne-biotin, immunoprecipitated with anti-FLAG beads, and blotted with antibiotin. (f) Immunoblot analysis showing the HEK293T cells overexpressing FLAG-K18^WT^ or FLAG-K18^S30A^ incubated with Y289L GalT1 and UDP-GalNAz, immunoprecipitated with anti-FLAG beads, and blotted with antibiotin. The red asterisk indicated tagged *O*-GlcNAcylated K18. (g) Representative MS2 spectrum of an *O*-GlcNAcylated peptide from K18 located on Ser 30. The matched fragment ions (red), diagnostic fragment ion (orange), and the GlcNAt fragment ion (blue) are labeled. Equal loadings were confirmed using β-actin in all Western blot analyses. IP, immunoprecipitation; UDP-GalNAz, UDP-*N*-azidoacetylglucosamine; Y289L GalT1, β-1,4-galactosyltransferase mutant; alkyne-PEG_5KD_, alkynylated polyethylene glycol 5000.

To confirm the *O*-GlcNAcylation of K18, HuCCT1 and HIBEpiC cells were incubated with 1,6-Pr_2_GalNAz for 48 h, followed by CuAAC reaction with alkyne-biotin and pull-down with streptavidin beads. Immunoblotting with anti-K18 revealed the azidosugars modification of K18 (Figure 3a, left panel). To further validate the glycan type, we treated both cell lysates with a permissive β-1,4-galactosyltransferase mutant (Y289L GalT1), which transfers *N*-azidoacetylgalactosamine (GalNAz) from its uridine diphosphate activated precursor (UDP-GalNAz) to *O*-GlcNAc residues.^39^ Subsequent chemoselective click reaction with alkyne-biotin and streptavidin enrichment confirmed *O*-GlcNAcylation on endogenous K18 (Figure 3a, right panel). The immunoprecipitated K18 can be directly detected by *O*-GlcNAc-specific antibody RL2, and we observed K18 *O*-GlcNAcylation changes in response to *OGT* knockdown but not to *OGA* (Figure S21). The semiquantitative measurement of *O*-GlcNAc modification on K18 in both HuCCT1 and HiBEpiC cells involved labeling azides with alkynylated polyethylene glycol 5000 (alkyne-PEG5_KD_) in a mass shift assay. The stoichiometric ratio for *O*-GlcNAcylation was around 50% in HuCCT1 cells, contrasting with approximately 20% in HiBEpiC cells (Figure 3b, Figure S22a,b).

Based on Click-iG, we mapped four *O*-GlcNAc modification sites (Ser 15, Ser 18, Ser 30, and Ser 31) on K18, with Ser 30 and Ser 31 being previously reported (Figure S23a).^37^ Surprisingly, although *O*-GlcNAcylation at Ser 49 had been documented before, it was not detected in our experimental conditions.^37^ Significantly, all four identified sites exhibited strong evolutionary conservation across *Homo sapiens*, *Mus musculus*, and *Rattus norvegicus* (Figure S23b). To gauge the relative abundance of *O*-GlcNAc modification on these sites, FLAG-tagged K18 mutants, K18^S15A^, K18^S18A^, K18^S15/18A^, K18^S30A^, K18^S31A^, K18^S30/31A^, and K18^S15/18/30/31A^ (K18^4A^), were transfected into HEK293T cells. Azides introduced either via MOE with 1,6-Pr_2_GalNAz or Y289L GalT1 chemoenzymatic labeling were subjected to biotinylation for streptavidin capture and subsequent immunoblot analysis. Ser 30 emerged as the primary *O*-GlcNAcylation site in K18 (Figure 3c,d), consistent with our chemical proteomic analysis results (Figure 2f). In contrast, we enriched K18 by pulling down with anti-FLAG beads and measured the *O*-GlcNAc levels for FLAG-K18^WT^ or FLAG-K18^S30A^, using biotinylated signals introduced via the above-mentioned glyco-analytical methods. A significant loss of the *O*-GlcNAcylation signal was evident for FLAG-K18^S30A^ compared to FLAG-K18^WT^ (Figure 3e,f). In addition, sceHCD-pd-EThcD-based LC–MS/MS analysis annotated the K18 peptide with amino acid 28–45 (PVSSAASVYAGAGGSGSR) as an *O*-GlcNAcylated peptide at Ser 30 (Figure 3g). The MS2 spectrum of the *O*-GlcNAcylated peptide from K18 located on Ser 15, Ser 18, and Ser 31 was also annotated (Figure S24).

### *O*-GlcNAcylation of K18 Promotes CCA Proliferation and Progression *In Vitro* and *In Vivo*

**Figure 4:**
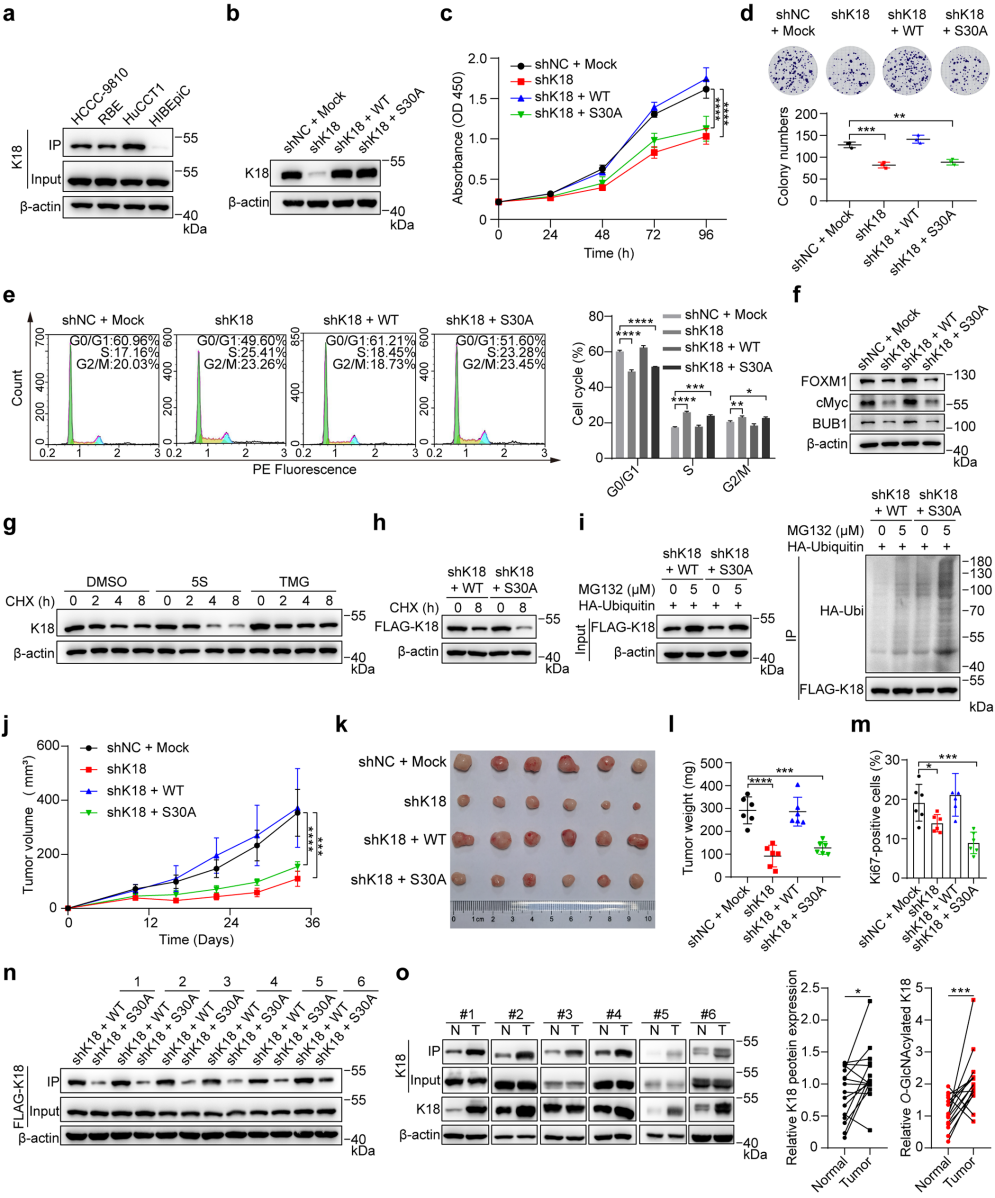
*O*-GlcNAcylation of K18 promotes CCA cell growth *in vitro* and *in vivo*. (a) Western blot analysis of K18 *O*-GlcNAcylation in three CCA cells and HIBEpiC cells. The cell lysates were incubated with Y289L GalT1 and UDP-GalNAz, reacted with alkyne-biotin, and immunoprecipitated by streptavidin beads. (b) Western blot analysis of K18 in HuCCT1 stable cell lines with small hairpin RNA K18 knockdown (shK18) and re-expression of shK18-resistant FLAG-K18 wild-type (shK18 + WT) or FLAG-K18 S30A (shK18 + S30A). Random small hairpin RNA with an empty vector (shNC + Mock) was used as a negative control. (c) Cell counting kit-8 (CCK-8) analysis of HuCCT1 stable cell lines. Absorbance was measured for cell viability. (d) Clonogenic assay of cell proliferation in HuCCT1 stable cell lines. Colony numbers were quantitatively analyzed at the bottom. (e) Cell cycle distribution assays of HuCCT1 stable cell lines. Histogram plot in flow cytometry indicated the percentage of cell populations in the G0/G1, S, or G2/M phase. Quantitative analysis was shown in the right panel. (f) Cell cycle marker analysis of HuCCT1 stable cell lines by Western blot. Protein levels of FOXM1, cMyc (G1/S transition markers), and BUB1(G2/M transition marker) were analyzed. (g) Degradation analysis of K18 in HuCCT1 cells by Western blot. The cells were incubated with DMSO (vehicle), 200 μM 5S, or 1 μM TMG for 48 h, followed by treatment with 10 μg/mL cycloheximide (CHX) for up to 8 h. (h) Degradation analysis of K18 in HuCCT1 shK18 + WT or shK18 + S30A stable cell lines by Western blot. The cells were incubated with DMSO (vehicle) or 10 *μ*g/mL CHX for 8 h. (i) Ubiquitination analysis of K18 in HuCCT1 shK18 + WT or shK18 + S30A stable cell lines. The cells were transfected with HA-ubiquitin, incubated with 5 μM MG-132 (proteasome inhibitor) for 20 h, lysed, and captured with anti-FLAG beads (left). Anti-HA blot demonstrated the ubiquitination of immunoprecipitated FLAG-K18 (right). (j–n) A tumorigenesis assay was performed by subcutaneous injection of HuCCT1 cells with shNC + Mock, shK18, shK18 + WT, and shK18 + S30A into the right flanks of nude mice (*n* = 6). Tumors generated by xenograft HuCCT1 stable cells were measured every 6 days from day 10 (j). After 34 days, tumors were dissected, photographed (k), and weighed (l). Ki67 (a marker of cell proliferation) positive area analysis of xenograft tumors through immunohistochemistry (m). Western blot analysis of K18 *O*-GlcNAcylation in xenograft tumors generated from stable HuCCT1 cell lines (n). (o) Representative images and densitometric analysis of K18 and its *O*-GlcNAcylation levels from 15 pairs of CCA tumor tissues (T) and adjacent normal tissues (N) by Western blot analysis. Equal loadings were confirmed using β-actin in all Western blot analyses. Data were shown as the mean ± SD; statistical significance was determined by Student’s *t* tests (two-tailed, *P < 0.05, **P < 0.01, ***P < 0.001, and ****P < 0.0001).

With the detailed K18 glycosylation information at hand, we next asked whether K18 *O*-GlcNAc modification would impact cholangiocarcinoma phenotype(s). We first assessed the impact of *O*-GlcNAcylation on K18 filament organization in CCA cells. FLAG-K18^WT^ RBE cells displayed higher filament density around the nucleus and gradually decreased toward the cell periphery. In contrast, FLAG-K18^S30A^ counterparts showed increased filament accumulation around the nucleus, with collapsed peripheral filaments. Addition of the OGA inhibitor TMG minimally rescued FLAG-K18^S30A^ filament organization but further enhanced filament organization in FLAG-K18^WT^, consistent with prior studies (Figure S25).^38^ We then examined the K18 *O*-GlcNAcylation levels in typical human CCA cell lines and the normal HIBEpiC cell line. Elevated K18 *O*-GlcNAcylation was observed in all three CCA cell lines, in contrast with the absence of such glycosylation in HIBEpiC cells (Figure 4a). To scrutinize the importance of Ser 30 *O*-GlcNAcylation, we generated stable CCA cell lines with three independent targeting-resistant short hairpin RNA (shRNA) for K18 knockdown (Figures S26a,b, S27a, and S28a) and then restored K18 expression using either FLAG-K18-WT or FLAG-K18-S30A (Figure 4b, Figures S26c, S27b, and S28b). Systematic evaluation of both *KRT18* mRNA expression and knockdown efficiency identified shK18-2 as the optimal construct (designated as shK18 thereof). Cell proliferation was severely inhibited in HuCCT1 cells upon K18 depletion, as evidenced by CCK-8 and colony formation assays. This inhibitory effect was rescued by the re-expression of FLAG-K18-WT but not FLAG-K18-S30A (Figure 4c,d). Similar results were also observed in RBE and HCCC-9810 cells (Figures S27c,d and S28c,d). These data imply that Ser 30 *O*-GlcNAcylation is functionally crucial in CCA proliferation *in vitro*. We next examined the cell cycle distribution in each rescued cell line and found that FLAG-K18-S30A-rescued HuCCT1, RBE, and HCCC-9810 cells exhibited an elevation in S and G2/M phase arrest. This finding is partially in conformity with a previous report indicating that *O*-GlcNAc on K18 is increased during G2/M phase arrest (Figure 4e, Figures S27e and S28e).^40^ Moreover, immunoblot analysis of cell cycle biomarkers in these rescued cell lines displayed similar correlations (Figure 4f, Figures S27f and S28f).

Considering the reported impact of *O*-GlcNAcylation on K18 in regulating its solubility, filament organization, and stability,^41^ we further investigated whether *O*-GlcNAcylation at Ser 30 modulates K18 stability. Previous studies demonstrated that the half-life for K18 is regulated by *O*-GlcNAcylation in the human hepatocytes (Chang) cell line.^38,41^ To assess stability, CCA cell lines were treated with cycloheximide (CHX, a protein synthesis inhibitor) for an immunoblot chase assay. OGT inhibition by 5S considerably accelerated K18 degradation, while silencing OGA with TMG had minimal effect on its decay rate (Figure 4g, Figures S27g and S28g). Similarly, FLAG-K18-WT exhibited greater stability than FLAG-K18-S30A after 8 h of protein lifespan (Figure 4h, Figures S27h and S28h). Correlatively, an increased level of ubiquitination was observed in FLAG-K18-S30A (Figure 4i, Figures S27i and 28i). These findings suggest that the enhanced stability of K18 is associated with the up-regulation of *O*-GlcNAcylation at Ser 30 and the concurrent inhibition of ubiquitination.

To decipher the impact of K18 *O*-GlcNAcylation on tumor growth *in vivo*, we injected BALB/c nude mice with stable HuCCT1 cell lines, including shRNA negative control with an empty vector (shNC + Mock), shK18, shK18+WT, shK18+S30A, and tumor formation was quantitatively measured in these groups. K18 depletion and its mutant at Ser 30 greatly repressed tumor growth rate, tumor size/weight, and the Ki67 positive percentage (a marker to determine cancer cell proliferation) (Figure 4j–m, Figure S29). Conversely, tumor tissues dissected from HuCCT1 cells expressing FLAG-K18-WT exhibited a higher level of K18 *O*-GlcNAcylation compared to cells expressing FLAG-K18-S30A (Figure 4n). Encouragingly, we observed that both protein expression and *O*-GlcNAcylation levels of K18 were markedly elevated in clinical CCA tumor tissues compared to adjacent normal tissues (Figure 4o, Figure S30). These findings mirror most of the observed *in vitro* effects and support the hypothesis that *O*-GlcNAcylation of K18 promotes CCA progression *in vivo*.

### K18 *O*-GlcNAcylation Promotes K18-Isocitrate Dehydrogenase Interaction to Regulate the TCA Cycle in CCA

**Figure 5:**
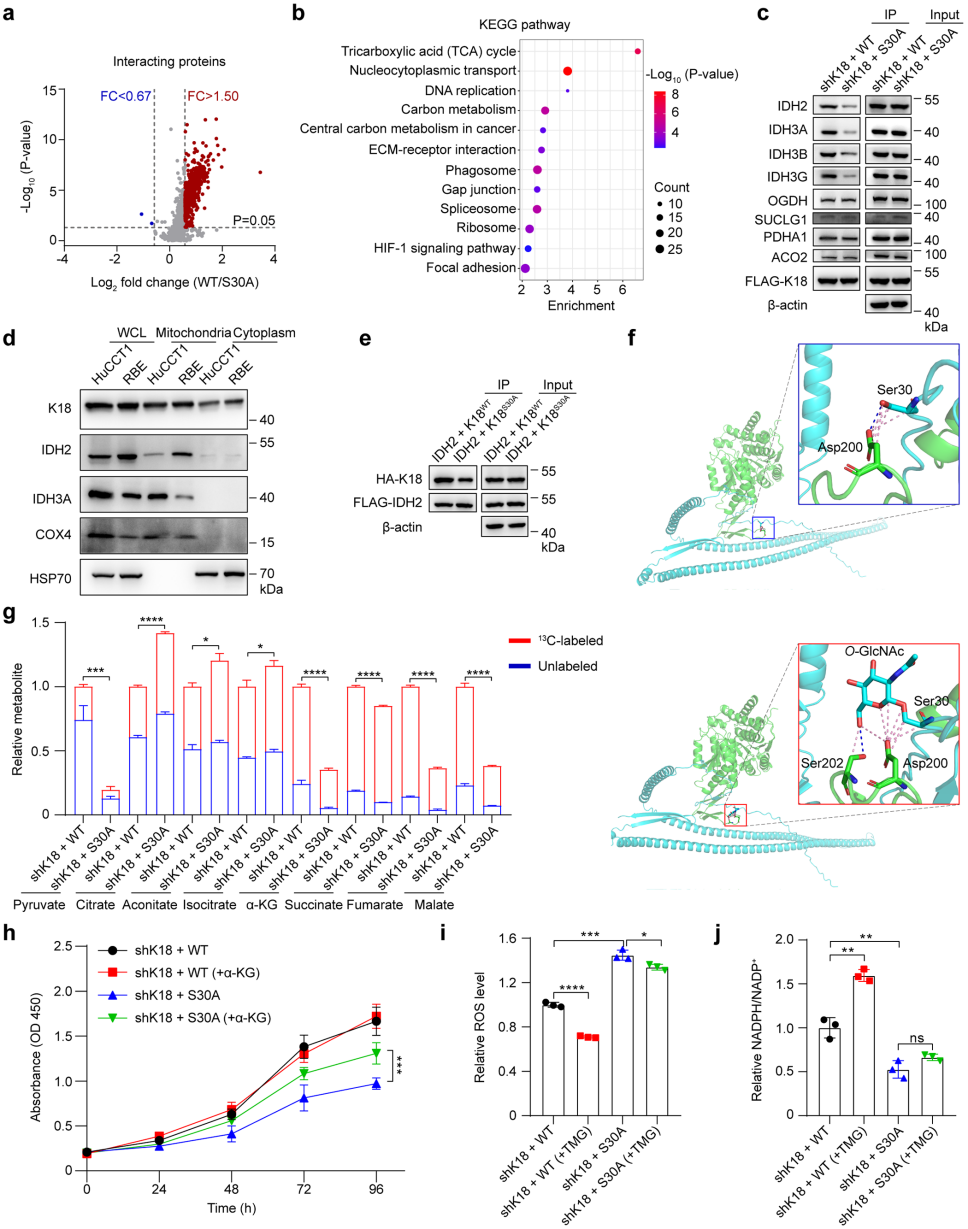
*O*-GlcNAcylation promotes K18-isocitrate dehydrogenase interaction to regulate the TCA cycle in CCA. (a) Volcano plot showing the relative abundance of interacting proteins with K18 in HuCCT1 shK18 stable cells transfected with FLAG-K18^WT^ or FLAG-K18^S30A^, the average log_2_ fold change for proteins quantified in three independent replicates and *P*-values. Proteins with *P*-value < 0.05 and a fold change > 1.50 (red) or < 0.67 (blue) were considered as up-regulated or down-regulated interacting proteins, respectively. (b) KEGG enrichment analysis for the upregulated interacting proteins with K18 in HuCCT1 shK18 cells transfected with FLAG-K18^WT^ compared with FLAG-K18^S30A^. (c) Analysis of enzymes in the TCA cycle interacting with K18 of HuCCT1 stable cell lines (shK18 + WT and shK18 + S30A). Protein levels of IDH2, IDH3A, IDH3B, IDH3G, OGDH, SUCLG1, PDHA1, ACO2, and FLAG-K18 were analyzed by Western blot and immunoprecipitation analysis. Equal loadings were confirmed using β-actin. (d) HuCCT1 and RBE cells were homogenized and subjected to subcellular fractionation, followed by immunoblotting analysis for cellular distribution of K18, IDH2, and IDH3A. COX4 and HSP70 were used as mitochondrial and cytoplasmic markers, respectively. (e) Western blot and immunoprecipitation analysis showing the HA-K18 and FLAG-IDH(s) protein levels in RBE cells cotransfected with FLAG-IDH2 and HA-K18^WT^ or HA-K18^S30A^ followed with lysing and enrichment using anti-FLAG beads. Equal loadings were confirmed using β-actin. (f) Spatial conformation of IDH2-nonglycosylated K18 binding and IDH2-glycosylated K18 (*O*-GlcNAcylated at Ser30) binding. The green and cyan indicated IDH2 and K18, respectively. (g) Relative abundance of metabolites in HuCCT1 stable cell lines (shK18 + WT and shK18 + S30A) labeled with [U–^13^C_6_] glucose for 8 h. (h) CCK-8 analysis of HuCCT1 stable cell lines (shK18 + WT and shK18 + S30A) after treating with or without 5 mM cell-permeable α-KG. Absorbance was measured for cell viability. (i–j) Relative ROS levels (i) and relative NADPH/NADP^+^ ratio (j) of HuCCT1 stable cell lines (shK18 + WT and shK18 + S30A) after treating with or without 1 μM TMG for 48 h. KEGG, Kyoto Encyclopedia of Genes and Genomes; TCA, tricarboxylic acid; and α-KG, α-ketoglutaric acid. Data were shown as the mean ± SD; statistical significance was determined by Student’s *t* tests (two-tailed, *P < 0.05, **P < 0.01, ***P < 0.001, and ****P < 0.0001, ns, not significant).

*O*-GlcNAcylation serves as a nutrient rheostat in a myriad of physiological contexts, especially in metabolically active organs such as the liver.^42−44^ Interestingly, K18 is also abundant in tissues with high rates of cellular turnover, such as the liver, pancreas, and gastrointestinal tract.^34,45^ Given the close relationship between *O*-GlcNAcylation and glucose metabolism, we were motivated to explore the mechanistic insights of how K18 *O*-GlcNAcylation influences its interaction.^46^ We transfected the HuCCT1 shK18 stable cell line with FLAG-K18^WT^ or FLAG-K18^S30A^ with equivalent protein expression levels, coimmunoprecipitated K18-interacting proteins with anti-FLAG beads, and subjected them to LC–MS/MS analysis (Figure S31). We identified 858 up-regulated (fold change >1.50, *P* < 0.05) interacting proteins in FLAG-K18^WT^ HuCCT1 cells compared to FLAG-K18^S30A^ cells (Figure 5a). The majority of these proteins were closely associated with the tricarboxylic acid (TCA) cycle, as revealed by the Kyoto Encyclopedia of Genes and Genomes (KEGG) pathway analysis (Figure 5b). To further explore K18-binding partners within the TCA cycle, we analyzed 8 out of 13 up-regulated TCA enzymes in HuCCT1 cells, by immunoblotting with their respective antibodies (Table S3). In particular, we noticed apparent signal decreases in isocitrate dehydrogenases (IDHs), including IDH2, IDH3A, IDH3B, and IDH3G, when *O*-GlcNAc modification at Ser 30 of K18 was functionally amputated (Figure 5c). Similar results were observed in RBE-rescued cells (Figure S32).

Keratin 18 typically localizes within the cytosol, while IDHs primarily reside in the mitochondrial matrix.^47,48^ To validate the accessibility of K18 to IDHs and eliminate the possibility of postlysis artifacts, we performed subcellular fractionation of mitochondria and cytoplasm. We measured the biodistribution of endogenous K18 (Figure 5d), as well as exogenous HA-tagged wild-type K18 (HA-K18^WT^) and K18 S30A mutant (HA-K18^S30A^) when these constructs were individually cotransfected with FLAG-tagged IDH(s) (Figure S33). Notably, K18 exhibited consistent distribution in both the cytoplasm and mitochondrial matrix, coinciding with previous findings on K8 (Figure S33).^49^ To further confirm the existence of interactions between K18 and IDHs, HA-K18^WT^ and HA-K18^S30A^ were individually cotransfected with FLAG-tagged IDH(s) in RBE cells. After enrichment procedures using anti-FLAG beads, the HA-K18 levels were assessed. We successfully identified K18 in enriched IDH2, IDH3A, IDH3B, and IDH3G samples (Figure 5e, Figure S34a–c). Importantly, the *O*-GlcNAcylation of K18 at Ser 30 facilitated its interaction with IDHs, as evidenced by the increased enrichment level of K18 in HA-K18^WT^ compared to HA-K18^S30A^ (Figure 5e, Figure S34a–c). To explore the atomic-level recognition and binding mechanism between K18 and IDH2, we computationally simulated the interaction between nonglycosylated K18 (AlphaFold Protein Structure Database, ID: AF-P05783-F1) and IDH2 (RCSB PDB ID: 5I96, homology modeling development on SWISS-MODEL server), as well as *O*-GlcNAcylated K18 at Ser30 (modeled with Glycoprotein Builder of GlyCAM) and IDH2, based on their structural information, using molecular docking. Encouragingly, we observed 9 interactions between *O*-GlcNAcylated K18 at Ser30 and IDH2, compared to 7 interactions with its nonglycosylated counterpart in the complex protein (Figure 5f). Detailed examinations of the interface interactions for both complexes revealed, in addition to the universal interaction with Asp200, a distinct hydrogen bond and more nonbonded contacts between the *N*-acetylglucosamine at Ser30 and the Ser202 residue of IDH2. This suggests that IDH2 may be an *O*-GlcNAc-dependent Keratin 18 interactor (Figures S35 and S36). To validate the computational prediction, we generated FLAG-tagged wild-type IDH2 and its Ser202 mutation (FLAG-IDH2^S202A^). We cotransfected these constructs with wild-type K18 (K18^WT^) and/or K18 mutants (K18^S30A^) in RBE cells, a representative cholangiocarcinoma cell line. Western Blot and immunoprecipitation analyses confirmed a decrease in the interaction between IDH2^S202A^ and K18^WT^ compared to IDH2^WT^+K18^WT^. Additionally, there was no significant change in the interaction between IDH2^S202A^ and K18^S30A^ compared to IDH2^WT^+K18^S30A^ (Figure S37). These findings suggest a potential mechanism where *O*-GlcNAcylation may contribute an additional hydrogen bond, stabilizing the interaction between K18 and IDH2.

In their functional roles, IDHs facilitate the oxidative decarboxylation of isocitrate to α-ketoglutarate and reduce NAD(P)^+^ to NAD(P)H, serving as a pivotal player in aerobic energy production within the TCA cycle.^50,51^ This process includes the oxidation of isocitrate to oxalosuccinate, with NAD(P)H as the electron acceptor, followed by the decarboxylation of oxalosuccinate to produce α-ketoglutarate (α-KG). Subsequently, we aimed to explore whether K18 *O*-GlcNAcylation enhances its interaction with TCA enzymes, thus influencing CCA metabolism and progression. We first assayed the metabolic profile of HuCCT1 and RBE cells by quantifying the ATP production through glycolysis or oxidative phosphorylation (OXPHOS) and found a predominant reliance on glycolysis (Figure S38), consistent with the Warburg effect commonly observed in cancer cells.^52^ Next, we analyzed the metabolic consequences of K18 using stable isotope tracing with [U-^13^C_6_] glucose in shK18+WT and shK18+S30A HuCCT1 stable cell lines. Cellular metabolites involved in the TCA cycle were analyzed using LC–MS/MS analysis. Comparative quantification revealed that, in the absence of K18 *O*-GlcNAcylation at Ser 30, cells exhibited increases in citrate, aconitate, and isocitrate, while witnessing decreases in pyruvate, α-KG, succinate, fumarate, and malate (Figure 5g). The argumentation is further substantiated by the partial recovery of cell proliferation in shK18+S30A cells upon the addition of 5 mM cell-permeable α-KG to the culture medium (Figure 5h). Finally, in evaluating the cellular response to hydrogen peroxide (H_2_O_2_) stress, we observed that the mutation in K18, specifically in the shK18+S30A group, significantly compromised cellular physiological resilience (Figure S39). Additionally, when using TMG to regulate *O*-GlcNAcylation, a global decrease in reactive oxygen species (ROS) was observed, with a more pronounced impact on the shK18+WT group compared to shK18+S30A (Figure 5i). This reduction in ROS levels may be partially attributed to the increase in the relative NADPH/NADP^+^ ratio, which served as key modulators for metabolism reconfiguration (Figure 5j).^53^

## Conclusion

**Figure 6:**
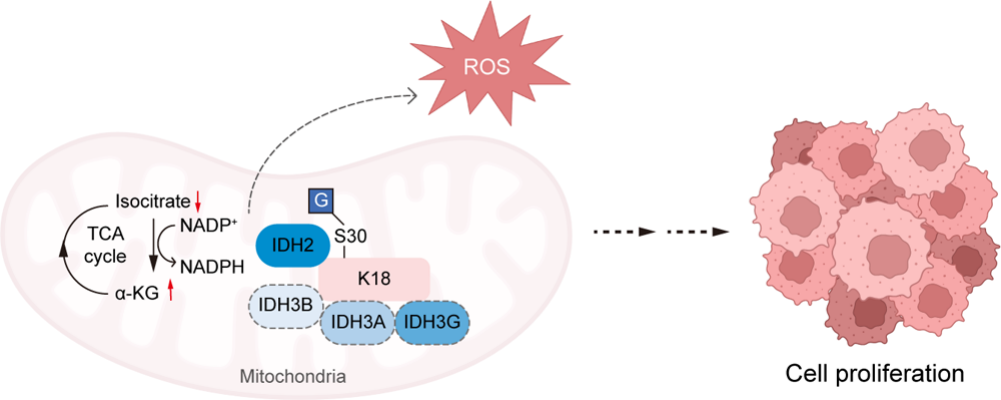
Proposed functional action of K18 *O*-GlcNAcylation in promoting CCA progression.

*In toto*, we systematically asked how knowledge of *O*-GlcNAcylation has led to the aberrant state of cell proliferation and tumorigenesis in cholangiocarcinoma. By exploiting a chemical glycoproteomic strategy Click-iG, our data strongly indicate that *O*-GlcNAcylation on Ser30 of Keratin 18 positively affects CCA cell proliferation and xenograft tumor growth. Chemical proteomic tools enable comprehensive coverage of the protein glycosylation landscape, which provides a blueprint for interrogating the crosstalk between different glycosylation pathways, showcasing the power of such tools in elucidating functional glycobiology. Mechanistically, we provide evidence that enhanced interactions between K18 and isocitrate dehydrogenases, namely IDH2, IDH3A, IDH3B, and IDH3G, orchestrate with the TCA cycle to regulate the level of metabolites in mitochondria. Additionally, *O*-GlcNAcylation on K18 also choreographs with cofactors used in anabolic reactions, to enhance oxidative stress resistance (Figure 6). Interestingly, *IDHs* are also considered as proto-oncogenes in cancer metabolic derangement.^42,54^ These findings add novel mechanistic insights into the regulation of K18 and highlight the potential to intervene in K18 *O*-GlcNAcylation as a therapeutic strategy against CCA tumorigenesis.

The reciprocal interplay between *O*-GlcNAcylation and phosphorylation is also a critical event in modulating protein–protein interaction, subcellular localization, and protein degradation.^55−57^ Competitive site blocking between *O*-GlcNAcylation and phosphorylation (e.g., Ser 49), as well as their synergism (e.g., Ser 31 *O*-GlcNAcylation and Ser 34 phosphorylation), poise functional modulation in K18 solubility, filament organization, and stability.^38,58^ Although beyond the scope of this research, it will be of interest to investigate whether K18 *O*-GlcNAcylation has crosstalk with phosphorylation or other PTM. Nevertheless, cellular metabolism of monosaccharide chemical reporter 1,6-Pr_2_GalNAz inevitably alters the endogenous level of UDP-GlcNAc and UDP-GalNAc, and therefore, it is not ruled out that UDP-GlcNAz or UDP-GalNAz may interfere with the catalytic activity of glycosyltransferases in CCA cell lines. Due to the accessibility of bioorthogonal reaction and the dynamic feature of *O*-GlcNAc modification, it is still challenging to bypass the abundance bias and generate MS spectra for glycosite identification with precise annotation. As *O*-GlcNAcylation occurs on thousands of protein substrates, targeting OGT and OGA using either chemical or biological methodologies might sabotage normal biological processes. In addition, other functional consequences of K18 *O*-GlcNAcylation at other sites still remain ambiguous because only a limited number of tools exist to study its site-specific *O*-GlcNAcylation effects.^59^ Further preclinical and clinical studies are needed to confirm the trueness of CCA-associated *O*-GlcNAcylated proteins as diagnosis/prognosis indicators. The integration of glycomics and other “omics” such as genomics or transcriptomics in CCA cell lines or tissues from patients will provide an avenue for greater impact on developing therapeutics. Additional studies are required to address these questions in the future.

## Materials and Methods

### Patient Samples and Statement

Human CCA tumor tissues and adjacent normal tissues were collected from patients undergoing surgery at the Drum Tower Hospital Affiliated to the Medical School of Nanjing University (Nanjing, China). Written consent was obtained from all patients, and all experiments in this study were conducted in accordance with official guidelines (Declaration of Helsinki), approved by the Medical Ethics Review Committee of Nanjing Drum Tower Hospital (Nanjing, China).

### Cell Lines and Cell Culture

Human CCA cells HCCC-9810 and RBE and HEK293T cells were purchased from the Institute of Biochemistry and Cell Biology, Shanghai Institutes for Biological Sciences, Chinese Academy of Sciences, Shanghai, China. Human CCA cells HuCCT1 were purchased from the Japanese Collection of Research Bioresources Cell Bank (JCRB, Osaka, Japan). HIBEpiC cells were purchased from Shanghai Zhong Qiao Xin Zhou Biotechnology (Shanghai, China). All cell lines were identified using short tandem repeat profiling and tested negative for mycoplasma contamination. HCCC-9810, RBE, and HuCCT1 cells were cultured in RPMI 1640 medium (Gibco, CA, USA). HEK293T cells were cultured in the DMEM medium (Gibco, CA, USA). HIBEpiC cells were cultured in the epithelial cell medium supplementing with 1% epithelial cell growth supplement (EpiCGS), which was purchased from Shanghai Zhong Qiao Xin Zhou Biotechnology (Shanghai, China). All cell lines were cultured with 10% fetal bovine serum (FBS, Gibco, USA) and 100 U/mL of penicillin and streptomycin (Gibco, USA) in a cell incubator at 37 °C with 5% CO_2_. Cells were transfected with siRNAs or plasmid in this study using jetPRIME transfection reagent (Polyplus) when they reached approximately 80% confluence.

### Reagents

Dimethyl sulfoxide (DMSO, cat. no. D2650), CuSO_4_ (cat. no. 931071), sodium ascorbate (cat. no. A7631), methanol (cat. no. 439193), urea (cat. no. U5378), ammonium bicarbonate (ABC, cat. no. A6141), dithiothreitol (DTT, cat. no. D9779), iodoacetamide (IAA, cat. no. I1149), *N*-2-hydroxyethylpiperazine-*N*-2-ethanesulfonic acid (HEPES, cat. no. H4034), MnCl_2_ (cat. no. 429449), chloroform (cat. no. 650498), G418 disulfate salt (cat. no. A1720) and glucose (cat. no. 158968) were purchased from Sigma-Aldrich. Thiamet-G (cat. no. S7213) and puromycin 2HCl (cat. no. S7417) were purchased from Selleck. Alkyne-PC-biotin (cat. no. BB-28) and 2-(4-((bis((1-*tert*-butyl-1H-1,2,3-triazol-4-yl)methyl)amino)methyl)-1H-1,2,3-triazol-1-yl) acetic acid (BTTAA, cat. no. BDJ-4) were purchased from Confluore Biotech (Xi’an, China). The mass spectra grade trypsin (cat. no. HLS TRY001C) was purchased from Beijing Life Proteomic (Beijing, China). Alkyne-biotin (cat. no. 1137) and alkyne-AZDye-488 (cat. no. 1277) were purchased from Click Chemistry Tools. Nonidet P-40 (NP-40, cat. no. 20103ES) was purchased from Yeasen (Shanghai, China). UDP-GalNAz (cat. no. CLK-077) was purchased from Jena Bioscience. Alkyne-PEG_5KD_ (cat. no. HWG26053) and α-ketoglutaric acid sodium salt (cat. no. SM38355-2) were purchased from HWRK CHEM (Beijing, China). Cycloheximide (CHX, cat. no. HY-12320) and MG132 (cat. no. HY-13259) were purchased from MedChemExpress. Oligomycin (cat. no. O399693) and 30% (w/w) H_2_O_2_ solution (cat. no. H112517) were purchased from Aladdin. [U-^13^C_6_] glucose (cat. no. CLM-1396-PK) was purchased from Cambridge Isotope Laboratories. The FLAG-tagged IDH2 (cat. no. F106750), IDH3A (cat. no. F121725), IDH3B (cat. no. F101540) and IDH3G (cat. no. F101620) constructs were purchased from Youbio. Ac_4_5SGlcNAc, 1,6-Pr_2_GalNAz, Y289L GalT1, and HA-Ubiquitin Vector were gifted from Dr. Xing Chen’s lab. Pierce bicinchoninic acid (BCA) Kit (cat. no. 23227) and Hoechst 33342 (cat. no. H3570) were purchased from Thermo Fisher Scientific. FDbio-Dura enhanced chemiluminescence (ECL) Kit (cat. no. FD8020) was purchased from Fdbio science (Hangzhou, China). jetPRIME transfection reagent (cat. no. 101000046) was purchased from Polyplus. RNA isolater Total RNA Extraction Reagent (cat. no. R401-01), HiScript III RT SuperMix for qPCR (+gDNA wiper, cat. no. R323-01), ChamQ Universal SYBR qPCR Master Mix (cat. no. Q711-02), Cell Counting Kit-8 (cat. no. A311-02), and ClonExpress Ultra One Step Cloning Kit (cat. no. C115-02) were purchased from Vazyme (Nanjing, China). BeyoBlue Coomassie Brilliant Blue Ultrafast Staining Solution (CBB, cat. no. P0017F), Annexin V-FITC Cell Apoptosis Detection Kit (cat. no. C1062L), Cell Cycle Detection Kit (cat. no. C1052), Radioimmunoprecipitation Assay (RIPA) lysis buffer (cat. no. P0013K), ATP Quantitation Kit (cat. no. S0027), Cell Mitochondria Isolation Kit (cat. no. C3601), ROS Detection Kit (cat. no. S0033S), NADP^+^/NADPH Quantitation Kit (cat. no. S0179), and NAD^+^/NADH Quantitation Kit (cat. no. S0175) were purchased from Beyotime (Shanghai, China).

### Immunohistochemistry Analysis

The formalin-fixed, paraffin-embedded samples were sliced, dewaxed, and rehydrated, followed by incubation with anti-*O*-GlcNAc (RL2, 1:200, MA1-072, Thermo Fisher Scientific), anti-OGT (1:200, ab177941, Abcam), anti-OGA (1:200, ab124807, Abcam) or anti-Ki67 (1:200, GB111499, Servicebio) antibody, and secondary antibody. After that, samples were incubated with diaminobenzidine (Dako, USA) and restained with hematoxylin (Sigma-Aldrich). The analysis of the IHC score (RL2 (anti-*O*-GlcNAc), OGT, and OGA staining) of CCA tumor tissues and adjacent normal bile duct was performed as previously described.^44^ Level of staining: 0, negative; 1, weakly positive; 2, positive; and 3, strongly positive. For Ki67 staining, the positive area of xenograft tumors was determined through IHC analysis.

### Protein Extraction and Western Blot Analysis

The cells were lysed using 4% sodium dodecyl sulfate (SDS, w/v) supplementing with protease inhibitor (Beyotime) under sonication. After centrifugation, the supernatant was quantified for protein concentration using a BCA kit (Pierce, USA).

For Western blot analysis, cell lysates supplemented with 5× loading buffer were boiled at 99 °C for 5 min. Equal amounts of proteins were separated by SDS–PAGE gel and blotted onto a polyvinylidene fluoride (PVDF) membrane. After blocking with 5% nonfat powdered milk (w/v), the membrane was incubated with specific primary antibodies for anti-*O*-GlcNAc (RL2, 1:2000, ab2739, Abcam), anti-OGT (1:2000, ab177941, Abcam), anti-OGA (1:2000, ab124807, Abcam), anti-β-actin (1:5000, FD0060, Fdbio), anti-Cleaved PARP (1:1000, #5625, Cell Signaling Technology), anti-Cleaved Caspase-3 (1:1000, #9664, Cell Signaling Technology), anti-Bcl2 (1:1000, A19693, ABclonal), anti-FOXM1 (1:1000, sc-376471, Santa Cruz), anti-cMyc (1:1000, AF6513, Beyotime), anti-BUB1 (1:1000, DF6698, Affinity), anti-Cyclin D1 (1:1000, AF0126, Beyotime), anti-Cyclin E1 (1:1000, AF6384, Beyotime), anti-Cyclin A2 (1:1000, AF6624, Beyotime), anti-Cyclin B1 (1:1000, AF6627, Beyotime), anti-K18 (1:1000, sc-6259, Santa Cruz), anti-FLAG (1:2000, M20008, Abmart), anti-FLAG (1:1000, AG8050, Beyotime), anti-Nup98 (1:1000, sc-74553, Santa Cruz), anti-Nup153 (1:1000, sc-101544, Santa Cruz), anti-MIDEAS (1:1000, sc-514710, Santa Cruz), anti-FOXK1 (1:1000, sc-373810, Santa Cruz), anti-CNOT2 (1:1000, sc-81229, Santa Cruz), anti-HA (1:1000, M20003, Abmart), anti-IDH2 (1:1000, sc-374476, Santa Cruz), anti-IDH3A (1:1000, sc-398021, Santa Cruz), anti-IDH3B (1:1000, A13742, ABclonal), anti-IDH3G (1:1000, sc-365489, Santa Cruz), anti-OGDH (1:1000, 15212-1-AP, Proteintech), anti-SUCLG1 (1:1000, 14923-1-AP, Proteintech), anti-PDHA1 (1:1000, 18068-1-AP, Proteintech), anti-ACO2 (1:1000, 11134-1-AP, Proteintech), anti-COX4 (1:1000, 66110-1-Ig, Proteintech), and anti-HSP70 (1:1000, AF1156, Beyotime), followed by secondary horseradish peroxidase (HRP)-conjugated antibodies. After washes with Tris-buffered saline with Tween 20 (TBST), blots were reacted with the ECL reagent (Fdbio science), and protein bands were detected by a chemiluminescence system (Tanon-5200, Shanghai, China).

### qRT-PCR Analysis

Total RNA was isolated from cultured cells by RNA isolater Total RNA Extraction Reagent (Vazyme) according to the manufacturer’s instructions. To quantify mRNAs, total RNA was converted to cDNA using the reverse transcription kit (Vazyme), followed by PCR using ChamQ Universal SYBR qPCR Master Mix (Vazyme) and gene-specific primers (Table S4). All of the reactions were run in triplicate. The expression levels of mRNAs were normalized to *ACTB* mRNA using the 2^–ΔΔC_T_^ method.

### CCK-8 Assay

The cell viability of HuCCT1, RBE, and HCCC-9810 cells was determined using the CCK-8 assays (Vazyme) following the manufacturer’s instructions. Briefly, HuCCT1, RBE, and HCCC-9810 cells were seeded into 96-well plates at a density of 1 × 10^4^ cells/well, followed by exposure to a gradient concentration of 5S and TMG for 48 h. To analyze the effect of H_2_O_2_ on cell proliferation, the cells were seeded into 96-well plates with 2 × 10^4^ cells/well, followed by exposure to a gradient concentration of H_2_O_2_ for 12 h. The cells seeded into 96-well plates with 5 × 10^3^ cells/well were subjected to 5 mM cell-permeable α-ketoglutarate for the indicated time. Finally, 100 μL/well RPMI 1640 medium containing 10% CCK-8 was replaced into the test well and incubated at 37 °C for 1 h. Absorbance was then measured at a wavelength of 450 nm.

### Cell Apoptosis Assay

HuCCT1, RBE, and HCCC-9810 cells were seeded into 6-well plates at a density of 4 × 10^5^ cells/well, followed by exposure to a gradient concentration of 5S and TMG. After incubation for 48 h, the cells were harvested for apoptosis analysis. Briefly, the cells were washed twice with cold PBS and resuspended in 1× binding buffer with 1 × 10^6^ cells/mL followed by the addition of FITC-Annexin V (FITC fluorescence) and PI (PE fluorescence). The cells were incubated at room temperature for 15 min in the dark and were analyzed by flow cytometry (Agilent) within 1 h after staining.

### Cell Cycle Analysis

Cell cycle distribution of HuCCT1, RBE, and HCCC-9810 cells followed by exposure to a gradient concentration of 5S and TMG for 48 h was detected by FACS analysis. Briefly, cells were trypsinized into single-cell suspension, rinsed with ice-cold PBS, and fixed in ice-cold 70% ethanol overnight. Then the cells were stained in PI added with RNase A (100 μg/mL) at 37 °C for 30 min and determined by flow cytometry (Agilent).

### Colony Formation Assay

HuCCT1 and RBE cells transfected with ncRNA, siOGT-1, siOGT-2, siOGT-3, siOGA-1, siOGA-2, or siOGA-3 were seeded into 6-well plates at a density of 300 cells/well and cultured in RPMI 1640 medium supplemented with 10% FBS for 14 days, during which the medium was replaced every 3 days. Colonies were then fixed with methanol for 10 min and stained with 4% crystal violet (Solarbio) in PBS for 15 min. Colony formation was shown by the number of stained colonies.

### Metabolic Oligosaccharide Engineering (MOE) of Living Cells

HuCCT1, HIBEpiC, HCCC-9810, and RBE cells seeded at 10 cm dishes were treated with unnatural sugar (1,6-Pr_2_GalNAz) at varied concentrations for 48 h or with 200 μM 1,6-Pr_2_GalNAz for up to 72 h when they reached approximately 30% confluence. The cells were harvested by trypsin digestion and washed twice with PBS and were lysed as described in the Protein Extraction and Western Blot Analysis section. All lysates were incubated with 500 μM premixed CuSO_4_/BTTAA (molar ratio 1:2), 100 μM alkyne-biotin, and 2.5 mM fresh sodium ascorbate for 2 h at room temperature. Subsequently, 5× loading buffer was added to the solution and boiled at 99 °C for 5 min. Equal amounts of proteins were detected with antibiotin (1:2000, A0303, Beyotime) by Western blot analysis. For confocal fluorescence microscopy imaging, the cells were seeded into 8-chamber at 1 × 10^4^ cells/well and treated with 200 μM 1,6-Pr_2_GalNAz for 48 h. The cells were washed twice with PBS, and then fixed with 4% paraformaldehyde (w/v), and permeabilized with 0.5% Triton-X 100 (v/v). Then, the cells were incubated with 50 μM premixed CuSO_4_/BTTAA (molar ratio 1:6), 50 μM alkyne-AZDye-488, and 2.5 mM fresh sodium ascorbate for 10 min at room temperature. For nucleus staining, cells were incubated with 5 μg/mL Hoechst 33342 at room temperature for 20 min. The cells were washed three times after each step. Finally, the cells were imaged by the Leica TCS SP5 laser scanning confocal system equipped with a × 63 oil immersion objective lens. For FACS analysis, the cells were seeded at 6-well plates and treated with 200 μM 1,6-Pr_2_GalNAz for 48 h when they reached approximately 30% confluence. The cells were trypsinized into single-cell suspension, rinsed with ice-cold PBS, and fixed in ice-cold 70% ethanol overnight. Then, the cells were incubated with 50 μM premixed CuSO_4_/BTTAA (molar ratio 1:6), 50 μM alkyne-biotin, and 2.5 mM fresh sodium ascorbate for 10 min on ice, followed by incubation with streptavidin-Alexa Fluor 488 Conjugate (1:2000, S32354, Thermo Fisher Scientific) for 30 min on ice, and then determined by flow cytometry (BD Biosciences).

### Glycopeptides Enrichment

The lysates extracted from HuCCT1 and HIBEpiC cells were incubated with 500 μM premixed CuSO_4_/BTTAA (molar ratio 1:2), 100 μM alkyne-PC-biotin, and 2.5 mM fresh sodium ascorbate for 3 h at room temperature. The mixture was added to 8 volumes of ice-cold methanol for precipitation overnight at −80 °C and washed three times with ice-cold methanol. Then, the protein pellet was reconstituted by sonication using 4 M urea in 50 mM ABC, incubated with 10 mM DTT at 37 °C for 1 h, and followed by incubation with 20 mM IAA at room temperature for 30 min in the dark. The solution was diluted to 1 M urea in 50 mM ABC supplemented with mass spectra grade trypsin (enzyme: substrate ratio at 1:50) and reacted at 37 °C for 16 h. After that, the streptavidin agarose beads (150 μL per 40 mg, Thermo Fisher Scientific, 20359) were added to the solution above and gently rotated for 3 h at room temperature. The beads were washed five times with PBS and Milli-Q water successively and resuspended with 200 μL 0.1% formic acid (FA, v/v), followed by irradiating three times under 365 nm UV light for 5 min using a UV cross-linker (CL-1000; UVP). The supernatant was collected, evaporated in a vacuum centrifuge, and subjected to LC–MS/MS analysis.

### LC–MS/MS Analysis and Data Processing

LC–MS/MS analysis of the glycopeptides enriched above was performed as previously described.^22^ In brief, all samples were resuspended with 0.1% FA, analyzed by an Orbitrap Fusion Lumos Tribrid Mass Spectrometer with a Nanospray Flex ionization source (Thermo Fisher Scientific), and coupled online to a nanoflow LC system (EASY-nLC 1200, Thermo Fisher Scientific). Survey scans of precursor were collected in Orbitrap from 350 to 2000 *m*/*z*, under the resolution of 120,000 at 200 *m*/*z*. Monoisotopic precursors selection was enabled, and the multicharged precursors with *z* = 2–8 were selected for data-dependent MS/MS scans with a cycle time of 3 s, dynamic exclusion set to 15 s, and window set to ±10 ppm. The initial data-dependent MS/MS scans were acquired using HCD with a first mass of 120 *m*/*z*, a normalized collision energy (NCE) of 30 ± 10, and a resolution of 30,000 at 200 *m*/*z*, according to the sceHCD-pd-EThcD method. Following ETD fragmentation triggered by glycan oxonium fragments, the glycan oxonium ions (*i.e*., *m*/*z* 168.0655, 186.0761, 204.0865, 274.0921, 292.1027, 300.1302, 329.1455, 342.1777, 366.1395, 388.1463, 399.1992, 405.213, etc.) were detected in the sceHCD spectrum with a mass accuracy within 10 ppm; meanwhile, additional precursor isolation and EThcD acquisition were performed with supplemental activation of 35.

The raw data processing was performed using pGlyco3 (https://github.com/pFindStudio/pGlyco3/releases/tag/pGlyco3.0.rc3_build20210124) under the “HCD + EThcD” mode as previously described.^22,23^ Briefly, the MS/MS spectrum were searched against the SwissPort *Human sapiens* proteome database downloaded from Uniprot (https://www.uniprot.org) on 2016-11-4. The *N*-glycans and *O*-glycans were searched against the pGlyco-*N*-glycan mode and the pGlyco-*O*-glycan mode in the pGlyco3 software with modified glycan databases, respectively. The parameter precursor tolerance was set to ±10 ppm and fragment tolerance ±20 ppm. The false discovery rate (FDR) was set to less than 1%. Notably, the *O*-GlcNAc sites were assigned manually based on the subcellular localization. The proteins localized in the cytoplasmic side (including the nucleus, cytoplasmic, mitochondrial, and cytoplasmic part of transmembrane proteins) were selected as *O*-GlcNAc proteins. For quantification, the *O*-GlcNAc sites coidentified in three independent replicates of HuCCT1 and HIBEpiC cells were defined as quantified *O*-GlcNAc sites. The *O*-GlcNAc sites with *P*-value < 0.05 and a fold change > 1.50 or < 0.67 were considered as up-regulated or down-regulated *O*-GlcNAc sites, respectively.

### Chemoenzymatic Labeling of *O*-GlcNAcylated Proteins

The cells were lysed as described in the Protein Extraction and Western Blot Analysis Section, and the lysates were added to eight volumes of ice-cold methanol overnight at −80 °C and washed three times with ice-cold methanol. The proteins were reconstituted with 1% SDS (w/v) in 20 mM HEPES buffer (pH 7.9) using sonication and incubated with 125 mM NaCl, 5% Nonidet P-40 (NP-40, v/v), 50 mM HEPES (pH 7.9), 100 mM MnCl_2_, 500 μM UDP-GalNAz, and Y289L GalT1 (enzyme: substrate ratio at 1:40) at 4 °C for 20 h. Methanol, chloroform, and Milli-Q water are successively added to the solution above (solution: methanol: chloroform: Milli-Q water ratio at 1:3:0.75:2) to obtain a protein pellet, followed by washing three times with ice-cold methanol. The proteins were resuspended as described above.

### Immunoprecipitation Assay

For biotin immunoprecipitation, the cells were labeled and lysed as mentioned above. The biotinylated lysates were treated with streptavidin agarose beads (10 μL/mg) and gently rotated for 3 h at room temperature. For anti-FLAG immunoprecipitation, the cells transfected with indicated plasmids were labeled and lysed as mentioned above. Then, the biotinylated lysates were treated with anti-FLAG beads (10 μL/mg, M20038, Abmart) at 4 °C for 12 h with gentle rotation. For K18 immunoprecipitation, the cells transfected with ncRNA, siOGT-1, or siOGA-1 were lysed as mentioned above, and then the lysates were diluted 10 times using RIPA lysis buffer supplementing with protease inhibitor. Four micrograms of anti-K18 antibody was incubated with 1 mg cell lysates at 4 °C for 12 h with gentle rotation. Immune complexes were retrieved by Protein A/G-Agarose beads (sc-2003, Santa Cruz) that gently rotated for 3 h at 4 °C. The beads were washed five times with PBS, added with 5× loading buffer, and boiled at 99 °C for 5 min. The final immunoprecipitated proteins were analyzed by Western blot.

### *O*-GlcNAcylation Stoichiometry on K18

The cells treated with metabolic oligosaccharide engineering or chemoenzymatic labeling were incubated with 500 μM premixed CuSO_4_/BTTAA (molar ratio 1:2), 100 μM alkyne-PEG_5KD_, and 2.5 mM fresh sodium ascorbate at 37 °C for 16 h. Subsequently, 5× loading buffer was added to the solution and boiled at 99 °C for 5 min. Equal amounts of proteins were detected with anti-K18 (Santa Cruz) by Western blot analysis.

### Plasmids Construction

The FLAG-tagged K18 constructs were generated by in-frame subcloning the human *KRT18* cDNA into the pFLAG-CMV-2 vector (Sigma-Aldrich). The K18 mutants (S15A, S18A, S15/18A, S30A, S31A, S30/31A and 4A), HA-tagged K18 constructs (WT and S30A), and FLAG-tagged IDH2 construct (S202A) were generated using the ClonExpress Ultra One Step Cloning Kit (Vazyme) according to the manufacturer’s protocol. All constructs were confirmed by DNA sequencing (Sangon, Shanghai, China). All primers used in plasmid construction were provided in this study (Table S4).

### Immunofluorescence Assay

Cells seeded into 8-chamber at 1 × 10^4^ cells/well were washed twice with PBS, fixed with 4% paraformaldehyde (w/v) for 15 min, and then permeabilized with 0.2% Triton-X 100 (v/v) for 10 min. Subsequently, the cells were blocked with 5% BSA for 1 h at room temperature. Primary anti-FLAG-K18 antibody was added for incubation overnight at 4 °C, followed by Alexa Fluor 488-conjugated secondary antibody incubation at room temperature for 1 h. For nucleus staining, cells were incubated with 5 μg/mL Hoechst 33342 at room temperature for 20 min. The cells were washed three times after each step. Images were acquired by the Leica TCS SP5 laser scanning confocal system equipped with a ×63 oil immersion objective lens.

### Lentivirus Infection and Stable Cell Line Establishment

The lentiviruses with random small hairpin RNA (shNC, used as a negative control) and small hairpin RNA K18 knockdown (shK18-1-3) were generated by GenePharma (Shanghai, China) using the LV-U6-copGFP-T2A-Neo vector (GenePharma) according to the manufacturer’s instructions. The HuCCT1 cells were infected with these lentiviruses and selected for stable cell lines with 400 μg/mL G418 for 2 weeks. We screened the lentivirus with the highest efficiency of knockdown of endogenous K18 (shK18) by qRT-PCR and Western blot analysis. To generate K18 reconstituted stable cell lines, we used the coexpressing exogenous FLAG-K18 WT or FLAG-K18 S30A lentivirus which was constructed with LV8N vector (Mock, GenePharma) to infect the shK18 HuCCT1 cells and selected for stable cell lines with 2 μg/mL puromycin for 2 weeks. The successful construction of stable cell lines shNC + Mock, shK18, shK18 + WT, and shK18 + S30A were verified by qRT-PCR and Western blot analysis. For CCK-8 analysis, the cells were seeded into 96-well plates at a density of 5 × 10^3^ cells/well, and the viability of cells was determined using the CCK-8 assays (Vazyme) following the manufacturer’s instructions. Cell cycle distribution of the cells above was by FACS analysis as described in the Cell Cycle Analysis section. For colony formation, the cells were seeded into 6-well plates at a density of 200 cells/well and detected as described in the Colony Formation Assay section.

### Determination of K18 Half-Life

The degradation of K18 in HuCCT1, RBE, and HCCC-9810 cells incubated with DMSO, 200 μM 5S, or 1 μM TMG for 48 h followed by treatment with 10 μg/mL CHX for 0, 2, 4, or 8 h was determined by Western blot analysis. The stable cell lines (shK18 + WT and shK18 + S30A) were treated with 10 μg/mL CHX for 0 or 8 h. The FLAG-K18 protein levels were analyzed by Western blot.

### Ubiquitination Assay

The stable cell lines (shK18 + WT and shK18 + S30A) were transfected with HA-ubiquitin and treated with 0 or 5 μM MG-132 for 20 h. Then, the cells were lysed and captured with anti-FLAG beads. Anti-HA blot demonstrated the ubiquitination of immunoprecipitated FLAG-K18.

### Tumorigenesis in Nude Mice

Five-week-old BALB/c nude mice (male) were purchased from GemPharmatech (Nanjing, China) and housed under specific pathogen-free (SPF) conditions. All animal experimental procedures were approved by the Animal Care and Use Committee of Nanjing University. Specifically, BALB/c nude mice as mentioned above were randomly divided into four groups (*n* = 6 per group) and injected subcutaneously with 5 × 10^6^ HuCCT1 stable cells with shNC + Mock, shK18, shK18 + WT, and shK18 + S30A K18. shNC + Mock was the control group. Tumors generated by xenograft HuCCT1 stable cells were measured every 6 days from day 10. After 34 days, tumors were dissected, photographed, weighed, and subjected to immunohistochemistry analysis of the Ki67 protein.

### Analysis of *O*-GlcNAcylated K18 in Tissues

The tissues of xenograft tumors generated from HuCCT1 cells (shK18 + WT and shK18 + S30A) and patient samples were ground to single cells and lysed as described in the Protein Extraction and Western Blot Analysis section. Then, the lysates were biotinylated by chemoenzymatic labeling and captured with streptavidin beads. The *O*-GlcNAcylation of K18 was determined by Western blot analysis.

### Interacting Proteins Analysis

The lysates from shK18 cells transfected with FLAG-K18^WT^ or FLAG-K18^S30A^ were captured with anti-FLAG beads and analyzed by LC–MS/MS. For protein quantification, the fold change from three independent replicates of the interacting proteins with K18 in FLAG-K18^WT^ cells was calculated relative to FLAG-K18^S30A^ cells. Proteins with *P*-value < 0.05 and a fold change > 1.50 or < 0.67 were considered as up-regulated or down-regulated interacting proteins, respectively. KEGG enrichment analysis for the upregulated interacting proteins was performed using the Database for Annotation, Visualization, and Integrated Discovery (DAVID) bioinformatics resources.

### Mitochondria Isolation

The mitochondria isolation experiment was carried out using the cell mitochondria isolation kit (Beyotime) according to the manufacturer’s instructions. In brief, cells seeded into the 10 cm dish were trypsinized and washed with ice-cold PBS. Subsequently, 2 × 10^6^ cells were lysed as mentioned above, the remaining 8 × 10^6^ cells were suspended gently in 1 mL mitochondria isolation reagent containing PMSF and incubated on ice for 15 min. Then the cell suspension was homogenized by a glass homogenizer, during which the trypan blue staining solution was used to judge the homogenization efficiency. After centrifugation at 600*g* for 10 min, the supernatant was transferred to the new tube and continued to be centrifuged at 3500*g* for 10 min. The pellet from this step was the isolated mitochondria which was lysed using 100 μL of mitochondrial lysis buffer containing PMSF. After centrifugation at 12,000*g* for 10 min, the final supernatant was the cytoplasmic protein which mitochondria have been removed. The whole cell lysates, mitochondrial, and cytoplasmic proteins were subjected to Western blot analysis.

### Molecular Docking

The structure of IDH2 was developed by homology modeling with 5I96 (RCSB PDB, https://www.rcsb.org/) as a template on the SWISS-MODEL server (https://swissmodel.expasy.org/). The nonglycosylated K18 (ID: AF-P05783-F1) was taken from the modeling structure of the AlphaFold Protein Structure Database (https://alphafold.ebi.ac.uk/).^60,61^ The structure of glycosylated K18 (*O*-GlcNAcylated at Ser30) was modeled with the Glycoprotein Builder of GlyCAM server (https://glycam.org/).^62^ The molecular docking of complex structure for IDH2-nonglycosylated K18 and IDH2-glycosylated K18 (*O*-GlcNAcylated at Ser30) were performed by ZDOCK 3.0.2f on the ZDOCK server (https://zdock.umassmed.edu/). PDBsum (https://www.ebi.ac.uk/thornton-srv/databases/pdbsum/) was used to analyze further interaction types of the complex structure on the interaction interface.^63^

### Measurement of Intracellular ATP Levels

Glycolytic ATP and total ATP were measured using an ATP Quantitation Kit (Beyotime) after treating with or without 2.5 μM oligomycin for 3 h to assess OXPHOS ATP levels. Briefly, cells were lysed with ice-cold lysis buffer, and after centrifugation at 12000*g* for 5 min, the supernatants were added into ATP detection solution containing substrate and luciferase and incubated at room temperature for 5 min. The luminescence was detected by a microplate reader (TECAN Infinite M1000 Pro). A standard curve of ATP concentration was prepared from the measurement of standard solutions. Meanwhile, the supernatants were quantified for protein concentration using a BCA kit. The quantity of intracellular ATP was normalized to the corresponding protein.

### Analysis of Metabolites by LC–MS/MS

The extraction and analysis of cell metabolic products were carried out according to the previous procedures.^64^ The cells seeded into the 10 cm dish were cultured in RPMI 1640 medium supplementing with 10% FBS, 100 U/mL of penicillin, and streptomycin. For labeling experiments, the cells were washed twice with PBS and incubated with glucose-free RPMI 1640 medium (Gibco, cat. no. 11879020) supplementing with 10% FBS, containing 11.1 mM label-free glucose (Sigma-Aldrich) or 11.1 mM [U-^13^C_6_] glucose (Cambridge Isotope Laboratories) for 8 h. An unlabeled culture was prepared in parallel by adding equal concentrations of label-free glucose to the medium to identify unlabeled metabolites. The medium of cells seeded at the 10 cm dish was absorbed completely by a pump. The cells were rinsed with precooled PBS three times, and the dish was immediately placed on dry ice with 80% precooled methanol (v/v) and incubated at −80 °C for 1 h. Subsequently, the cells were scraped off on dry ice using a cell scraper, and the lysates were centrifuged at 14,000*g* for 20 min at 4 °C to obtain the supernatant-containing metabolites. The pellets were lysed and quantified as described in the Protein Extraction and Western Blot Analysis section, and the supernatant containing metabolites was dried into powder using SpeedVac and lyophilize. The samples were resuspended using 100 μL 50% methanol (v/v). After centrifugation, 1 μL supernatant was injected into the Q Exactive mass spectrometer (Thermo Fisher Scientific) for detection. For each metabolite, the standard compound was detected to ensure proper chromatographic elution time and generate a standard curve. LC–MS/MS analysis was performed as previously described.^65,66^ Data were acquired and processed using Tracefinder software. The quantity of the metabolite fraction was normalized to the corresponding protein.

### Measurement of Intracellular ROS Levels

ROS levels were measured using a fluorescent ROS indicator, 2′,7′-dichlorofluorescein diacetate (H2DCF-DA, Beyotime). Briefly, cells were seeded into 6-well plates at a density of 4 × 10^5^ cells/well, followed by exposure to 1 μM TMG for 48 h or 500 μM H_2_O_2_ for 12 h. After the treatment, cells were washed three times with PBS and incubated with 5 μM DCFH-DA in the dark at 37 °C for 20 min. Subsequently, the cells were harvested for ROS analysis and were determined by flow cytometry (Agilent) within 1 h.

### Measurement of Intracellular NADP^+^ and NADPH Levels

Intracellular NADP^+^ and NADPH levels were determined by a NADP^+^/NADPH Quantitation Kit (Beyotime). Briefly, cells were seeded into 6-well plates at a density of 4 × 10^5^ cells/well, followed by exposure to 1 μM TMG for 48 h. After incubation, the cells were lysed using 200 μL NADP^+^/NADPH extraction buffer and centrifuged at 12,000*g* for 10 min. For NADPH levels analysis, the 100 μL of supernatants were heated at 60 °C for 30 min to remove NADP^+^. To analyze the total NADP^+^/NADPH levels and NADPH levels, 50 μL of samples were added into 100 μL of NADP^+^/NADPH reaction mixture and incubated in the dark at 37 °C for 10 min. Subsequently, 10 μL of chromogenic solution was added to the reaction mixture and incubated in the dark at 37 °C for 10 min. The samples were then measured at a wavelength of 450 nm. A standard curve of NADPH concentration was prepared from the measurement of standard solutions. Meanwhile, the supernatants were quantified for protein concentration using a BCA kit. The quantity of intracellular NADP^+^ and NADPH levels was normalized to the corresponding protein.

### Quantification and Statistical Analysis

Data from three independent experiments are shown as the mean ± standard deviation (SD). Student’s *t* tests (two-tailed) were used to compare two data sets, and a *P*-value of < 0.05 was considered statistically significant (*P < 0.05, **P < 0.01, ***P < 0.001, and ****P < 0.0001, ns, not significant). Otherwise, the *P*-value of the Kaplan–Meier survival curve (Figure S3c,d) was analyzed by the log-rank (Mantel-Cox) test, which is a hypothesis test to compare the survival distributions of two samples. The adjusted *P*-value (adjusted by a conservative Bonferroni correction) of motif analysis in *O*-GlcNAc sites, *N*-glycosites, and mucin-type *O*-glycosites was analyzed using the pLogo (https://plogo.uconn.edu/) (Figures S16b and S17d). GO terms (Figure S16c) and KEGG-enrichment analysis (Figure 5b) were performed using the DAVID bioinformatics resources (https://david.ncifcrf.gov/), and the enrichment *P*-values were adjusted by a modified Fisher’s exact test.

## Data Availability

The human cholangiocarcinoma data (Figure S3a,b) were derived from the GEO (https://www.ncbi.nlm.nih.gov/geo/query/acc.cgi) and TCGA Research Network (https://www.cancer.gov/ccg/research/genome-sequencing/tcga). The mass spectrometry data have been deposited to ProteomeXchange Consortium (http://proteomecentral.proteomexchange.org) via the PRIDE partner repository43 with the data set identifier PXD048188 and PXD048144. All other data supporting the findings of this study are available from the corresponding author upon reasonable request.

## Supporting Information Available

Figures S1−S39 (PDF)
List of *O*-GlcNAc sites identified in HuCCT1 and HIBEpiC cells (XLSX)
List of dysregulated *O*-GlcNAc sites identified in HuCCT1 and HIBEpiC cells (XLSX)
List of upregulated proteins interacting with K18 enriched in TCA cycle pathway (XLSX)
Resource and sequence of primers used in this study (XLSX)
Transparent Peer Review report available (PDF)
