# Correction to “*O*-GlcNAcylation Facilitates the Interaction between Keratin 18 and Isocitrate Dehydrogenases and Potentially Influencing Cholangiocarcinoma Progression”

**DOI:** 10.1021/acscentsci.4c01302

**Published:** 2024-10-21

**Authors:** Xiangfeng Meng, Yue Zhou, Lei Xu, Limu Hu, Changjiang Wang, Xiao Tian, Xiang Zhang, Yi Hao, Bo Cheng, Jing Ma, Lei Wang, Jialin Liu, Ran Xie

**Description of correction:**

Page 1070. The Western Blot figure of FLAG-K18 in Figure 3f was misused of the published article. It is revised in the corrected version of Figure 3.

**Figure 3:**
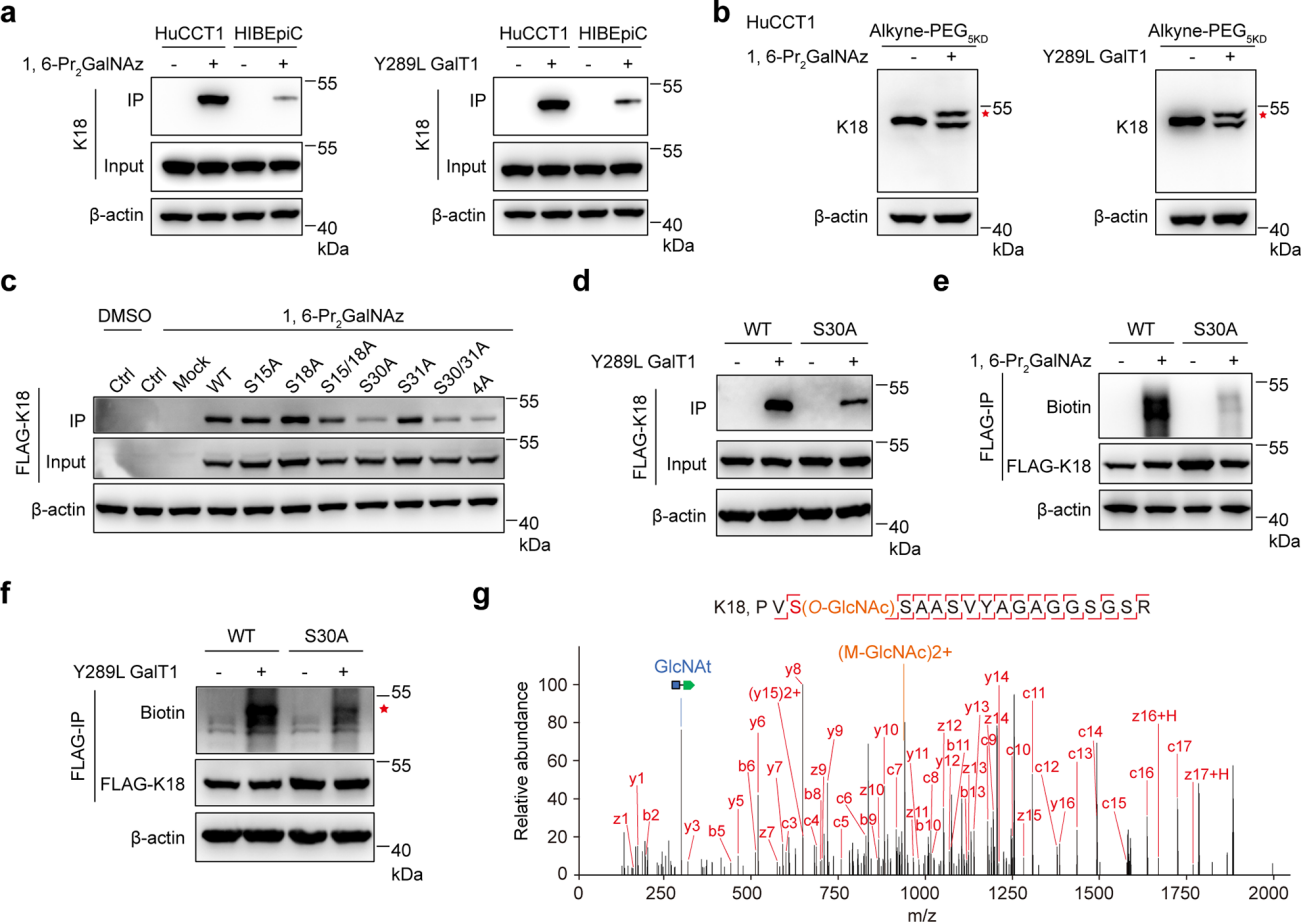
Keratin 18 is mainly *O*-GlcNAcylated at Ser 30. (a) Western blot analysis showing the Keratin 18 (K18) *O*-GlcNAcylation in HuCCT1 and HIBEpiC cells. The cells were incubated with 1,6-Pr_2_GalNAz, lysed, reacted with alkyne-biotin, and captured by streptavidin beads (left) or incubated with Y289L GalT1 and UDP-GalNAz in cell lysates, reacted with alkyne-biotin, and captured by streptavidin beads (right). (b) Western blot analysis showing the *O*-GlcNAcylation stoichiometry of K18 in HuCCT1 cells. The cells were incubated with 1,6-Pr_2_GalNAz, lysed, reacted with alkyne-PEG_5KD_ (left) or incubated with Y289L GalT1 and UDP-GalNAz in cell lysates, and reacted with alkyne-PEG_5KD_ (right). The red asterisk indicated tagged *O*-GlcNAcylated K18. (c) Immunoblot analysis of K18 *O*-GlcNAcylation showing HEK293T cell lysates transfected with FLAG-tagged K18 (FLAG-K18) with wild-type, single, double, or quadruple mutations incubated with 1,6-Pr_2_GalNAz, lysed, and immunoprecipitated with streptavidin beads. (d) Immunoblot analysis showing HEK293T cells overexpressing FLAG-K18^WT^ or FLAG-K18^S30A^ incubated with Y289L GalT1 and UDP-GalNAz and immunoprecipitated with streptavidin beads. (e) Immunoblot analysis showing the HEK293T cells overexpressing FLAG-K18^WT^ or FLAG-K18^S30A^ incubated with 1,6-Pr_2_GalNAz, lysed, reacted with alkyne-biotin, immunoprecipitated with anti-FLAG beads, and blotted with antibiotin. (f) Immunoblot analysis showing the HEK293T cells overexpressing FLAG-K18^WT^ or FLAGK18^S30A^ incubated with Y289L GalT1 and UDP-GalNAz, immunoprecipitated with anti-FLAG beads, and blotted with antibiotin. The red asterisk indicated tagged *O*-GlcNAcylated K18. (g) Representative MS2 spectrum of an *O*-GlcNAcylated peptide from K18 located on Ser 30. The matched fragment ions (red), diagnostic fragment ion (orange), and the GlcNAt fragment ion (blue) are labeled. Equal loadings were confirmed using β-actin in all Western blot analyses. IP, immunoprecipitation; UDP-GalNAz, UDP-N-azidoacetylglucosamine; Y289L GalT1, β-1,4-galactosyltransferase mutant; alkyne-PEG_5KD_, alkynylated poly(ethylene glycol) 5000.

**Statement:**

The conclusions of this work have not been affected.

