# Supplementary material for: O-GlcNAcylation Facilitates the Interaction between Keratin 18 and Isocitrate Dehydrogenases and Potentially Influencing Cholangiocarcinoma Progression
Source: ACS Cent Sci. 2024 Apr 23;10(5):1065–83. doi: 10.1021/acscentsci.4c00163 (PMC11117311; doi:10.1021/acscentsci.4c00163)
Supplement: Supplementary file 1 — oc4c00163_si_001.pdf [file oc4c00163_si_001.pdf]

Supplementary Information

O-GlcNAcylation Facilitates the Interaction between Keratin 18 and Isocitrate Dehydrogenases and Potentially Influencing Cholangiocarcinoma Progression

Xiangfeng Meng,○† Yue Zhou,○‡ Lei Xu,‡ Limu Hu,†⊥ Changjiang Wang,† Xiao Tian,† Xiang Zhang,‡ Yi Hao,# Bo Cheng,▽ Jing Ma*†⊥ Lei Wang,*‡ Jialin Liu*§ and Ran Xie*†∥◇

†State Key Laboratory of Coordination Chemistry, School of Chemistry and Chemical Engineering, Nanjing University, Nanjing 210023, China.

‡Department of Gastroenterology, Nanjing Drum Tower Hospital, The Affiliated, Hospital of Nanjing University Medical School, Nanjing 210008, China.

§State Key Laboratory of Medical Proteomics, Beijing Proteome Research Center, National Center for Protein Sciences (Beijing), Beijing Institute of Lifeomics, Beijing 102206, China.

∥Chemistry and Biomedicine Innovation Center (ChemBIC), Nanjing University, Nanjing 210023, China.

⊥Collaborative Innovation Center of Advanced Microstructures, Nanjing University, Nanjing 210023, China.

#College of Chemistry and Molecular Engineering, Peking University, Beijing 100871, China.

▽School of Pharmaceutical Sciences, Peking University, Beijing 100191, China.

◇Beijing National Laboratory for Molecular Sciences, Beijing 100191, China.

○These authors contributed equally: Xiangfeng Meng, Yue Zhou.

*Correspondence:

ranxie@nju.edu.cn (Ran Xie)

jialinliu_bprc@163.com (Jialin Liu)

leiwang9631@nju.edu.cn (Lei Wang)

majing@nju.edu.cn (Jing Ma)

This Supplementary Information includes **Figures S1-S39**:

Figure S1. *O*-GlcNAcylation and OGT are upregulated in human CCA tumor tissues.

Figure S2. *O*-GlcNAcylation is dysregulated in human CCA.

Figure S3. *OGT* and *OGA* mRNA expression are dysregulated in CCA and correlate with overall survival (OS).

Figure S4. *OGT* and *OGA* mRNA expression are dysregulated in CCA cell lines.

Figure S5. Global *O*-GlcNAcylation levels in CCA cells are regulated by 5S and TMG treatment.

Figure S6. Regulation of *O*-GlcNAcylation affects cell proliferation in CCA cells.

Figure S7. Cell apoptosis assay of 5S- or TMG-treated HuCCT1 cells.

Figure S8. Cell apoptosis assay of 5S- or TMG-treated RBE cells.

Figure S9. *O*-GlcNAcylation affects the cell cycle progression of CCA cells.

Figure S10. Western blot analysis of the cell cycle and apoptosis marker in 5S- or TMG-treated RBE cells.

Figure S11. Knockdown of *OGT* reduced CCA cell proliferation.

Figure S12. Global *O*-GlcNAcylation of the CCA cells is affected by regulating OGT and OGA levels.

Figure S13. Cytotoxicity of 1,6-Pr_2GalNAz in CCA and HIBEpiC cells by CCK-8 assays.

Figure S14. Metabolic efficacy of 1,6-Pr_2GalNAz in HCCC-9810 and RBE cells.

Figure S15. Streamlined pipeline for screening high-confidence intact glycosites.

Figure S16. Profiling of protein *O*-GlcNAcylation in HuCCT1 and HIBEpiC cells.

Figure S17. Profiling of the intact glycosites in HuCCT1 and HIBEpiC cells.

Figure S18. Numbers of the intact glycosites with specific glycan in HuCCT1 and HIBEpiC cells.

Figure S19. Glycan compositions from the intact glycosites in HuCCT1 and HIBEpiC cells.

Figure S20. Biochemical validation of identified *O*-GlcNAcylated proteins in HuCCT1 and HIBEpiC cells.

Figure S21. Knockdown of *OGT* reduces the *O*-GlcNAcylated K18 protein levels.

Figure S22. *O*-GlcNAcylation stoichiometry of K18 by Western blot analysis.

Figure S23. *O*-GlcNAc sites of K18 identified in HuCCT1 and HIBEpiC cells.

Figure S24. MS2 spectrum analysis of *O*-GlcNAcylated peptide in K18.

Figure S25. Confocal fluorescence imaging of K18 filament organization in RBE cells.

Figure S26. Generation of HuCCT1 stable cell lines.

Figure S27. *O*-GlcNAcylation of K18 promotes cell growth in RBE cells.

Figure S28. *O*-GlcNAcylation of K18 promotes cell growth in HCCC-9810 cells.

Figure S29. Tumor formation in nude mice.

Figure S30. K18 and its *O*-GlcNAcylation are dysregulated in CCA.

Figure S31. Analysis of interacting proteins of K18 protein.

Figure S32. Analysis of enzymes in the TCA cycle interacting with K18 of RBE stable cell lines.

Figure S33. Distribution of K18 protein in CCA cells.

Figure S34. Analysis of IDH(s) interacted with K18 in CCA cells.

Figure S35. Structural modeling and simulation of IDH2-nonglycosylated K18 binding and IDH2-glycosylated K18 (*O*-GlcNAcylated at Ser30) binding.

Figure S36. Schematic diagrams of the interactions for IDH2-nonglycosylated K18 binding and IDH2-glycosylated K18 (*O*-GlcNAcylated at Ser30) binding.

Figure S37. Ser202 of IDH2 contributes to its interaction with K18 in CCA cells.

Figure S38. Measurement of glycolytic and oxidative phosphorylation (OXPHOS) ATP levels in CCA cells.

Figure S39. *O*-GlcNAcylation of K18 contributes to the resistance of H_2O_2 stimulation in CCA cells.

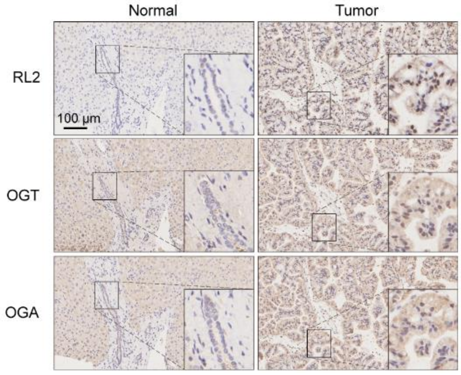

Figure S1. *O*-GlcNAcylation and OGT are upregulated in human CCA tumor tissues. Representative images of RL2 (anti-*O*-GlcNAc), OGT, and OGA staining by immunohistochemistry (IHC). Scale bar: 100 μm.

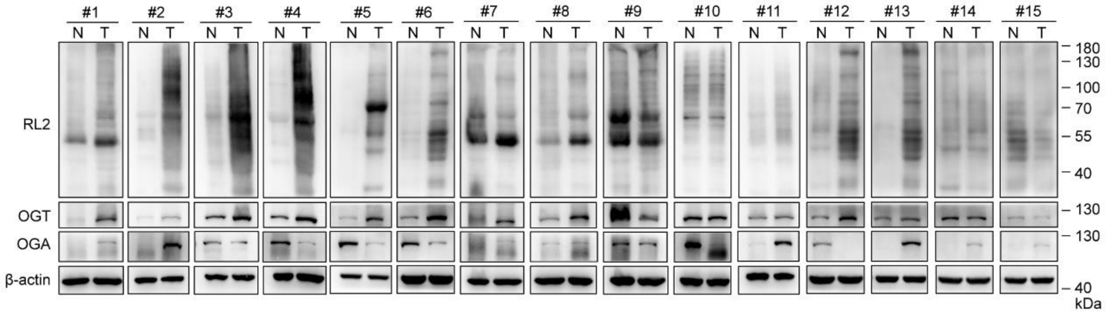

Figure S2. *O*-GlcNAcylation is dysregulated in human CCA. Western blot analysis of *O*-GlcNAcylated proteins, OGT, and OGA levels in the 15 CCA tumor tissues (T) and adjacent normal tissues (N). Equal loading was confirmed using β-actin.

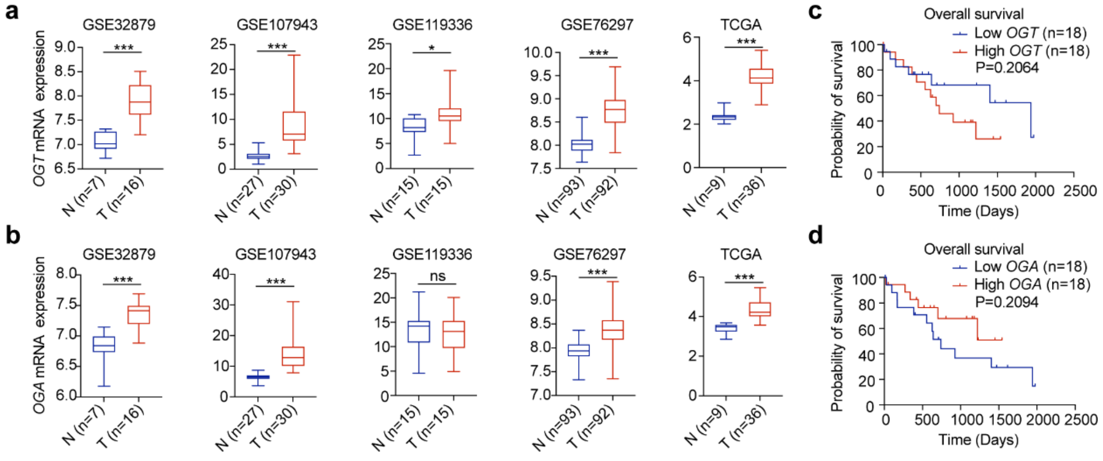

Figure S3. *OGT* and *OGA* mRNA expression are dysregulated in CCA and correlate with overall survival (OS). (a-b) The *OGT* (a) and *OGA* (b) mRNA expression in CCA tumor tissues and adjacent normal tissues based on GSE32879, GSE107943, GSE119336, GSE76297, and TCGA datasets. (c-d) Kaplan-Meier survival curve in the high and low *OGT* (c) or *OGA* (d) mRNA expression groups. Data (Figure S3a,b) were shown as the mean ± standard deviation (SD); statistical significance was determined by Student's *t* tests (two-tailed, **P* < 0.05 and ****P* < 0.001, ns, not significant). The *P*-value of the Kaplan-Meier survival curve (Figure S3c,d) was analyzed by log-rank (Mantel-Cox) test.

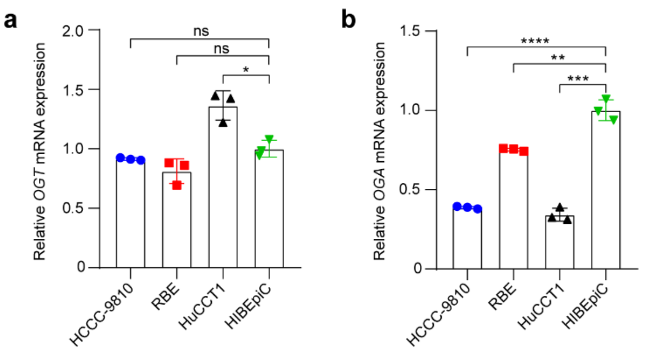

Figure S4. *OGT* and *OGA* mRNA expression are dysregulated in CCA cell lines. (a-b) The relative expression level of *OGT* (a) and *OGA* (b) mRNA in three CCA cells and HIBEpiC cells was determined by quantitative real-time PCR (qRT-PCR). Data were shown as the mean ± SD; statistical significance was determined by Student's *t* tests (two-tailed, $*P < 0.05$, $**P < 0.01$, $***P < 0.001$ and $****P < 0.0001$, ns, not significant).

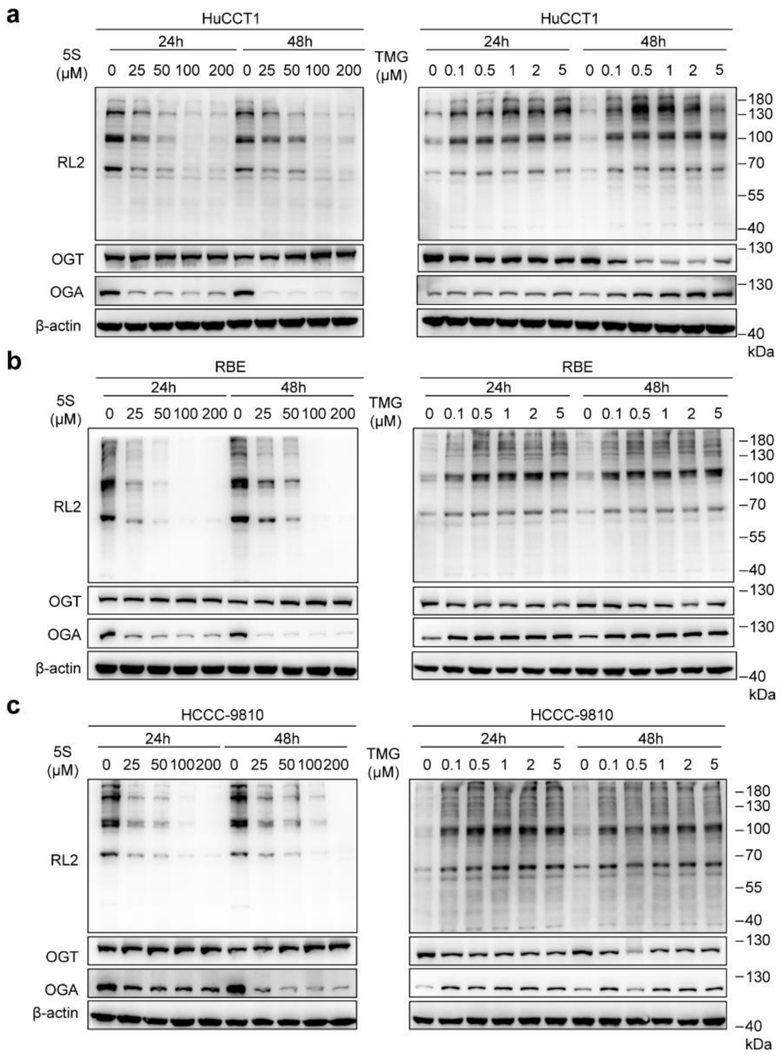

Figure S5. Global *O*-GlcNAcylation levels in CCA cells are regulated by 5S and TMG treatment. (a-c) Time and dose-dependent analysis of the *O*-GlcNAcylated protein, OGT, and OGA levels in 5S- or TMG-treated HuCCT1 (a), RBE (b), and HCCC-9810 (c) cells by Western blot. Equal loading was confirmed using β-actin.

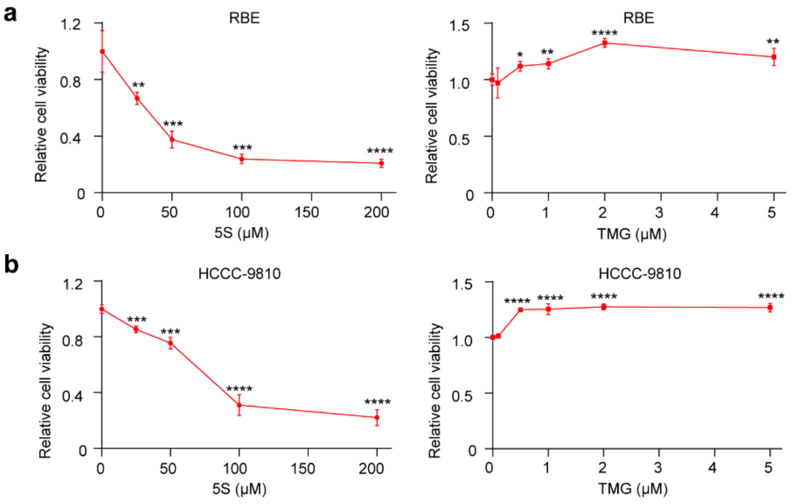

Figure S6. Regulation of *O*-GlcNAcylation affects cell proliferation in CCA cells. (a-b) Cytotoxicity assay of 5S- or TMG-treated RBE (a) and HCCC-9810 (b) cells. The cells were treated with 5S or TMG at various concentrations for 48 h. Data were shown as the mean ± SD; statistical significance was determined by Student's *t* tests (two-tailed, *$P < 0.05$, **$P < 0.01$, ***$P < 0.001$ and ****$P < 0.0001$).

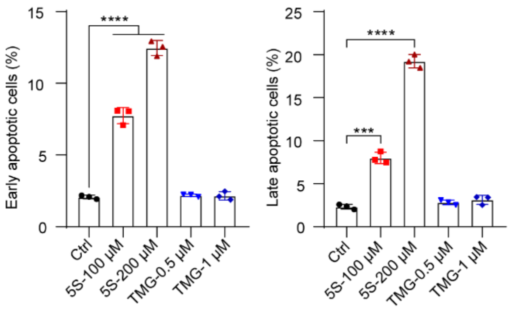

Figure S7. Cell apoptosis assay of 5S- or TMG-treated HuCCT1 cells. The quantitative analysis for the cell population of early apoptotic cells ($FITC^+/PE^-$) and late apoptotic cells ($FITC^+/PE^+$) in 5S- or TMG-treated HuCCT1 cells. Data were shown as the mean ± SD; statistical significance was determined by Student's *t* tests (two-tailed, ***P < 0.001 and ****P < 0.0001).

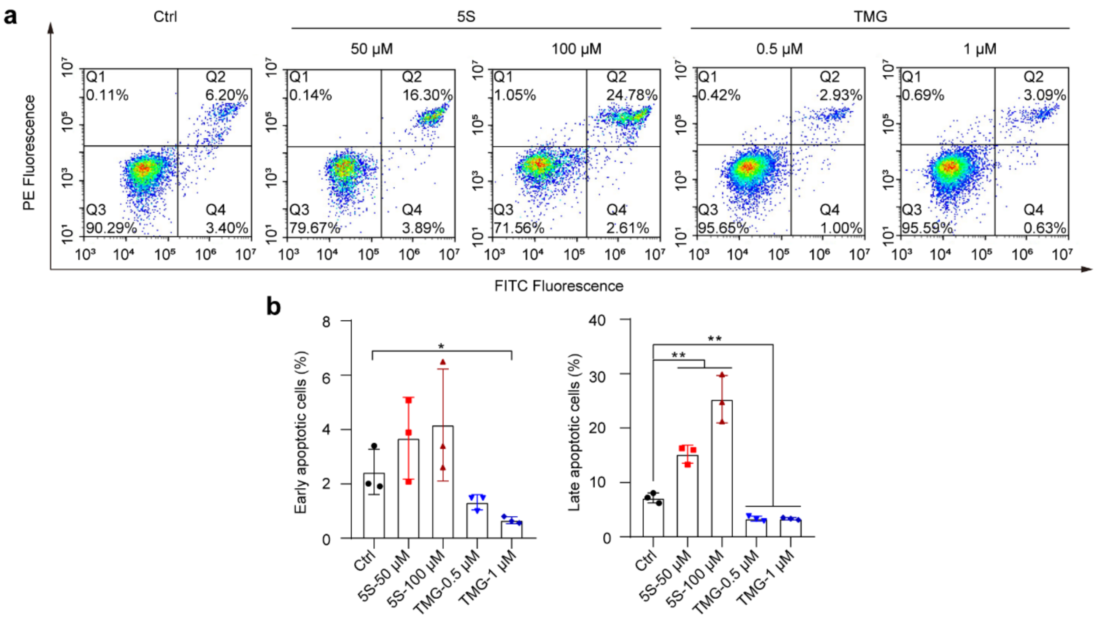

Figure S8. Cell apoptosis assay of 5S- or TMG-treated RBE cells. (a) The bivariate density plot in flow cytometry indicated the cell population of early apoptotic cells ($FITC^+/PE^-$) and late apoptotic cells ($FITC^+/PE^+$). (b) The quantitative analysis for the cell population of early apoptotic cells ($FITC^+/PE^-$) and late apoptotic cells ($FITC^+/PE^+$) in 5S- or TMG-treated RBE cells. Data were shown as the mean ± SD; statistical significance was determined by Student's *t* tests (two-tailed, **P* < 0.05 and ***P* < 0.01).

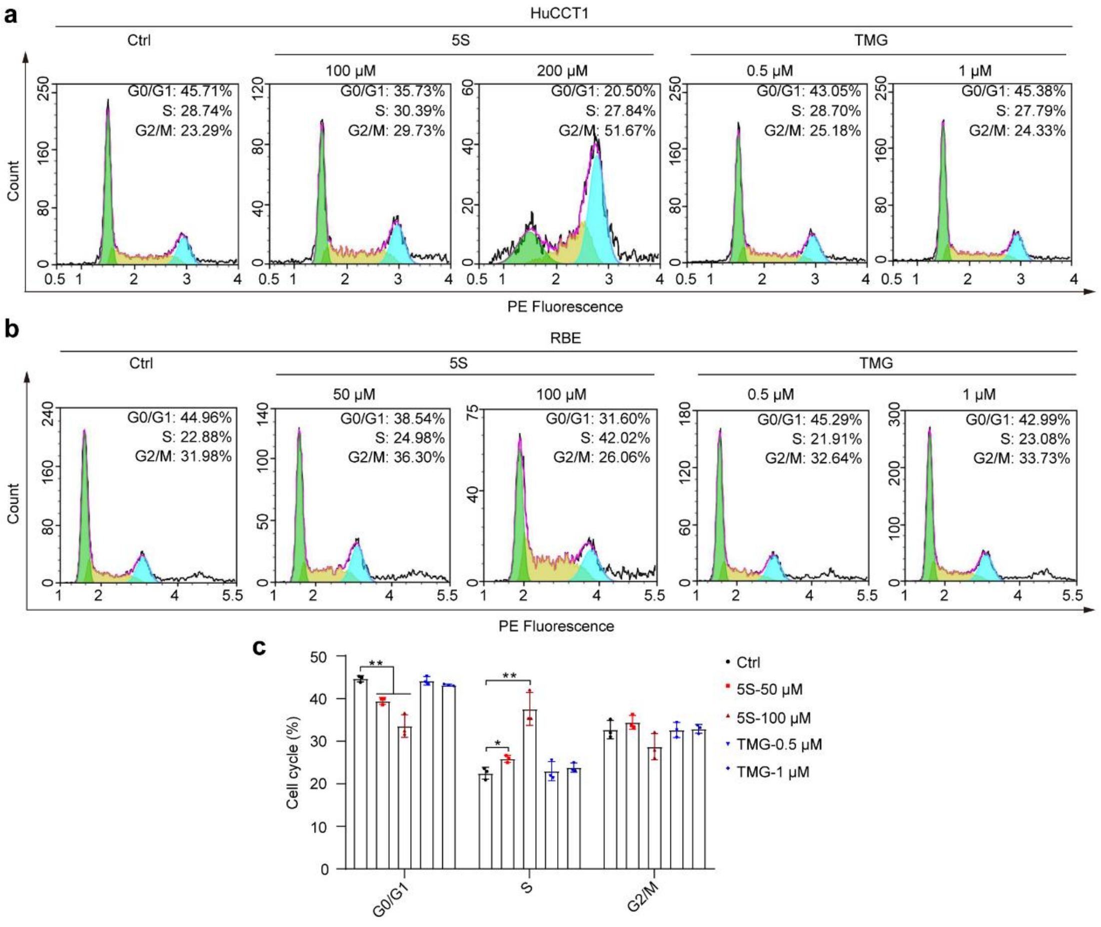

Figure S9. *O*-GlcNAcylation affects the cell cycle progression of CCA cells. (a) Cell cycle distribution assay of 5S- or TMG-treated HuCCT1 cells. (b) Cell cycle distribution assay of 5S- or TMG-treated RBE cells. (c) Histogram plot in flow cytometry indicated the percentage of cell populations in the G0/G1, S, or G2/M phase of RBE cells. Data were shown as the mean ± SD; statistical significance was determined by Student's *t* tests (two-tailed, *$P < 0.05$ and **$P < 0.01$).

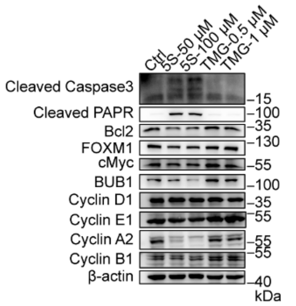

Figure S10. Western blot analysis of the cell cycle and apoptosis marker in 5S- or TMG-treated RBE cells. Protein levels of cleaved Caspase3, cleaved PARP (apoptotic markers); Bcl2 (antiapoptotic marker); cMyc, Cyclin D1, FOXM1, Cyclin E1(G1/S transition markers); and BUB1, Cyclin A2, Cyclin B1(G2/M transition markers) were analyzed by Western blot. Equal loading was confirmed using β-actin.

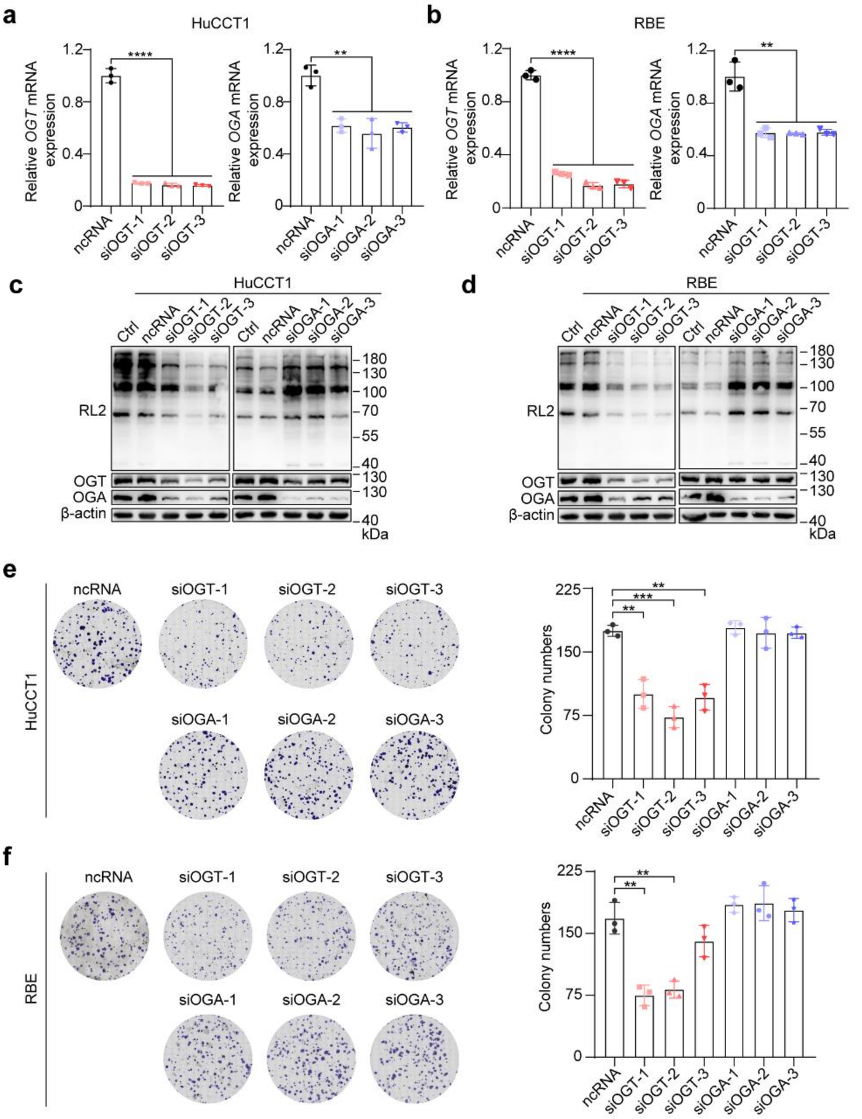

Figure S11. Knockdown of *OGT* reduced CCA cell proliferation. (a-b) The *OGT* or *OGA* mRNA expression of small interfering RNAs (siRNAs)-treated HuCCT1 (a) and RBE (b) cells by qRT-PCR. Random RNA was used as the negative control. Three independent siRNAs targeting *OGT* (siOGT1-3) or *OGA* (siOGA1-3) were shown. (c-d) Silencing efficacy of siOGT or siOGA in HuCCT1 (a) and RBE (b) cells. The *O*-GlcNAcylated protein, OGT, and OGA levels were analyzed by Western blot. Equal loading was confirmed using β-actin. (e-f) Clonogenic assay of HuCCT1 (e) and RBE (f) cells transfected with ncRNA, siOGTs, or siOGAs. Colony numbers were quantified in the right panel. Data were shown as the mean ± SD; statistical significance was determined by Student's *t* tests (two-tailed, $^{**}P < 0.01$, $^{***}P < 0.001$ and $^{****}P < 0.0001$).

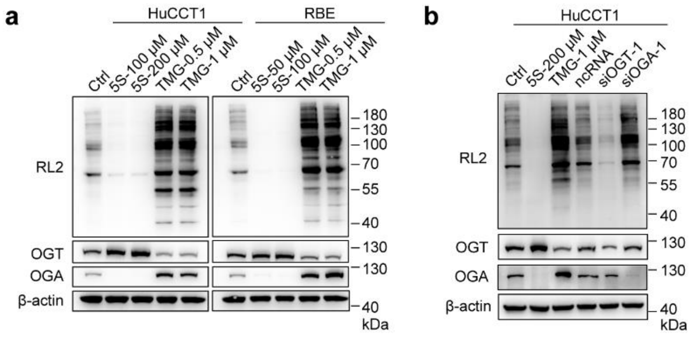

Figure S12. Global *O*-GlcNAcylation of the CCA cells is affected by regulating OGT and OGA levels. (a-b) Western blot analysis of the *O*-GlcNAcylated protein, OGT, and OGA levels in 5S- or TMG-treated (a) and 5S-, TMG-, siOGT-1, or siOGA-1 treated (b) HuCCT1 and RBE cells. Equal loading was confirmed using β-actin.

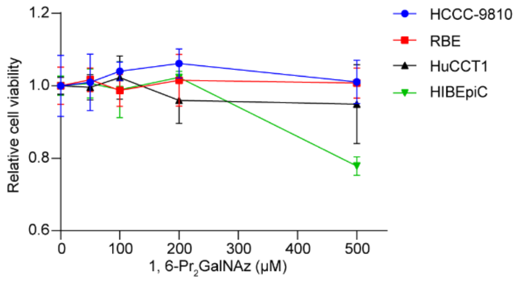

Figure S13. Cytotoxicity of 1,6-Pr_2GalNAz in CCA and HIBEpiC cells by CCK-8 assays. 1,6-Pr_2GalNAz, 1,6-di-*O*-propionyl-*N*-azidoacetylgalactosamine. Data were shown as the mean ± SD.

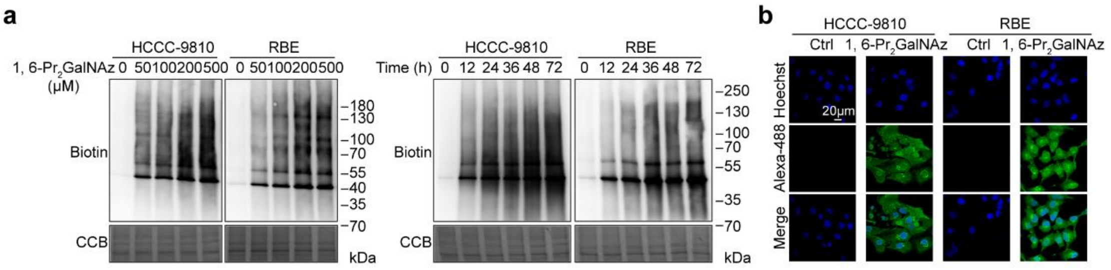

Figure S14. Metabolic efficacy of 1,6-Pr_2GalNAz in HCCC-9810 and RBE cells. (a) Western blot analysis of HCCC-9810 and RBE cells treated with 1,6-Pr_2GalNAz at various concentrations for different times. The cell lysates were reacted with alkyne-biotin via copper (I)-catalyzed azide-alkyne cycloaddition (CuAAC) and blotted using antibiotin. Equal loading was confirmed using Coomassie brilliant blue staining (CBB). (b) Confocal fluorescence imaging of HCCC-9810 and RBE cells treated with 1,6-Pr_2GalNAz at 0 or 200 μM for 48 h. The cells were washed, labeled with alkyne-AZDye-488, and analyzed. Scale bar: 20 μm.

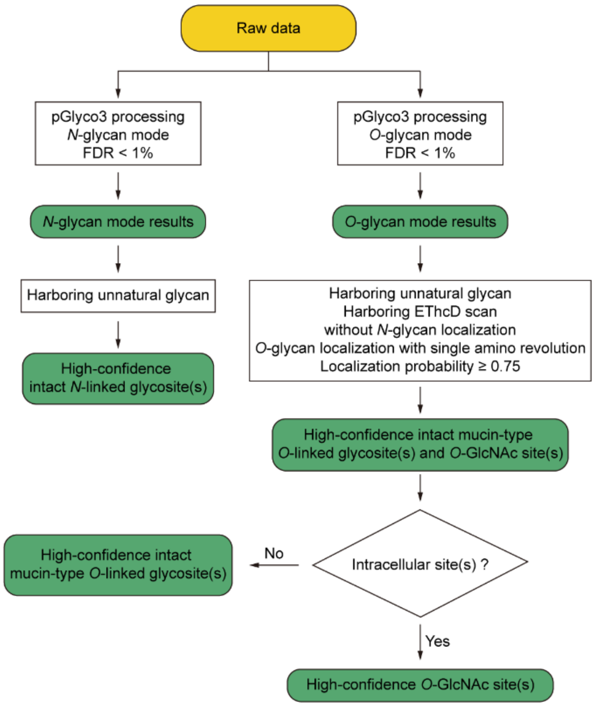

Figure S15. Streamlined pipeline for screening high-confidence intact glycosites.

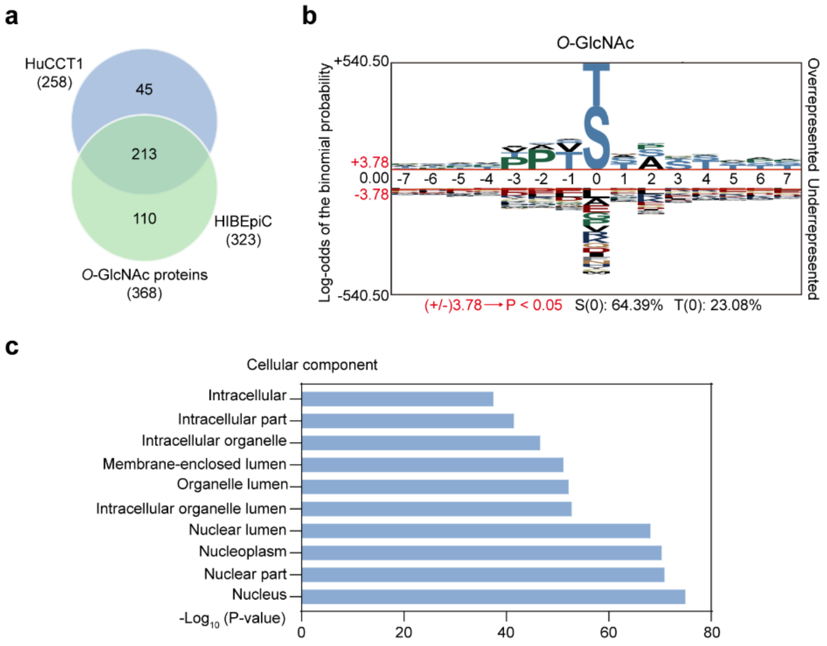

Figure S16. Profiling of protein *O*-GlcNAcylation in HuCCT1 and HIBEpiC cells. (a) Overlap of the identified *O*-GlcNAcylated proteins between HuCCT1 and HIBEpiC cells. (b) Motif analysis of *O*-GlcNAc sites identified in HuCCT1 and HIBEpiC cells using the pLogo. The log-odds binomial probability and adjusted P-value (adjusted by a conservative Bonferroni correction) were shown. (c) Cellular component gene ontology (GO) terms of the identified *O*-GlcNAcylated proteins in HuCCT1 and HIBEpiC cells using the Database for Annotation, Visualization and Integrated Discovery (DAVID). The annotation terms and enrichment P-values (adjusted by a modified Fisher's exact test) were shown.

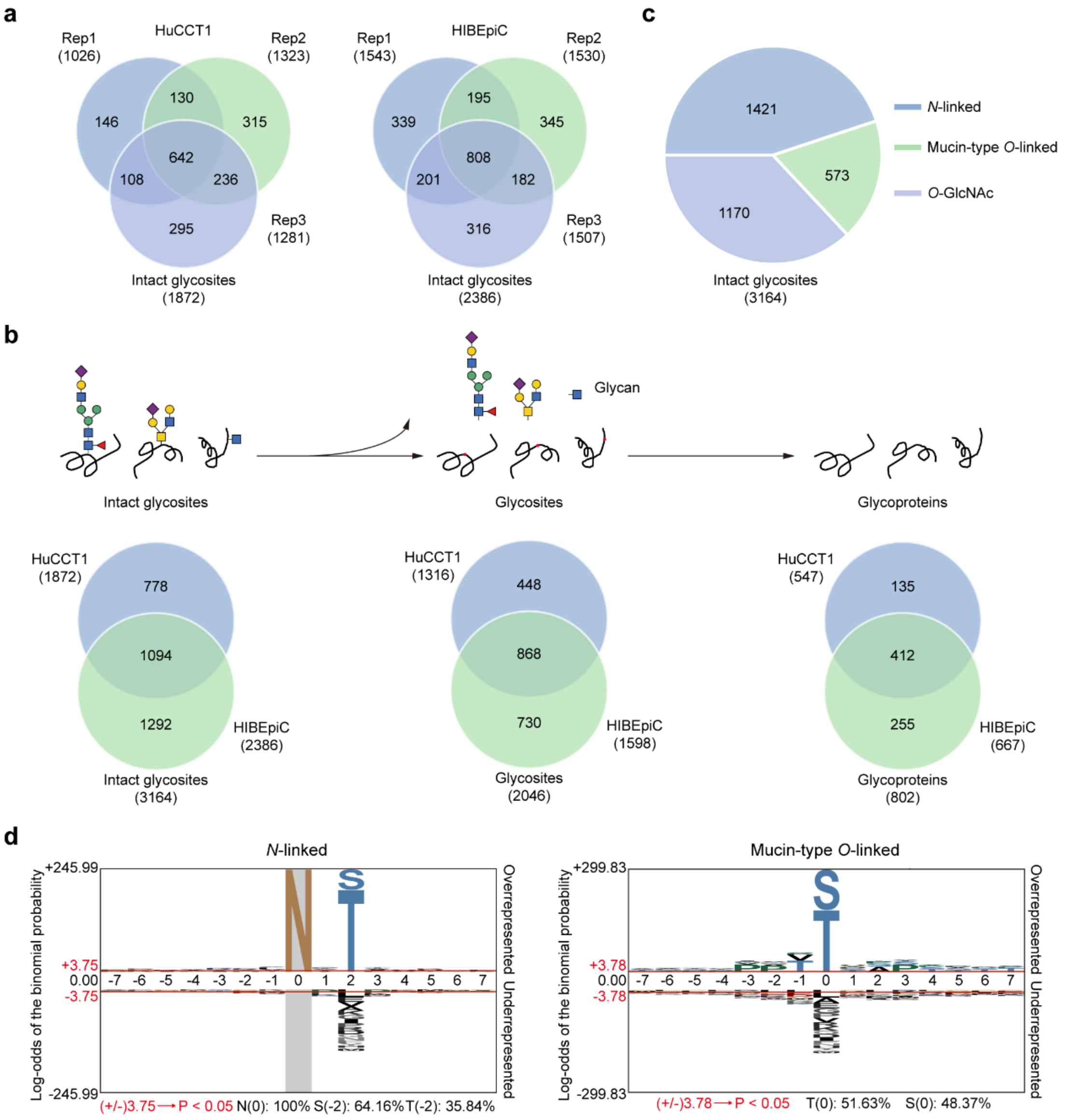

Figure S17. Profiling of the intact glycosites in HuCCT1 and HIBEpiC cells. (a) Overlap of the identified intact glycosites in HuCCT1 or HIBEpiC cells between three replicates. (b) Overlap of the identified intact glycosites, glycosites, and glycoproteins between HuCCT1 and HIBEpiC cells. (c) Total numbers and percentages of the intact *N*-linked glycosites, intact mucin-type *O*-linked glycosites, and intact *O*-GlcNAc sites identified in HuCCT1 and HIBEpiC cells. (d) Motif analysis of the *N*-linked glycosites and mucin-type *O*-linked glycosites identified in HuCCT1 and HIBEpiC cells using the pLogo. The log-odds binomial probability and adjusted *P*-value (adjusted by a conservative Bonferroni correction) were shown.

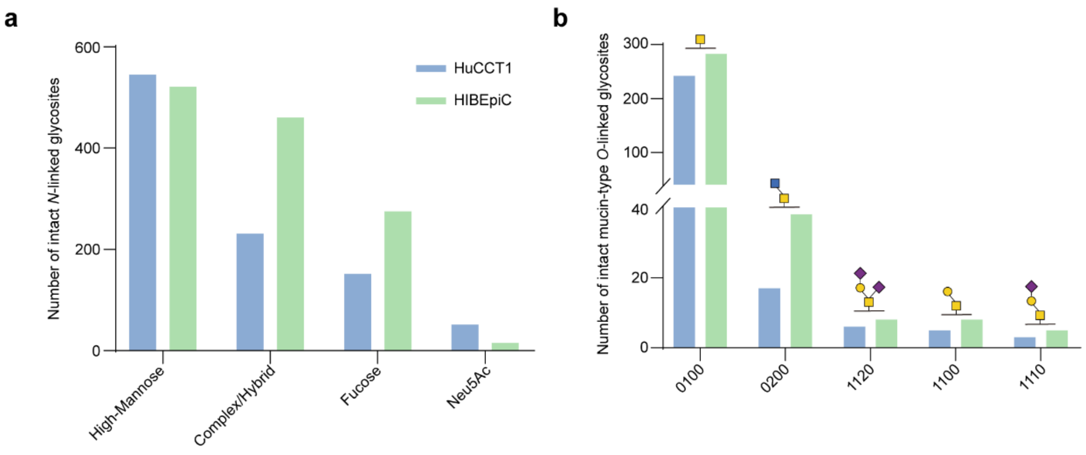

Figure S18. Numbers of the intact glycosites with specific glycan in HuCCT1 and HIBEpiC cells. (a) Numbers of the intact *N*-linked glycosites with specific types (High-Mannose, Complex/Hybrid, Fucose, and Neu5Ac) of *N*-glycans. (b) Numbers of the intact mucin-type *O*-linked glycosites with specific *O*-glycans.

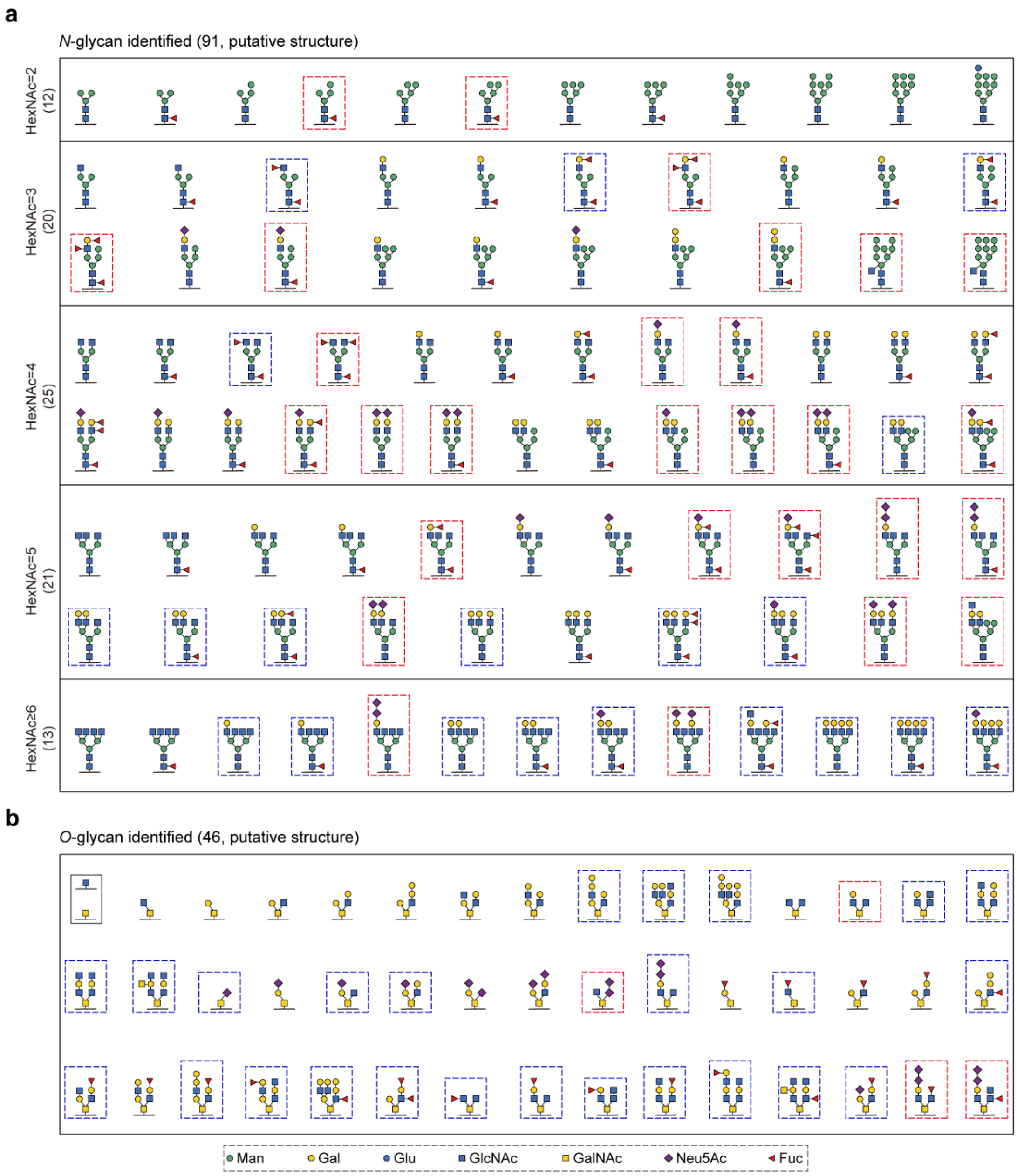

Figure S19. Glycan compositions from the intact glycosites in HuCCT1 and HIBEpiC cells. (a) The putative glycan structure of 91 *N*-glycans was shown. (b) The putative glycan structure of *O*-GlcNAc and 45 mucin-type *O*-glycans was shown. The red or blue boxes were identified only in HuCCT1 or HIBEpiC cells, respectively.

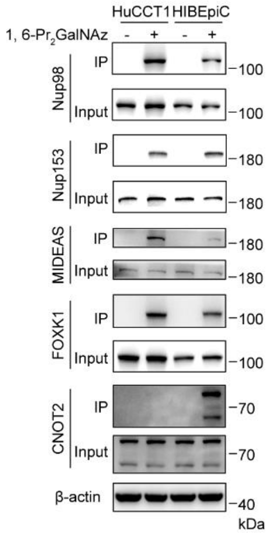

Figure S20. Biochemical validation of identified *O*-GlcNAcylated proteins in HuCCT1 and HIBEpiC cells. Western blot analysis showing the *O*-GlcNAcylated candidates (Nup98, Nup153, MIDEAS, FOXK1, and CNOT2) in HuCCT1 and HIBEpiC cells. The cells incubated with 1,6-Pr_2GalNAz, lysed, reacted with alkyne-biotin, and captured by streptavidin beads. Equal loadings were confirmed using β-actin.

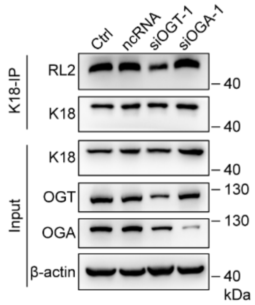

Figure S21. Knockdown of *OGT* reduces the *O*-GlcNAcylated K18 protein levels. Western blot and immunoprecipitation analysis showing the *O*-GlcNAcylated K18, K18, OGT, and OGA protein levels in HuCCT1 cells transfected with ncRNA, siOGT-1, or siOGA-1. Equal loadings were confirmed using β-actin.

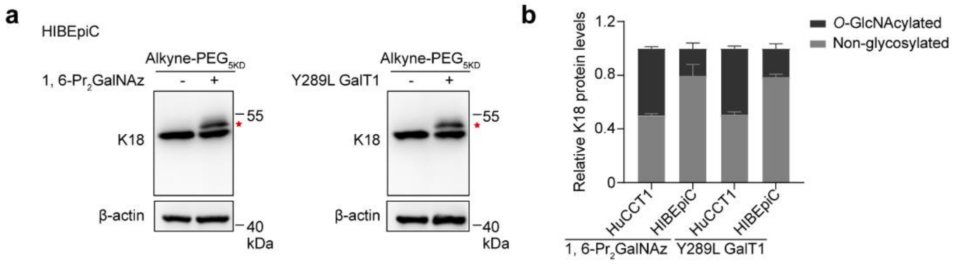

Figure S22. *O*-GlcNAcylation stoichiometry of K18 by Western blot analysis. (a) The HiBEpiC cells were incubated with 1,6-Pr_2GalNAz, lysed, reacted with alkyne-PEG_{5KD} (left), or incubated with Y289L GalT1 and UDP-GalNAz in cell lysates, reacted with alkyne-PEG_{5KD} (right). The red asterisk indicated tagged *O*-GlcNAcylated K18. (b) Relative *O*-GlcNAcylated and nonglycosylated K18 levels in HuCCT1 and HIBEpiC cells. Data were shown as the mean ± SD.

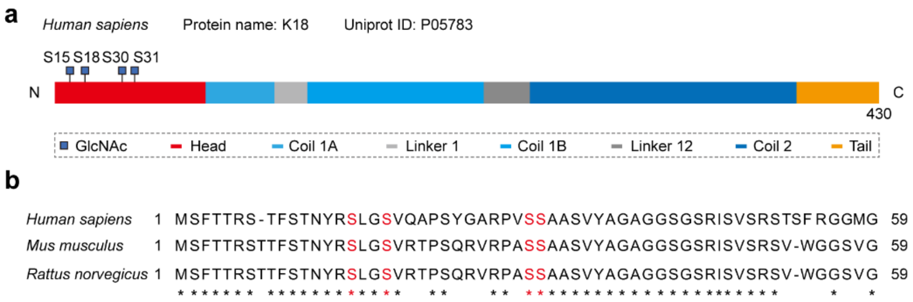

Figure S23. *O*-GlcNAc sites of K18 identified in HuCCT1 and HIBEpiC cells. (a) Schematic showing the *O*-GlcNAc sites of K18. (b) The *O*-GlcNAc sites of K18 are conserved across different species. *Human sapiens* (NP_001191439.1), *Mus musculus* (NP_001152847.1), *Rattus norvegicus* (NP_001093628.1).

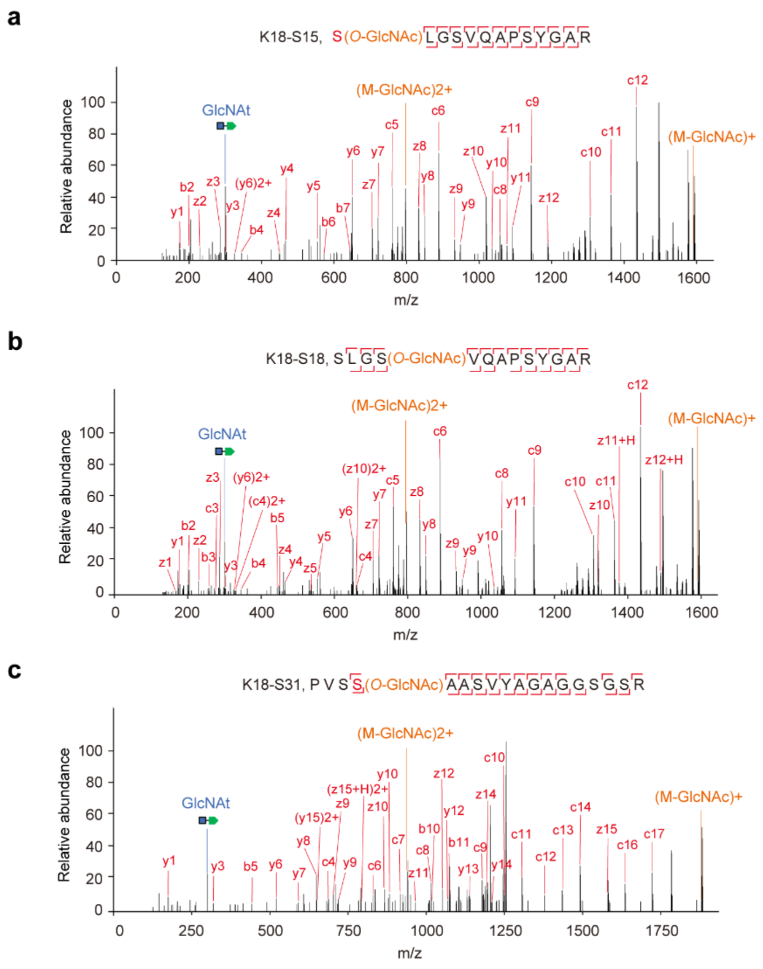

Figure S24. MS2 spectrum analysis of *O*-GlcNAcylated peptide in K18. (a-c) Representative MS2 spectrum of an *O*-GlcNAcylated peptide from K18 located on Ser 15 (a), Ser 18 (b), and Ser 31 (c). The matched fragment ions (red), diagnostic fragment ion (orange), and the GlcNAt fragment ion (blue) are labeled.

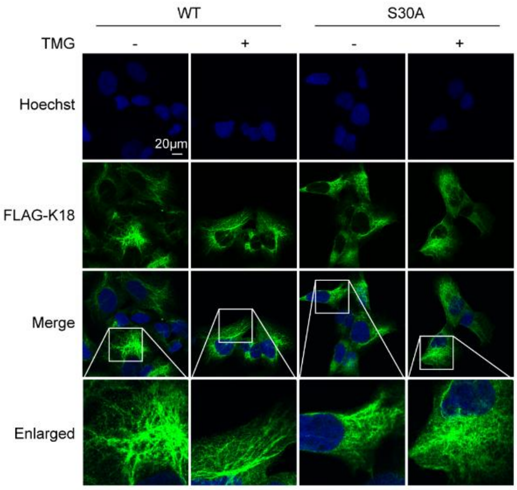

Figure S25. Confocal fluorescence imaging of K18 filament organization in RBE cells. Scale bar: 20 μm.

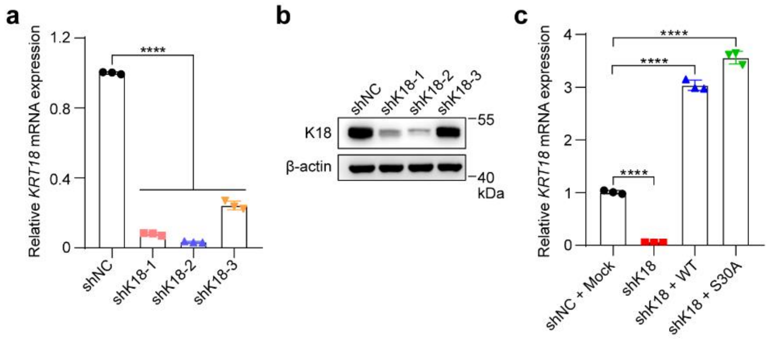

Figure S26. Generation of HuCCT1 stable cell lines. (a) The *KRT18* mRNA expression of HuCCT1 stable cell lines with small hairpin RNA K18 knockdown (shK18-1~3) by qRT-PCR. Random small hairpin RNA (shNC) was used as a negative control. (b) Western blot analysis of K18 in HuCCT1 stable cell lines with shK18-1~3. Equal loadings were confirmed using β-actin. (c) qRT-PCR analysis of the *KRT18* mRNA expression in HuCCT1 stable cell lines with small hairpin RNA K18 knockdown (shK18) and re-expression of shK18-resistant FLAG-K18 wild-type (shK18 + WT) or FLAG-K18 S30A (shK18 + S30A). Random small hairpin RNA with an empty vector (shNC + Mock) was used as a negative control. Data were shown as the mean ± SD; statistical significance was determined by Student's *t* tests (two-tailed, ****$P < 0.0001$).

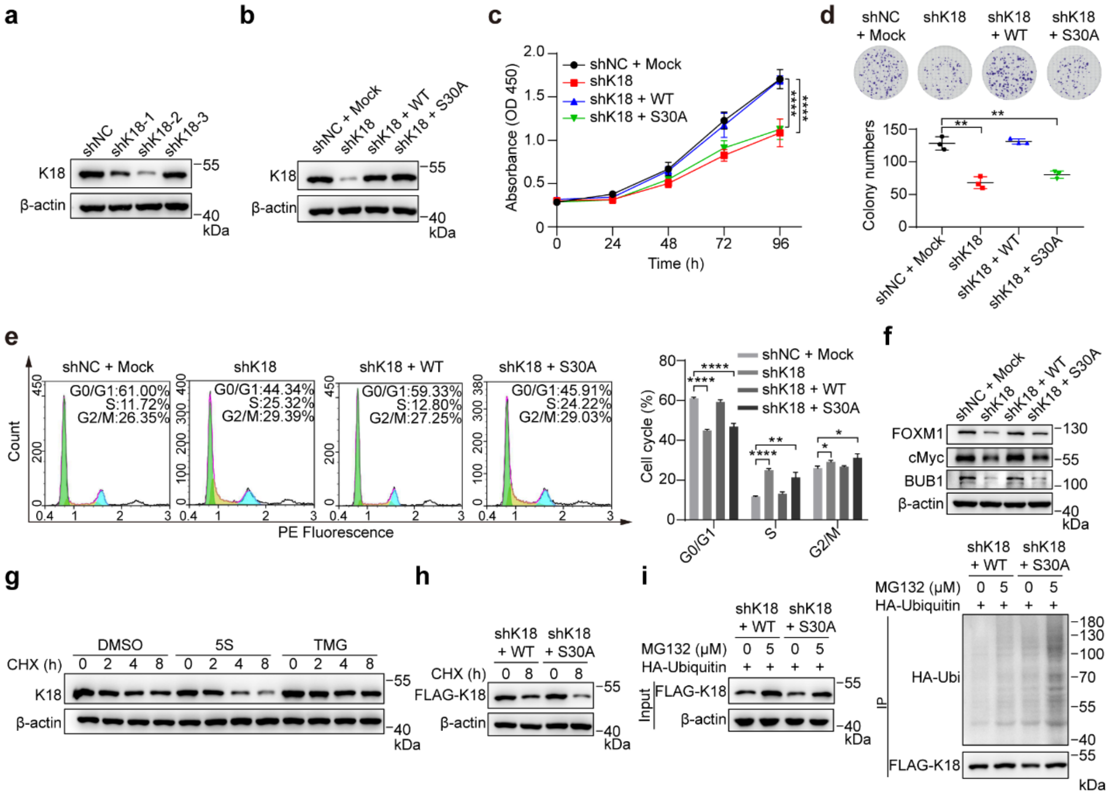

Figure S27. *O*-GlcNAcylation of K18 promotes cell growth in RBE cells. (a) Western blot analysis of K18 in RBE stable cell lines with shK18-1~3. (b) Western blot analysis of K18 in RBE stable cell lines with small hairpin RNA K18 knockdown (shK18) and re-expression of shK18-resistant FLAG-K18 wild-type (shK18 + WT) or FLAG-K18 S30A (shK18 + S30A). Random small hairpin RNA with an empty vector (shNC + Mock) was used as a negative control. (c) CCK-8 analysis of RBE stable cell lines. Absorbance was measured for cell viability. (d) Clonogenic assay of cell proliferation in RBE stable cell lines. Colony numbers were quantitatively analyzed at the bottom. (e) Cell cycle distribution assays of RBE stable cell lines. Histogram plot in flow cytometry indicated the percentage of cell populations in the G0/G1, S, or G2/M phase. Quantitative analysis was shown in the right panel. (f) Cell cycle marker analysis of RBE stable cell lines by Western blot. Protein levels of FOXM1, cMyc (G1/S transition markers), and BUB1(G2/M transition marker) were analyzed. (g) Degradation analysis of K18 in RBE cells analyzed by Western blot. The cells were incubated with DMSO (vehicle), 200 μM 5S, or 1 μM TMG for 48 h, followed by treatment with 10 μg/mL

CHX for up to 8 h. (h) Degradation analysis of K18 in RBE shK18 + WT or shK18 + S30A stable cell lines by Western blot. The cells were incubated with DMSO (vehicle) or 10 μg/mL CHX for 8 h. (i) Ubiquitination analysis of K18 in RBE shK18 + WT or shK18 + S30A stable cell lines. The cells were transfected with HA-ubiquitin, incubated with 5 μM MG-132 (proteasome inhibitor) for 20 h, lysed, and captured with anti-FLAG beads (left). Anti-HA blot demonstrated the ubiquitination of immunoprecipitated FLAG-K18 (right). Equal loadings were confirmed using β-actin in all Western blot analyses. Data were shown as the mean ± SD; statistical significance was determined by Student's *t* tests (two-tailed, $^*P < 0.05$, $^{**}P < 0.01$ and $^{****}P < 0.0001$).

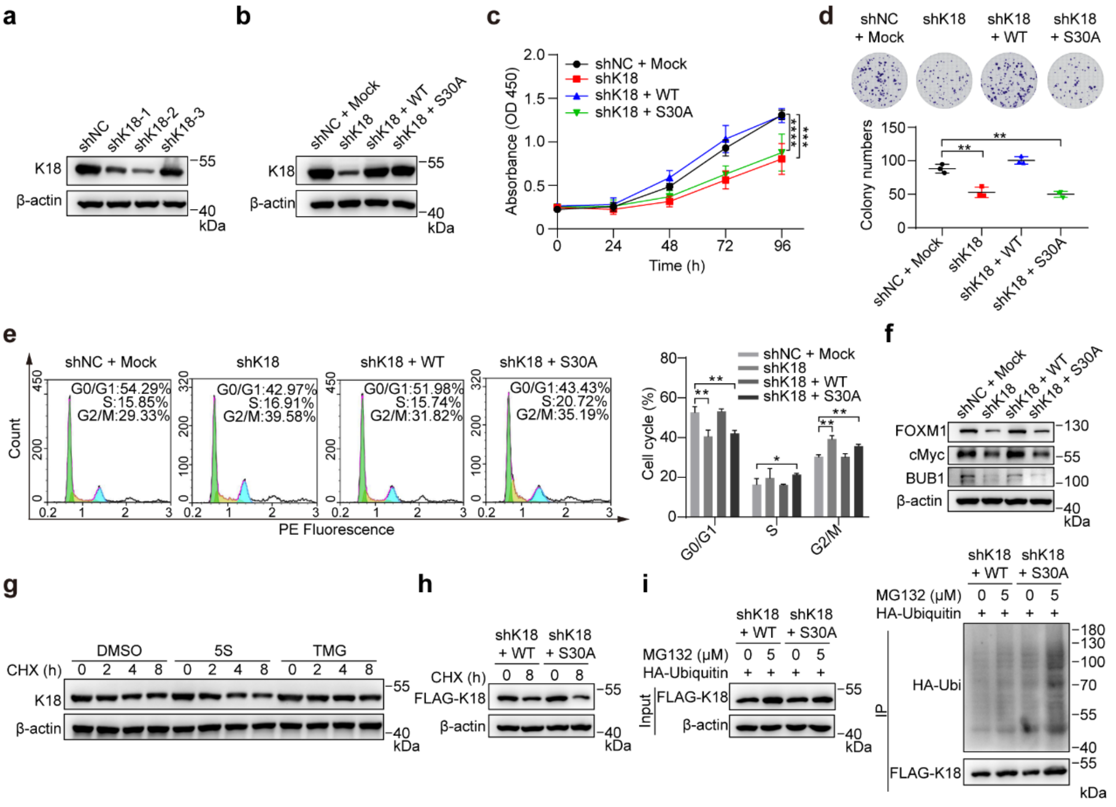

Figure S28. *O*-GlcNAcylation of K18 promotes cell growth in HCCC-9810 cells. (a) Western blot analysis of K18 in HCCC-9810 stable cell lines with shK18-1~3. (b) Western blot analysis of K18 in HCCC-9810 stable cell lines with small hairpin RNA K18 knockdown (shK18) and re-expression of shK18-resistant FLAG-K18 wild-type (shK18 + WT) or FLAG-K18 S30A (shK18 + S30A). Random small hairpin RNA with an empty vector (shNC + Mock) was used as a negative control. (c) CCK-8 analysis of HCCC-9810 stable cell lines. Absorbance was measured for cell viability. (d) Clonogenic assay of cell proliferation in HCCC-9810 stable cell lines. Colony numbers were quantitatively analyzed at the bottom. (e) Cell cycle distribution assays of HCCC-9810 stable cell lines. Histogram plot in flow cytometry indicated the percentage of cell populations in the G0/G1, S, or G2/M phase. Quantitative analysis was shown in the right panel. (f) Cell cycle marker analysis of HCCC-9810 stable cell lines by Western blot. Protein levels of FOXM1, cMyc (G1/S transition markers), and BUB1(G2/M transition marker) were analyzed. (g) Degradation analysis of K18 in HCCC-9810 cells analyzed by Western blot. The cells were incubated with DMSO (vehicle), 200 μM 5S,

or 1 μM TMG for 48 h, followed by treatment with 10 μg/mL CHX for up to 8 h. (h) Degradation analysis of K18 in HCCC-9810 shK18 + WT or shK18 + S30A stable cell lines by Western blot. The cells were incubated with DMSO (vehicle) or 10 μg/mL CHX for 8 h. (i) Ubiquitination analysis of K18 in HCCC-9810 shK18 + WT or shK18 + S30A stable cell lines. The cells were transfected with HA-ubiquitin, incubated with 5 μM MG-132 (proteasome inhibitor) for 20 h, lysed, and captured with anti-FLAG beads (left). Anti-HA blot demonstrated the ubiquitination of immunoprecipitated FLAG-K18 (right). Equal loadings were confirmed using β-actin in all Western blot analyses. Data were shown as the mean ± SD; statistical significance was determined by Student's *t* tests (two-tailed, $^*P < 0.05$, $^{**}P < 0.01$, $^{***}P < 0.001$ and $^{****}P < 0.0001$).

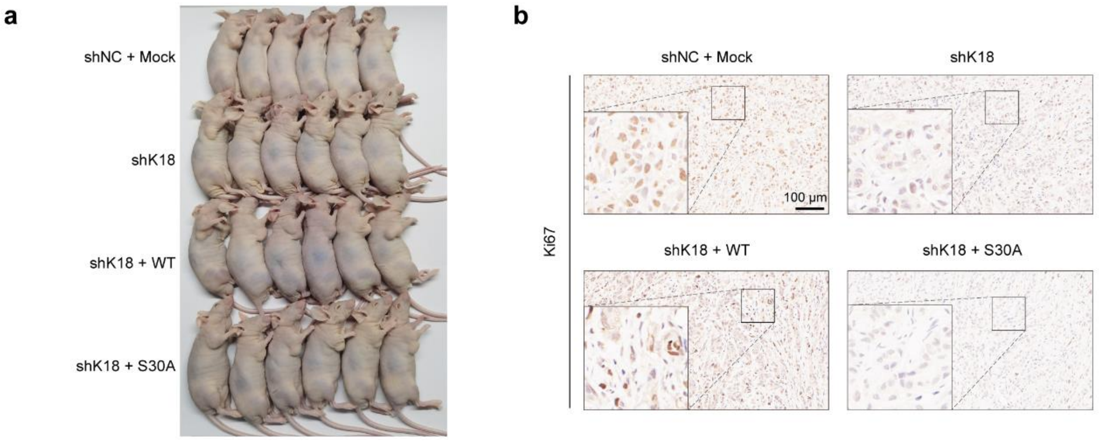

Figure S29. Tumor formation in nude mice. (a) Photograph of tumors generated by xenograft HuCCT1 stable cell lines. (b) Representative images of Ki67 staining in xenograft tumors through immunohistochemistry analysis. Scale bar: 100 μm.

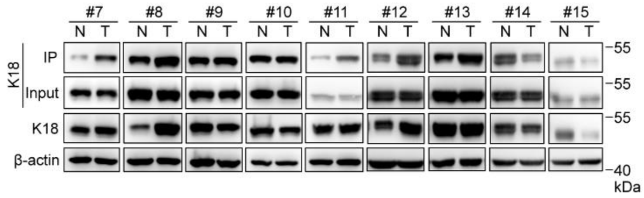

Figure S30. K18 and its *O*-GlcNAcylation are dysregulated in CCA. Representative images of K18 and its *O*-GlcNAcylation levels from nine pairs of CCA tumor tissues (T) and adjacent normal tissues (N) by Western blot analysis. Equal loadings were confirmed using β-actin.

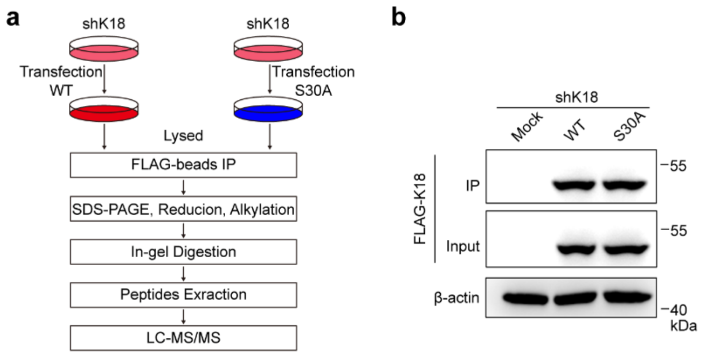

Figure S31. Analysis of interacting proteins of K18 protein. (a) Workflow of the analysis of interacting proteins with K18 in HuCCT1 shK18 cells transfected with FLAG-K18^{WT} or FLAG-K18^{S30A} by LC-MS/MS. (b) Western blot and immunoprecipitation analysis showing the FLAG-K18 protein levels in HuCCT1 shK18 cells transfected with FLAG-K18^{WT} or FLAG-K18^{S30A}. Equal loadings were confirmed using β-actin.

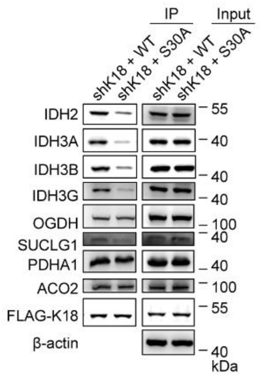

Figure S32. Analysis of enzymes in the TCA cycle interacting with K18 of RBE stable cell lines. Protein levels of IDH2, IDH3A, IDH3B, IDH3G, OGDH, SUCLG1, PDHA1, ACO2, and FLAG-K18 were analyzed by Western blot and immunoprecipitation analysis. Equal loadings were confirmed using β-actin.

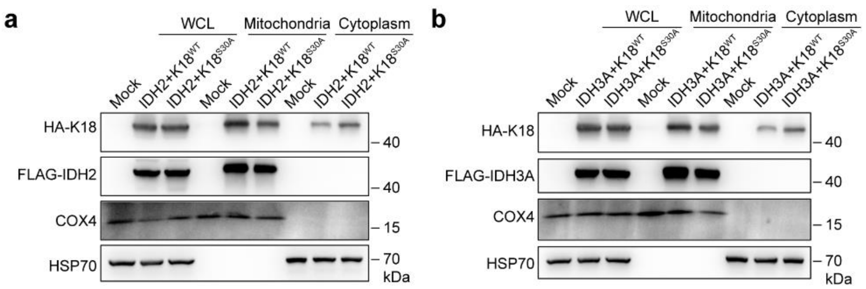

Figure S33. Distribution of K18 protein in CCA cells. (a-b) RBE cells cotransfected with FLAG-IDH2 and HA-K18^{WT} or HA-K18^{S30A} (a), as well as FLAG-IDH3A and HA-K18^{WT} or HA-K18^{S30A} (b), were homogenized and subjected to subcellular fractionation, followed by immunoblotting analysis for cellular distribution of HA-K18, FLAG-IDH2 or FLAG-IDH3A. COX4 and HSP70 were used as mitochondrial and cytoplasmic markers, respectively.

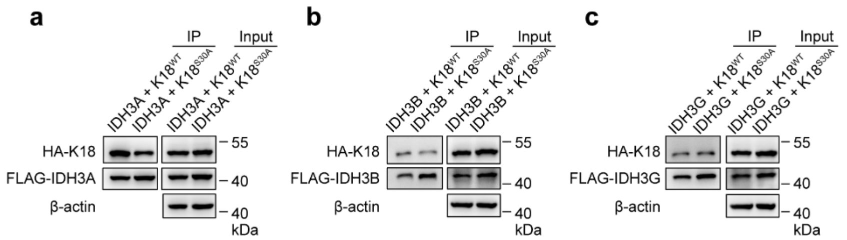

Figure S34. Analysis of IDH(s) interacted with K18 in CCA cells. (a-c) RBE cells cotransfected with FLAG-IDH3A and HA-K18^{WT} or HA-K18^{S30A} (a), FLAG-IDH3B and HA-K18^{WT} or HA-K18^{S30A} (b), as well as FLAG-IDH3G and HA-K18^{WT} or HA-K18^{S30A} (c), followed by lysing and anti-FLAG immunoprecipitation. Western blot and immunoprecipitation analysis showing the HA-K18 and FLAG-IDH(s) protein levels. Equal loadings were confirmed using β-actin in all Western blot analyses.

a

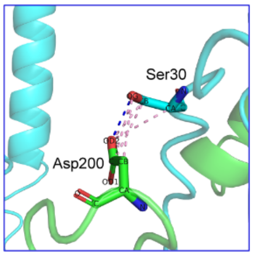

K18^{WT} Interaction site	IDH2 Interaction site	Distance (Å)	Interaction type
Ser30 (OG)	Asp200 (OD2)	2.69	Hydrogen bond
Ser30 (CA)	Asp200 (OD2)	3.69	Nonbonded contact
Ser30 (CB)	Asp200 (CG)	3.82	Nonbonded contact
Ser30 (CB)	Asp200 (OD2)	2.84	Non-bonded contact
Ser30 (OG)	Asp200 (CB)	3.72	Nonbonded contact
Ser30 (OG)	Asp200 (CG)	3.57	Nonbonded contact
Ser30 (OG)	Asp200 (OD2)	2.69	Nonbonded contact

b

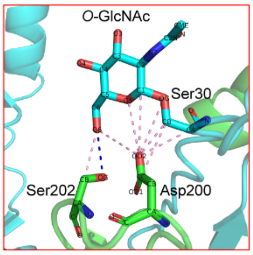

K18^{WT} (*O*-GlcNAc) Interaction site	IDH2 Interaction site	Distance (Å)	Interaction type
O-GlcNAc (O6)	Ser202 (OG)	3.07	Hydrogen bond
O-GlcNAc (O5)	Asp200 (OD2)	3.65	Nonbonded contact
O-GlcNAc (C1)	Asp200 (OD2)	3.81	Nonbonded contact
O-GlcNAc (O6)	Ser202 (CB)	3.23	Nonbonded contact
O-GlcNAc (O6)	Asp200 (OD2)	3.07	Nonbonded contact
Ser30 (CA)	Asp200 (OD2)	3.45	Nonbonded contact
Ser30 (CB)	Asp200 (CG)	3.58	Nonbonded contact
Ser30 (CB)	Asp200 (OD2)	2.60	Nonbonded contact
Ser30 (OG)	Asp200 (OD2)	3.75	Nonbonded contact

Figure S35. Structural modeling and simulation of IDH2-nonglycosylated K18 binding and IDH2-glycosylated K18 (*O*-GlcNAcylated at Ser30) binding. The matched hydrogen bond (blue) and nonbonded contact (pink) are labeled.

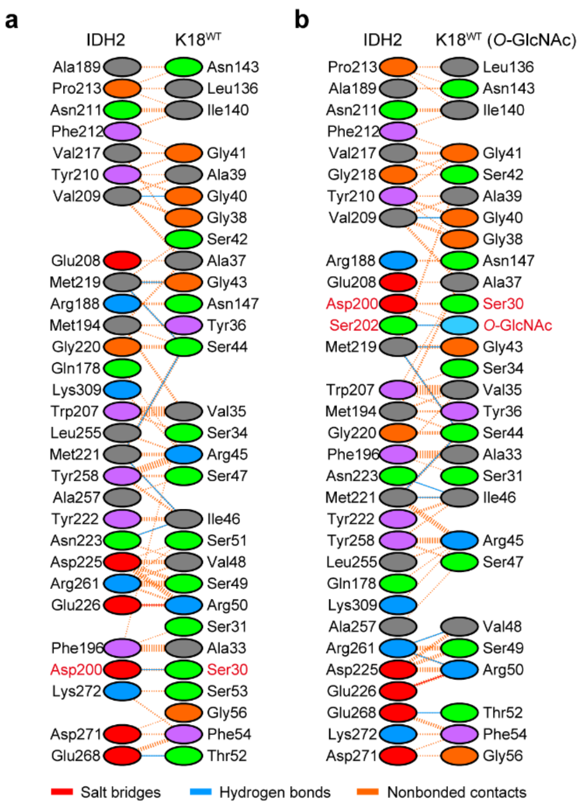

Figure S36. Schematic diagrams of the interactions for IDH2-nonglycosylated K18 binding and IDH2-glycosylated K18 (*O*-GlcNAcylated at Ser30) binding. The matched salt bridge (red), hydrogen bond (blue), and nonbonded contact (orange) are labeled.

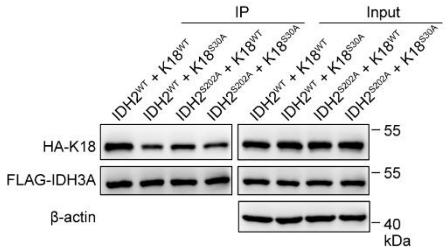

Figure S37. Ser202 of IDH2 contributes to its interaction with K18 in CCA cells. Western blot and immunoprecipitation analysis showing the HA-K18 and FLAG-IDH2 protein levels in cotransfected RBE cells (IDH2^{WT} + K18^{WT}, IDH2^{WT} + K18^{S30A}, IDH2^{S30A} + K18^{WT} and IDH2^{S202A} + K18^{S30A}), following with lysing and enrichment using anti-FLAG beads. Equal loadings were confirmed using β-actin.

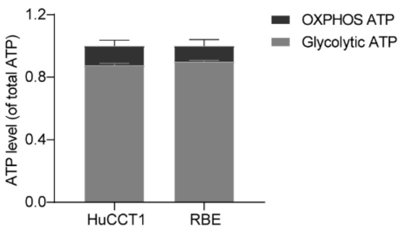

Figure S38. Measurement of glycolytic and oxidative phosphorylation (OXPHOS) ATP levels in CCA cells. Data were shown as the mean ± SD.

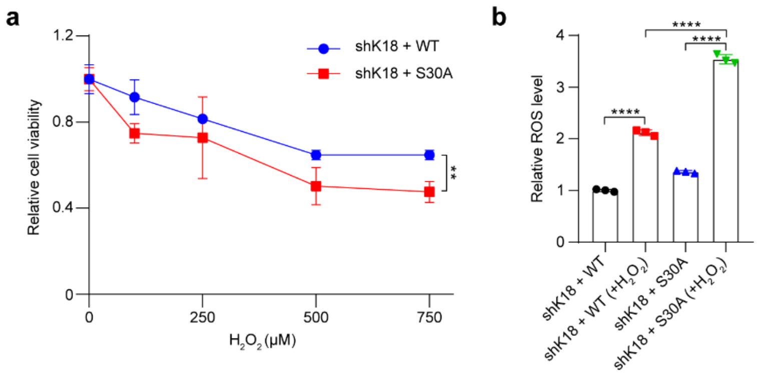

Figure S39. *O*-GlcNAcylation of K18 contributes to the resistance of H_2O_2 stimulation in CCA cells. (a) Cell viability assay of HuCCT1 stable cell lines (shK18 + WT and shK18 + S30A) treated with H_2O_2 at indicated concentrations for 12 h. (b) Relative ROS levels of HuCCT1 stable cell lines (shK18 + WT and shK18 + S30A) after treating with or without 500 μM H_2O_2 for 12 h. Data were shown as the mean ± SD; statistical significance was determined by Student's *t* tests (two-tailed, $^{**}P < 0.01$ and $^{****}P < 0.0001$).
