# Supplementary material for: O-GlcNAcylation Facilitates the Interaction between Keratin 18 and Isocitrate Dehydrogenases and Potentially Influencing Cholangiocarcinoma Progression
Source: ACS Cent Sci. 2024 Apr 23;10(5):1065–83. doi: 10.1021/acscentsci.4c00163 (PMC11117311; doi:10.1021/acscentsci.4c00163)
Supplement: Supplementary file 6 — oc4c00163_si_006.pdf [file oc4c00163_si_006.pdf]

oc-2024-00163q.R1

Name: Peer Review Information for "O-GlcNAcylation Facilitates the Interaction between Keratin 18 and Isocitrate Dehydrogenases and Potentially Influencing Cholangiocarcinoma Progression"

First Round of Reviewer Comments

Reviewer: 1

Comments to the Author

This is a very comprehensive study on the role of O-GlcNAc in Cholangiocarnioma. The authors perform a nice proteomics study to identify potential proteins that are differentially O-GlcNAc modified in disease versus healthy tissue. They then zero in on K18 glycosylation and perform a series of nice experiments chasing down potential mechanisms, including stability and interactions with cellular metabolism mediated by K18 O-GlcNAc modification at serine 30. The experiments are quite comprehensive and overall paint a convincing picture. I support publication but have a few comments that should be addressed.

Comment #1 - It might be interesting to compare the absolute stoichiometry of K18 O-GlcNAc modification in the HuCCT1 versus HiBEpiC cells using mass shifting as in Figure 3b, since RL2 can miss some O-GlcNAc sites.

Comment #2 - I am intrigued by the potential mechanism where O-GlcNAc is involved in an additional hydrogen bond to stabilize an interaction between K18 and IDH2. Particularly because most functions of O-GlcNAc appear to be by inhibiting protein-protein interactions. I suggest that the authors test this mechanism by making mutations to Ser200 to see if the integration with K18 but not K18 S30A is decreased.

Comment #3 (minor) - On page 8, line 13, the authors refer to "Leloir-type glycosylatransferase OGT." I don't think this is a correct way to say what they are trying to say. The Leloir pathway refers to the metabolism of Gal/GalNAc and conversion to Glc/GlcNAc, not OGT.

Reviewer: 2

Comments to the Author

The manuscript by Meng et al. is a very nice study focusing on the role of O-GlcNAcylation in cholangiocarcinoma (CCA). They identified the global glycoproteomic landscape of O-GlcNAcylation in CCA using the recently developed Click-iG strategy, which combines metabolic labeling, clickable unnatural sugars, and chemoproteomic profiling. The authors then showed that keratin 18 (K18) is O-GlcNAcylated mainly at Ser 30, and that O-GlcNAcylation of K18 promotes CCA proliferation and progression in vitro and in vivo. They suggest a potential role for O-GlcNAcylation in the coordination of K18 and its interplay with isocitrate dehydrogenases (IDHs) to promote CCA progression. The results are interesting, the data are of high quality.

Main comments:

1. In the Introduction, the authors should briefly describe the advantages or improvements of the Click-iG strategy over other established strategies.

2. O-GlcNAcylation is involved in a variety of biological activities in cancer and its mechanism is very complex. In this manuscript, the authors show that O-GlcNAcylation of K18 plays a critical role in promoting CCA proliferation and progression. Why did the authors choose the K18 protein to study the role of O-GlcNAc modification in CCA?

3. Can O-GlcNAcylation be modulated to treat CCA?

Minor comments:

1. please comment on how many Cholangiocarcinoma patients are there worldwide.

2. Figure 1h, "Cleaved caspase3, cleaved PAPR, Bcl2, etc." are not right aligned as shown in Figure S10.

3. In line 17 on page 8, Bcl2 is an anti-apoptotic marker and the protein level is downregulated under 5S treatment.

4. Some subscripts in the figure appear to be formatted incorrectly, such as 1,6-Pr2GalNAz in Figure 2a, please check the full text and correct them.

Author's Response to Peer Review Comments:

Senior Editor

ACS Central Science 1155
Sixteenth Street N.W.
Washington, DC 20036,
USA

March 6, 2024

Dear Editor,

Thank you for providing us with two reviews for the manuscript entitled "*O*-GlcNAcylation Facilitates the Interaction between Keratin 18 and Isocitrate Dehydrogenases and Potentially Influencing Cholangiocarcinoma Progression" (Manuscript ID: oc-2024-00163q). The reviewers gave overall positive, inspiring, and insightful comments on the importance, and high level of interest in the topic, and made several helpful suggestions to improve both experimental aspects of our study as well as clarity.

In response to these comments, we are happy to submit a ***revised manuscript*** that addresses all of these considerations. The most salient changes to the manuscript are outlined below:

1. We have reorganized the revised manuscript to include necessary but overlooked literature citations throughout the paper, additionally emphasizing the background information about cholangiocarcinoma, as well as the core concepts and the innovation of the Click-iG strategy.

2. We performed several experiments we deemed necessary according to the reviewers' valuable comments. Several changes regarding to the Figures, especially typos, have also been made.

We also attached, along with the revised manuscript, the ***point-by-point responses***, and an ***annotated version of the manuscript*** for comparison convenience. We hope that with these changes, the manuscript will be now suitable for publication in *ACS Central Science.* We thank you for your precious time and expertise in handling our work. Of course, we would be happy to address any additional concerns or make any other amendments that you deem necessary.

Thank you for considering our revised paper.

Sincerely,

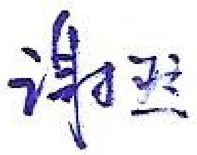

Dr. Ran Xie
School of Chemistry and Chemical Engineering,

National Key Laboratory of Coordination Chemistry,

Chemistry and Biomedicine Innovation Center (ChemBIC),
Nanjing University

Jiangsu, 210023, China

Point-by-point responses

This manuscript has been seen by two reviewers, and detailed review comments were provided. We find all review comments extremely supportive, inspiring and insightful. We have carefully considered each comment, and addressed all points with additional data to improve the overall quality of the manuscript. Below are our ***point-to-point responses*** to the comments and concerns raised by the reviewers. The cited, newly added or revised figures mentioned in the responses are shown correspondingly for the ease of your reference. We would like to acknowledge all reviewers for your precious time and efforts working on this manuscript. The manuscript file is now provided with both an annotated version with changes ***marked in blue***, and a formatted manuscript.

Referee:1

Review Comment 1:

"Recommendation: Publish in ACS Central Science after minor revisions noted.

Comments: This is a very comprehensive study on the role of O-GlcNAc in Cholangiocarnioma. The authors perform a nice proteomics study to identify potential proteins that are differentially O-GlcNAc modified in disease versus healthy tissue. They then zero in on K18 glycosylation and perform a series of nice experiments chasing down potential mechanisms, including stability and interactions with cellular metabolism mediated by K18 O-GlcNAc modification at serine 30. The experiments are quite comprehensive and overall paint a convincing picture. I support publication but have a few comments that should be addressed."

Response: We are grateful for the positive feedback and strong support from respected Reviewer 1. We acknowledge the reviewer for this concise yet insightful summary of the interesting points and key innovations in our work.

Reviewer comment 2:

"It might be interesting to compare the absolute stoichiometry of K18 O-GlcNAc modification in the
HuCCT1 versus HiBEpiC cells using mass shifting as in Figure 3b, since RL2 can miss some OGlcNAc sites."

Response: We thank the reviewer for this insightful suggestion. We conducted the mass shift assay for HiBEpiC cells accordingly and used the densitometric analysis for the blotting comparison (**Figure S22a**). In brief, the extent of *O*-GlcNAc modification on K18 was semi-quantitatively measured by labeling azides with alkynylated PEG 5000 (alkyne-PEG_{5KD}) in a mass shift assay. The stoichiometric ratio for *O*-GlcNAcylation was approximately 50 % in HuCCT1, and approximately 20 % in HiBEpiC (**Figure S22b**).

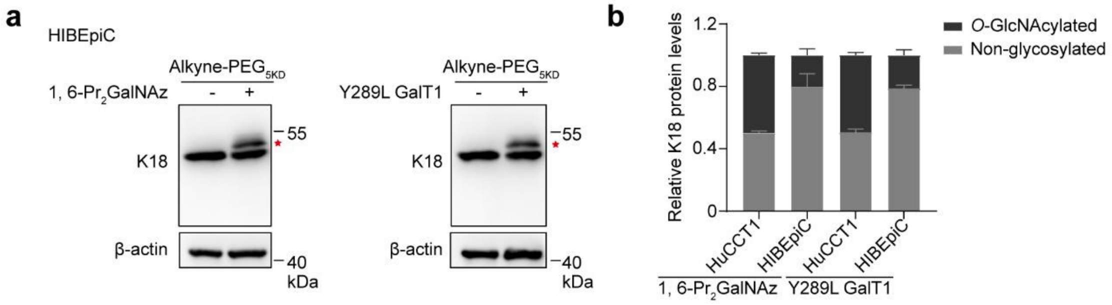

Figure S22. *O*-GlcNAcylation stoichiometry of K18 by Western blot analysis. (a) The HiBEpiC cells were incubated with 1,6-Pr_2GalNAz, lysed, reacted with alkyne-PEG_{5KD} (left), or incubated with Y289L GalT1 and UDP-GalNAz in cell lysates, reacted with alkyne-PEG_{5KD} (right). The red asterisk indicated tagged *O*-GlcNAcylated K18. (b) Relative *O*-GlcNAcylated and non-glycosylated K18 levels in HuCCT1 and HIBEpiC cells.

Reviewer comment 3:

"I am intrigued by the potential mechanism where O-GlcNAc is involved in an additional hydrogen bond to stabilize an interaction between K18 and IDH2. Particularly because most functions of OGlcNAc appear to be by inhibiting protein-protein interactions. I suggest that the authors test this mechanism by making mutations to Ser200 to see if the integration with K18 but not K18 S30A is decreased."

Response: We thank the reviewer for this interesting discussion and agree that additional data would definitely provide better scientific argumentation. Based on the docking results, *O*-GlcNAcylation on Ser 30 would majorly form the additional hydrogen bond with IDH2 at Ser 202 rather than Ser 200 (We logically assume that the respected reviewer made a typo in the comment). According to the interesting design made by the reviewer, we constructed FLAG-tagged IDH2 mutation plasmid (FLAGIDH2^{S202A}), and co-transfected with varied K18 wild-type and/or mutants, in RBE cells, a typical cholangiocarcinoma cell line. The experiment groups were labeled as IDH2^{WT}+K18^{WT}, IDH2^{WT}+K18^{S30A}, IDH2^{S30A}+K18^{WT}, and IDH2^{S202A}+K18^{S30A}. The samples were collected from the above-mentioned groups, lysed for total protein acquisition, enriched via anti-FLAG beads, and finally characterized by Western Blot and immunoprecipitation (**Figure S37**). As predicted by the reviewer, compared to IDH2^{WT} + K18^{WT}, the interaction between IDH2^{S202A} and K18^{WT} is decreasing, while compared to IDH2^{WT} + K18^{S30A}, the interaction between IDH2^{S202A} and K18^{S30A} did not exhibit further change. These data suggest that: 1) *the IDH2 Ser202 mutant integration is decreased with K18, but not K18 S30A.* 2) *The O-GlcNAcylation under this scenario stabilized this interaction via additional hydrogen bonding with Ser 202.*

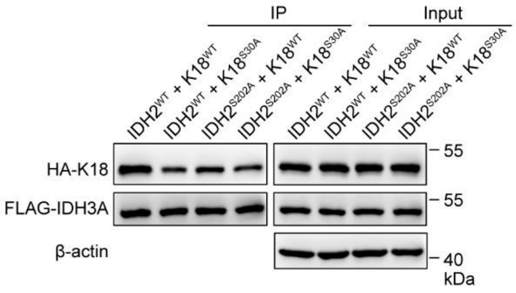

Figure S37. Ser202 of IDH2 contributes to its interaction with K18 in CCA cells. Western blot and immunoprecipitation analysis showing the HA-K18 and FLAG-IDH2 protein levels in co-transfected RBE cells (IDH2^{WT} + K18^{WT}, IDH2^{WT} + K18^{S30A}, IDH2^{S30A} + K18^{WT} and IDH2^{S202A} + K18^{S30A}), following with lysing and enrichment using anti-FLAG beads. Equal loadings were confirmed using β-actin. Data were shown as the mean ± SD.

Reviewer comment 4:

“On page 8, line 13, the authors refer to "Leloir-type glycosylatransferase OGT." I don't think this is a correct way to say what they are trying to say. The Leloir pathway refers to the metabolism of Gal/GalNAc and conversion to Glc/GlcNAc, not OGT.”

Response: We appreciate the professionalism our reviewer has put in improving the quality of the manuscript. We have fixed these phrases accordingly. In addition, language in both the main text and the Supporting Information has also been grammatically adjusted to eradicate the misunderstanding of our research.

Taken together, we wish to express our gratitude again to the respected Referee 1 for providing truly insightful suggestions.

Referee: 2

Reviewer comment 1:

“Recommendation: Publish in ACS Central Science after minor revisions noted.

Comments: The manuscript by Meng et al. is a very nice study focusing on the role of OGlcNAcylation in cholangiocarcinoma (CCA). They identified the global glycoproteomic landscape of O-GlcNAcylation in CCA using the recently developed Click-iG strategy, which combines metabolic labeling, clickable unnatural sugars, and chemoproteomic profiling. The authors then showed that keratin 18 (K18) is O-GlcNAcylated mainly at Ser 30, and that O-GlcNAcylation of K18 promotes CCA proliferation and progression in vitro and in vivo. They suggest a potential role for O-GlcNAcylation in the coordination of K18 and its interplay with isocitrate dehydrogenases (IDHs) to promote CCA progression. The results are interesting, the data are of high quality.”

Response: We are grateful for the positive feedback and strong support from the respected reviewer 2. We acknowledge the reviewer for this concise yet insightful summary of the interesting points, key innovations, and the amount of data in our work.

Reviewer comment 2:

“In the Introduction, the authors should briefly describe the advantages or improvements of the Click-iG strategy over other established strategies.”

Response: We thank the reviewer for this insightful suggestion for improving the quality of our manuscript. We therefore include a brief introduction of the Click-iG strategy as well as other glycoproteomic strategies in the Introduction section.

Reviewer comment 3:

“O-GlcNAcylation is involved in a variety of biological activities in cancer and its mechanism is very complex. In this manuscript, the authors show that O-GlcNAcylation of K18 plays a critical role in promoting CCA proliferation and progression. Why did the authors choose the K18 protein to study the role of O-GlcNAc modification in CCA?”

Response: We thank the reviewer for this interesting discussion. We identified *O*GlcNAcylated glycopeptides in the CCA cell line (HuCCT1) and control cell line (HIBEpiC) through the Click-iG strategy. Volcano plot analysis of *O*-GlcNAc sites in these two cells showed that the *O*-GlcNAcylation level at Ser30 of K18 protein was significantly upregulated in HuCCT1 cells (**Figure 2f**), which greatly attracted our attention. Additionally, K18 protein is an important member of the cytoskeletal protein family. We have introduced the pivotal roles of K18 protein in in intracellular scaffolding and cellular processes in the first paragraph of "Keratin 18 is mainly *O*-GlcNAcylated at Ser 30." in the Results and

Discussion section. We therefore chose the K18 protein to study the role of *O*GlcNAcylation in CCA.

Reviewer comment 4:

"Can O-GlcNAcylation be modulated to treat CCA?"

Response: We thank the respected reviewer for this question. In "Up-regulation of *O*GlcNAcylation in CCA is associated with its contribution to global cell proliferation in CCA cells." under the Results and Discussion section, we discovered that *O*-GlcNAcylation was significantly up-regulated in CCA tumor tissues and the CCA cell lines. Treatment of the cells with 5S (an OGT inhibitor) resulted in an overall decrease in intracellular *O*GlcNAcylation levels, while cells exhibited concentration-dependent cell death, whereas the opposite results were seen when cells were treated with TMG (an OGA inhibitor) (**Figure 1e, Figure S6**). Additionally, 5S-treated CCA cells also experienced cell cycle arrest and increased apoptosis (**Figure 1f-i, Figure S7, S8, S9, S10**). The decrease in *O*GlcNAcylation levels caused by the knockdown of OGT using siOGT also affected the proliferation of CCA cells (**Figure S11**). Based on these results, we are confident that CCA can be treated by modulating *O*-GlcNAcylation. Of course, this finding also pave the way for combinational therapy with O-GlcNAc inhibitors and clinical cancer drugs.

Reviewer comment 5:

"Please comment on how many Cholangiocarcinoma patients are there worldwide."

Response: We thank the reviewer for raising this important question with regard to the global incidence of CCA. CCA is a rare cancer, and includes a cluster of highly heterogeneous biliary malignant tumors that can arise at any point of the biliary tree. Their incidence is increasing

globally, currently accounting for ~15% of all primary liver cancers and ~3% of gastrointestinal malignancies. The silent presentation of these tumors combined with their highly aggressive nature and refractoriness to chemotherapy contribute to their alarming mortality, representing ~2% of all cancer-related deaths worldwide yearly. According to the report by Gores GJ. et al. in "Cholangiocarcinoma 2020: the next horizon in mechanisms and management",[1] the incidence (0.3-6 per 100,000 inhabitants per year) and mortality (1–6 per 100,000 inhabitants per year, globally, not taking into account specific regions with incidence >6 per 100,000 habitants such as South Korea, China, and Thailand) of CCA have been increasing in the past few decades worldwide, representing a global health problem.

Reviewer comment 6:

"Figure 1h, "Cleaved caspase3, cleaved PAPR, Bcl2, etc." are not right aligned as shown in Figure S10."

Response: We thank the reviewer for this insightful suggestion for improving the quality of our manuscript. We have formatted the text accordingly.

Reviewer comment 7:

"In line 17 on page 8, Bcl2 is an anti-apoptotic marker and the protein level is downregulated under 5S treatment."

Response: We have added this description at the corresponding position for clarification.

Reviewer comment 8:

"Some subscripts in the figure appear to be formatted incorrectly, such as 1,6-Pr_2GalNAz in Figure 2a, please check the full text and correct them."

Response: We apologize for the typo and thank the reviewer for pointing it out. We therefore proof-read all the figures in both the manuscript and supplementary information, and made several corrections accordingly, including $Ac_4$5SGlcNAc (**Figure 1a**), 1,6Pr_2GalNAz (**Figure 2a-d, Figure 3a, 3b, 3c, 3e, Figure S13, S14, S20**), Log_2 fold change

(**Figure 2f, Figure 5a**), Log_{10} (P-value) (**Figure 2f, Figure 5a, 5b, Figure S16**) and Alkyne-PEG_{5KD} (**Figure 3b**).

Taken together, we wish to express our gratitude again to the respected Referee 2 for providing truly insightful suggestions

In all, we wish to express our gratitude again to the two respected reviewers for the precious time, invaluable expertise, and superb professionalism you have put in improving the quality of our paper. We hope the manuscript is now suitable for the publication in ***ACS Central Science***.

References

(1) Banales, J. M.; Marin, J. J.; Lamarca, A.; Rodrigues, P. M.; Khan, S. A.; Roberts, L. R.; Cardinale, V.; Carpino, G.; Andersen, J. B.; Braconi, C.; et al. Cholangiocarcinoma 2020: the next horizon in mechanisms and management. *Nat. Rev. Gastroenterol. Hepatol.* **2020**, *17*, 557-588.
